# Unravelling the genomic epidemiology and antigenic evolution of Chandipura virus: A genetically constrained neurotropic threat

**DOI:** 10.1371/journal.pntd.0014565

**Published:** 2026-08-03

**Authors:** Pooja Rani Kuri, Amit Sharma

**Affiliations:** Molecular Medicine Group, International Centre for Genetic Engineering and Biotechnology (ICGEB), New Delhi, India; Oregon State University College of Veterinary Medicine, UNITED STATES OF AMERICA

## Abstract

Spanning almost six decades (1966–2024), 23 whole-genome sequences of Chandipura virus (CHPV), a neurotropic rhabdovirus associated with acute encephalitis primarily in India, have been analyzed. The virus is believed to be transmitted mainly by *Sergentomyia* and *Phlebotomus* sandflies. An epidemiological geographical map was developed showing global CHPV detection in sandflies, district-level human encephalitis reports in India, and sandfly species distribution across the country. This integrated landscape links viral occurrence with vector ecology and outbreak potential. To investigate evolutionary dynamics, we analyzed 23 complete genomes, comprising 5 human-derived isolates from India, 17 sandfly-derived isolates from Senegal and Kenya, and one hedgehog-derived isolate from Nigeria. Phylogenetic reconstruction revealed clear lineage segregation between Indian and African strains, indicating region-specific evolution. Despite this divergence, overall genomic variability remained low. Among all genes, the phosphoprotein exhibited relatively higher variability, while human-derived Indian sequences showed lower genetic diversity than the predominantly sandfly-derived African sequences; however, this comparison is confounded by differences in geography, sampling period, and host origin. Selection pressure analyses indicated predominantly purifying selection across coding regions. Episodic diversification analysis detected adaptive events in structural proteins. In the glycoprotein, all diversification sites (P272H, L424W, K503R) overlapped with predicted B-cell epitopes. In the matrix protein, both sites (K20R, D97N) mapped to predicted B-cell epitope regions. In contrast, among the two diversification sites in the nucleoprotein (N35E, A187V), only A187V overlapped with an epitope, whereas N35E was outside predicted antigenic domains. Collectively, these findings provide a comprehensive evolutionary and epidemiological perspective on CHPV and highlight the urgent need for systematic genomic surveillance to inform future diagnostic, vaccine and public health intervention strategies.

## Author summary

Chandipura virus causes severe and rapidly progressing brain infections in children, but many aspects of its spread and evolution remain unclear. In this study, we combined previously unanalyzed data types to understand better how this virus behaves across regions and hosts. We first created a map showing where human cases and sandfly detections have been reported in India and Africa, alongside the distribution of sandfly species. This helped us understand the ecological and geographical context of virus transmission. We then analyzed all available complete virus genomes and found that Indian and African viruses form two distinct groups, suggesting long-term separate evolution. Importantly, we identified genomic regions that showed signs of adaptation, some of which overlapped with immune-related sites in the virus. Our integrated approach provides new insight into how Chandipura virus spreads and evolves, which can support improved monitoring and preparedness efforts.

## Introduction

**Fig 1:**
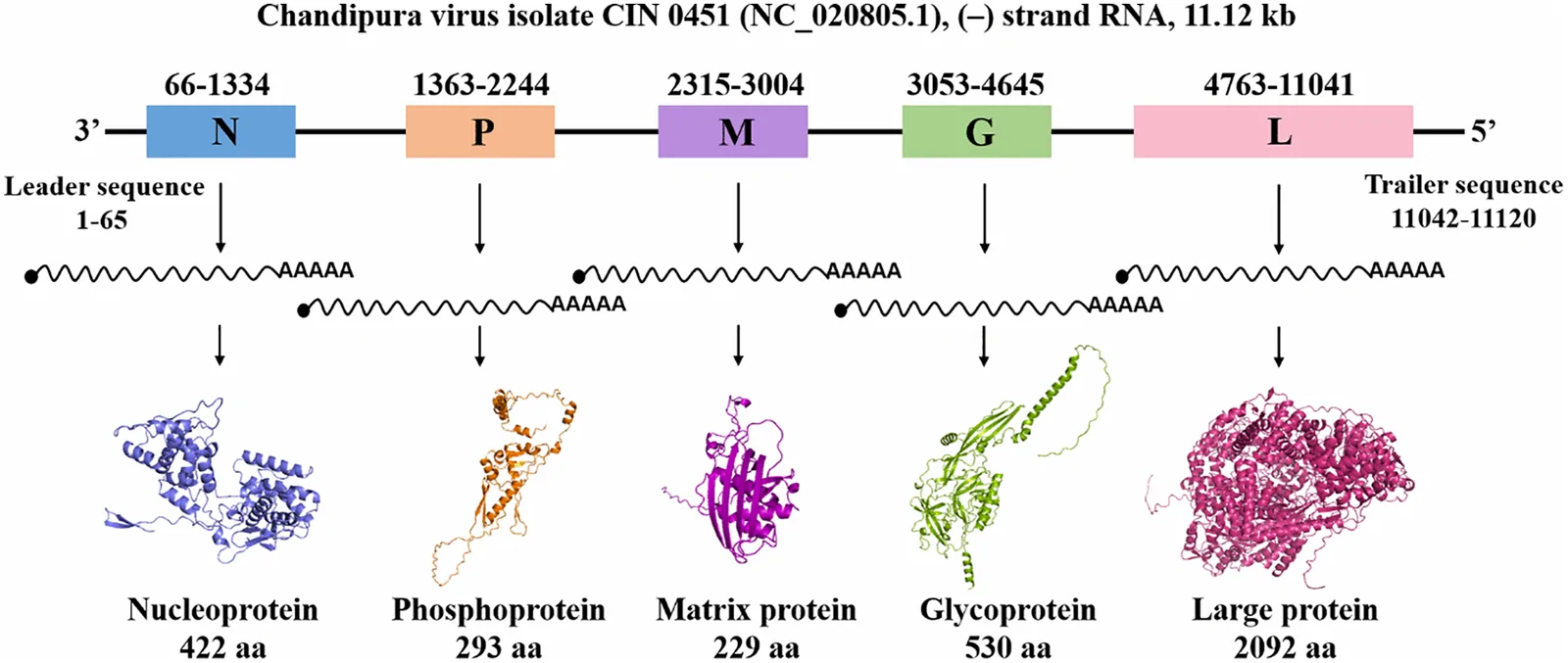
Genome organization and structural protein annotation of Chandipura virus. The schematic illustrates the genomic architecture of Chandipura virus (CHPV), a (–) sense single-stranded RNA genome of approximately 11.12 kb (RefSeq: NC_020805.1, isolate CIN 0451). The genome is depicted in the 3′ → 5′ orientation and annotated with the five canonical open reading frames corresponding to the nucleoprotein (N), phosphoprotein (P), matrix protein (M), glycoprotein (G), and RNA-dependent RNA polymerase (L).

CHPV is an enveloped, negative-sense, single-stranded RNA virus belonging to the family Rhabdoviridae and genus *Vesiculovirus*. Its ~ 11 kb genome follows the canonical rhabdovirus organization, 3′-N-P-M-G-L-5′, encoding five structural proteins with distinct roles in the viral life cycle [1]. The nucleoprotein (N) encapsidates and protects the viral RNA, forming the ribonucleoprotein complex which is essential for transcription and replication [2]. The phosphoprotein (P) functions as a cofactor of the polymerase complex, assisting in RNA synthesis and antagonizing host immune responses [3]. The matrix protein (M) mediates virion assembly, budding, and host cell shutoff mechanisms [4]. The glycoprotein (G) forms the surface spikes on the lipid envelope and facilitates receptor binding and membrane fusion during host cell entry [5]. The large RNA-dependent RNA polymerase (L) drives transcription and replication of the viral genome [6]. Fig 1 and Table 1 illustrate the genome annotation and protein attributes of CHPV. Together, these proteins enable efficient replication, immune evasion, assembly, and host-cell entry. Comparative genomics indicate that while CHPV shares structural and functional features with other *Vesiculoviruses*, it displays distinct pathogenic characteristics in humans (S1 Table). However, the molecular determinants underlying its neurotropism, host specificity, and vector competence, which are not yet fully characterized, remain to be elucidated.

**Table 1:** Genome annotation and protein attributes of Chandipura virus (isolate CIN0451, GenBank: NC_020805.1).

GenPept	Gene	Protein	Genomic Position (nt)	ORF Length (nt)	Protein length (aa)	Calculated Molecular Weight (kDa)	Function
YP_007641377.1	N	Nucleoprotein	66-1334	1,269	422	47.8	Encapsidates genomic RNA, forms nucleocapsidn, essential for replication/transcription
YP_007641378.1	P	Phosphoprotein	1363-2244	882	293	32.4	Polymerase cofactor, regulates transcription/replication, involved in immune evasion
YP_007641379.1	M	Matrix protein	2315-3004	690	229	26.2	Facilitates virus assembly and budding; links nucleoprotein to envelope
YP_007641380.1	G	Glycoprotein	3053-4645	1,593	530	58.9	Surface spike protein; mediates host cell attachment and entry
YP_007641381.1	L	Large protein	4763-11041	6,279	2,092	238.4	RNA-dependent RNA polymerase; conducts replication and transcription

CHPV was first identified in 1965 from febrile patients in Chandipura village, Nagpur district, Maharashtra, India [7]. For several decades it remained relatively overlooked, with only sporadic detections from humans and sandflies. Its clinical relevance became apparent in the early 2000s when outbreaks of acute encephalitis with high fatality rates were reported in Indian pediatric populations. Major epidemics were documented in Andhra Pradesh (2003), Gujarat (2004), and Maharashtra (2007), with case fatality rates often ranging between 55% and 75% [8,9]. The disease predominantly affects children under 15 years of age and is characterized by acute fever, vomiting, altered consciousness, seizures, and rapid neurological decline [10–12]. In many regions, CHPV-associated cases are subsumed under the broader category of Acute Encephalitis Syndrome (AES), contributing to diagnostic underrecognition. Although sporadic outbreaks have been reported in recent years, the absence of active surveillance and limited molecular testing raise concern about silent circulation. Given the rapid disease progression, lack of antivirals or vaccines, and unclear transmission reservoirs, CHPV remains an under-recognized but clinically significant neurotropic virus.

Phlebotomine sandflies are strongly implicated in CHPV transmission, but a single, definitive vector species has not been established [13]. Current evidence strongly implicates sandflies, particularly species of the genus *Sergentomyia* as the potential primary vectors, based on molecular detection of viral RNA, isolation of live virus from field-caught specimens, and ecological studies in endemic regions of India [14]. Early investigations identified *Phlebotomus papatasi* as a likely vector, demonstrating its competence through experimental infection and transmission to suckling mice. However, subsequent entomological surveys documented a decline in *Phlebotomus* populations, with *Sergentomyia* spp. predominating in outbreak zones [15,16]. Viral detection in both male and female sandflies indicates potential vertical transmission, suggesting that CHPV may be maintained within vector populations independent of vertebrate hosts [16]. CHPV RNA has been detected in field-collected sandflies using RT-PCR, although the frequency of detection varies across sampling sites and studies [17]. Seasonal and environmental factors further influence transmission, as human outbreaks often coincide with pre-monsoon and monsoon months when temperature, humidity, and sandfly density peak [18]. Comparative consideration with other sandfly-borne pathogens, including *Leishmania* spp. and sandfly fever viruses, underscores the epidemiological importance of these vectors [19]. Despite extensive evidence, key aspects of CHPV transmission, including vector competence under natural conditions and the efficiency of horizontal and vertical transmission, remain incompletely understood.

CHPV exhibits a geographically restricted but ecologically significant distribution, with human infections reported exclusively in India and vector isolations documented in Africa [20]. In India, outbreaks have been reported across Andhra Pradesh, Gujarat, Maharashtra, and Odisha, primarily affecting pediatric populations [12]. Epidemiological observations suggest circulation in discrete endemic pockets, emerging episodically during pre-monsoon and monsoon seasons, likely influenced by vector abundance and environmental conditions. Beyond India, CHPV RNA and live virus have been detected in sandflies from Senegal, Nigeria, and Kenya [20], highlighting a broader ecological range than human case reports alone suggest. This disparity indicates possible underdiagnosis, silent circulation, or host-restriction mechanisms that limit symptomatic disease outside India. Understanding these spatial dynamics is critical for anticipating the emergence of outbreaks and linking genomic variability to epidemiological patterns.

Diagnosis relies primarily on molecular techniques, including conventional and real-time RT-PCR, which can detect low viral loads in blood, cerebrospinal fluid, and sandflies. Serological assays provide supplementary value but are constrained by transient antibody responses and potential cross-reactivity [21]. No licensed vaccines or antiviral therapies exist, and supportive care remains the mainstay of management. The rapid progression and narrow symptomatic window underscore the public health urgency of early detection and outbreak containment, emphasizing the need for integrated molecular diagnostics, surveillance, and vector control strategies.

Despite the availability of a limited number of complete CHPV genomes, an integrative evaluation combining phylogeographic structure, host-associated evolutionary pressures, and antigenic mapping remains limited. In particular, whether evolutionary constraints differ between human- and vector-derived sequences remains insufficiently characterized. Furthermore, it remains unclear whether sites under adaptive evolution in structural proteins, particularly the glycoprotein, are associated with predicted B-cell epitope regions suggestive of immune-mediated selection.

To address these questions, we tested three primary hypotheses: **(i) CHPV isolates exhibit phylogenetic structuring associated with their geographic and host origins**; **(ii) evolutionary pressures differ between human- and vector-derived isolates, resulting in measurable differences in genetic divergence and selection intensity**; and **(iii) sites under diversifying selection in structural proteins overlap with predicted B-cell epitope regions.**

## Methodology

### Epidemiological and vector distribution mapping

Data on confirmed human cases of CHPV and reports of virus isolation from sandflies were compiled from peer-reviewed literature, national surveillance reports, and regional outbreak investigations. To ensure comprehensive data capture, a structured literature search was conducted using PubMed and Google Scholar, combining the terms “Chandipura virus,” “CHPV,” “human cases,” “outbreak,” “sandfly,” and “virus isolation.” Additional information was obtained from national surveillance summaries and publicly accessible outbreak reports. The literature search included reports available up to October 2025, and inclusion criteria were laboratory-confirmed cases with district-level geographic information. In instances where peer-reviewed documentation was unavailable, credible media reports citing official health authority data were included to supplement surveillance information. Extracted data, including state, district/region, reporting period, case counts, and source, are summarized in S3 Table. Cases were mapped to the district level, providing an aggregated and policy-relevant spatial resolution that aligns with administrative boundaries used for public health planning and surveillance.

Available distribution data for phlebotomine sandfly species, including the genera *Sergentomyia, Phlebotomus, and Grassomyia*, were compiled from published entomological studies [22,23] and overlaid to contextualize potential virus-vector associations across districts, as reliable occurrence data were available for these genera across the Indian subcontinent.

Spatial mapping and visualization were performed in QGIS (version 3.44) [24], using district boundary shapefiles to generate georeferenced maps. Layers representing human CHPV cases, sandfly species distributions, and confirmed vector isolations were integrated to provide a comprehensive overview of the geographic overlap between CHPV occurrence and vector presence. This district-level approach enables identification of potential transmission hotspots, facilitates epidemiological interpretation of outbreak patterns, and supports targeted vector surveillance and intervention strategies.

### Sequence data retrieval and phylogenetic analysis

All available complete CHPV genomes (n = 23) were retrieved from the NCBI GenBank database as of October 2025. These genomes encompassed isolates derived from human (n = 5), sandfly (n = 17), and hedgehog (n = 1), thereby representing the full set of publicly accessible complete CHPV sequences at the time of analysis. The metadata of all CHPV isolates included in this study is summarized in Table 2. To contextualize CHPV within the Rhabdoviridae family, complete reference genomes of closely related vesiculoviruses were included: Vesicular stomatitis virus Indiana (VSVI), Vesicular stomatitis virus New Jersey (VSVNJ), Isfahan virus (ISFV), and Piry virus (PV). Their accession IDs and genome size have been summarized in S2 Table. These viruses were selected based on their genetic and ecological relevance to CHPV, providing a phylogenetically meaningful framework for assessing both genus- and family-level evolutionary relationships. Additionally, Rabies virus (RABV) (genus *Lyssavirus*) was incorporated as an outgroup to improve tree rooting and robustness.

**Table 2:** Metadata of 23 Chandipura virus whole-genome sequences included in phylogenetic analysis.

GenBank Accession	Size (bp)	Sequencing Technology	Molecule Type	Strain/Isolate	Host	Geolocation	Collection Year
ON158116.1	11109	Illumina	viral cRNA	BAR_SS10_763	Phlebotomine sandflies	Kenya: Baringo	2016
ON158117.1	11109	Illumina	viral cRNA	BAR_SS12_700	Phlebotomine sandflies	Kenya: Baringo	2017
HM627187.1	11067	–	viral cRNA	ArD111125	sandfly	Senegal	1978
MT019609.1	11061	llumina	viral cRNA	ArD89384	Phlebotomine sandflies	Senegal: Barkedji	1992
MT019610.1	11061	llumina	viral cRNA	ArD105134	Phlebotomine sandflies	Senegal: Barkedji	1994
MT019618.1	11077	llumina	viral cRNA	ArD111313	Phlebotomine sandflies	Senegal: Barkedji, West Africa	1995
MT019617.1	11077	Illumina	viral cRNA	ArD111213	Phlebotomine sandflies	Senegal: Barkedji	1995
MT019619.1	11077	Illumina	viral cRNA	ArD129179	Phlebotomine sandflies	Senegal: Barkedji, West Africa	1997
MT019616.1	11061	llumina	viral cRNA	ArD129178	Phlebotomine sandflies	Senegal: Barkedji	1997
MT019614.1	11061	llumina	viral cRNA	ArD129212	Phlebotomine sandflies	Senegal: Barkedji	1997
MT019613.1	11061	llumina	viral cRNA	ArD129199	Phlebotomine sandflies	Senegal: Barkedji	1997
MT019612	11061	llumina	viral cRNA	ArD129185	Phlebotomine sandflies	Senegal: Barkedji	1997
MT019611.1	11061	llumina	viral cRNA	ArD111758	Phlebotomine sandflies	Senegal: Kedougou	1995
MT019615.1	11061	llumina	viral cRNA	ArD111757	Phlebotomine sandflies	Senegal: Kedougou	1995
MT019608.1	11061	llumina	viral cRNA	ArD111761	Phlebotomine sandflies	Senegal: Kedougou	1995
ON158118.1	11109	Illumina	viral cRNA	TUR_SS1_361	Phlebotomine sandflies	Kenya: Turkana	2016
ON158119.1	11117	Illumina	viral cRNA	TUR_SS5_851	Phlebotomine sandflies	Kenya: Turkana	2017
HM627186.1	11061	–	viral cRNA	IB An 9978	Hedgehog	Nigeria	1966
GU212858.1	11120	–	viral cRNA	CIN 0327	*Homo sapiens*	India: Karimnagar, Andhra Pradesh	2003
GU212856.1	11120	–	viral cRNA	CIN 0451	*Homo sapiens*	India: Vadodara, Gujarat	2004
GU190711.1	11094	–	viral cRNA	CIN 0728	*Homo sapiens*	India: Nagpur, Maharashtra	2007
GU212857.1	11083	–	viral cRNA	CIN 0755	*Homo sapiens*	India: Warangal, Andhra Pradesh	2007
PQ185534.2	11078	Illumina	viral cRNA	CHPV0744	*Homo sapiens*	India: Gujarat, Patan	2024

All sequences were aligned using the Multiple Sequence Comparison by Log-Expectation (MUSCLE) algorithm implemented in MEGA12 [25]. Whole-genome phylogenetic analysis was conducted using only the coding sequences of each genome. Maximum likelihood phylogenetic analyses were performed using IQ-TREE (version 2.2.0) [26] for the whole-genome coding sequences and each of the five individual viral protein genes: glycoprotein, large protein (polymerase), matrix protein, nucleoprotein, and phosphoprotein. The optimal nucleotide substitution models were determined using ModelFinder implemented in IQ-TREE based on the Bayesian information criterion (BIC). The selected models were as follows: GTR + F + I + R3 for the whole-genome and glycoprotein datasets, GTR + F + R4 for the large protein, TIM2 + F + G4 for the matrix protein, GTR + F + G4 for the nucleoprotein, and K3Pu+F + I + R2 for the phosphoprotein. All trees were reconstructed with 1,000 ultrafast bootstrap replicates to assess nodal support. Visualization and annotation were performed using the interactive Tree of Life (iTOL) platform [27]. Tip labels were annotated with host species (Human [H], Sandfly [S], Hedgehog [HG]) and geographic origin (country and regional abbreviations), while bootstrap values were displayed at internal nodes.

### Assessment of temporal signal

To evaluate the temporal structure of the dataset and assess its suitability for molecular clock-based analyses, root-to-tip regression analyses were performed with TempEst [28]. Maximum likelihood phylogenetic trees inferred for the complete genome and individual gene datasets (N, P, M, G, and L) were used as input. For each dataset, the best-fitting root was determined within TempEst to optimize the correlation between sampling time and root-to-tip genetic divergence. Linear regression analyses were conducted by plotting root-to-tip genetic distances against sampling dates. The strength of the temporal signal was evaluated using the coefficient of determination (R²) and correlation coefficient (r).

### Bayesian coalescent-based demographic analysis

Bayesian coalescent-based demographic analyses were performed using BEAST 2.7.7 [29] to infer temporal changes in effective population size of CHPV. Analyses were conducted on the complete genome dataset and for each of the five protein-coding genes (N, P, M, G, and L). Time-calibrated phylogenies were reconstructed using both a strict molecular clock and an uncorrelated lognormal relaxed clock to account for potential rate variation among lineages. Given the relatively small dataset, the strict clock provides a reasonable approximation, while the relaxed clock captures possible heterogeneity in evolutionary rates. The optimal nucleotide substitution models for each dataset were selected using ModelFinder implemented in IQ-TREE. Markov Chain Monte Carlo (MCMC) analyses were run for 20 million generations under the strict clock model and 50 million generations under the relaxed clock model, with parameters sampled every 5,000 steps. Convergence and adequate sampling were assessed using effective sample size (ESS > 200) in Tracer 1.7.2, and the initial 10% of samples were discarded as burn-in. Bayesian skyline plots were generated in Tracer to estimate temporal changes in scaled effective population size (Neτ), where Ne represents effective population size and τ denotes the coalescent time scaling factor. Median estimates and their corresponding 95% highest posterior density (HPD) intervals were used to interpret demographic trends. Due to the limited sample size (n = 23) and potential constraints in temporal signal, the resulting demographic inferences were interpreted cautiously and considered exploratory.

### Pairwise evolutionary distance estimates

To quantify sequence divergence among CHPV isolates and selected reference rhabdoviruses, pairwise evolutionary distances were estimated using the Kimura 2-parameter (K2P) model of MEGA12, applying pairwise deletion for sites containing gaps [25]. A total of 28 nucleotide sequences were included, comprising complete genomes of human- and sandfly-derived CHPV isolates, one hedgehog-derived isolate, and representative reference rhabdoviruses: VSVI and VSVNJ, ISFV, PV, and RABV. Ambiguous nucleotide positions were handled using pairwise deletion, resulting in a final alignment of 13,606 nucleotide sites. All calculations were performed in MEGA12 [25], generating a matrix of pairwise K2P evolutionary distances expressed as substitutions per site. The resulting divergence matrix was visualized as a heat map using the Morpheus online tool [30], providing a clear comparative overview of genetic distances within CHPV and between CHPV and other rhabdoviruses.

In addition to whole-genome comparisons, gene-wise nucleotide divergence was assessed for the five major CHPV genes, glycoprotein, matrix, nucleoprotein, large protein, and phosphoprotein, across all 23 CHPV isolates. Pairwise evolutionary distances for each gene were calculated using the Kimura 2-parameter model in MEGA12 under the same pairwise deletion settings, and individual distance matrices were generated for each protein-coding region. The resulting nucleotide evolutionary distances were expressed as substitutions per site.

To complement the nucleotide-level analyses, amino acid sequence divergence was quantified for the same five CHPV proteins. For each protein, pairwise amino acid distances were calculated using the p-distance model in MEGA12, which expresses the proportion of amino acid differences per site between sequences. amino acid p-distances, these values were expressed as percentages of amino acid differences. These analyses provided protein-specific estimates of intra- and inter-lineage divergence among CHPV isolates.

### Selection pressure analysis, epitope prediction, and structural mapping of CHPV proteins

To evaluate the evolutionary pressures acting on CHPV coding sequences, gene-wise selection analyses were performed on the nucleotide alignments of the three structural proteins: glycoprotein, matrix, and nucleoprotein. These proteins were specifically chosen because they are directly involved in virion formation and host interactions, making them likely targets of host-driven adaptive pressures. The phosphoprotein and large polymerase protein were excluded from episodic diversification analysis, as these proteins primarily function in viral replication as polymerase cofactors and as the RNA-dependent RNA polymerase, and are largely internal replication-associated proteins, for which adaptive variation is less likely to reflect host-driven selective pressures.

To quantify overall selection pressure, the ratio of nonsynonymous to synonymous substitutions (dN/dS) was estimated using the Single-Likelihood Ancestor Counting (SLAC) method implemented in the HyPhy package via the Datamonkey web server [31]. Analyses were conducted on codon-aligned nucleotide sequences using a maximum likelihood phylogenetic tree inferred under the best-fit substitution model. Mean dN/dS ratios were calculated for each gene to assess the extent of purifying selection and to enable comparative evaluation of evolutionary constraints across host-associated groups.

Codon-level selection was further assessed using the Mixed Effects Model of Evolution (MEME) implemented in the HyPhy package through the Datamonkey web server [31]. MEME identifies sites evolving under episodic diversifying selection, with p-values < 0.05 considered statistically significant. To integrate structural and immunological context, B-cell linear epitopes for each structural protein were predicted using IEDB BepiPred version 2.0 with a default threshold of 0.5 [32].

Structural prediction of the CHPV glycoprotein (ADO63658.1), matrix protein (ADO63657.1), and nucleoprotein (ADO63655.1) was performed using AlphaFold3 [33] with input sequences extracted from the CHPV genome (GU212856.1). AlphaFold3 outputs included predicted 3D structures along with accompanying model-confidence metrics. The predicted template modeling score (pTM) was used to assess confidence in the overall protein fold, whereas per-residue pLDDT scores were used to assess local structural confidence. pTM values were interpreted as indicators of confidence in the predicted global fold.pLDDT) pTM scores approaching 1.0 (with values >0.5 indicating reliable global fold) and per-residue pLDDT scores >70 (and >90 indicating very high confidence) were used as criteria to assess structural reliability. These parameters were used to evaluate overall fold reliability; they were taken directly from the model output.

PyMOL [34] was used to map significant diversifying selection sites and predicted B-cell epitopes onto the AlphaFold-predicted protein structures, assessing their spatial relationship to predicted B cell regions and providing insights into potential effects on immune recognition and viral evolution.

## Results

### Geographical distribution of CHPV cases and vector associations

**Fig 2:**
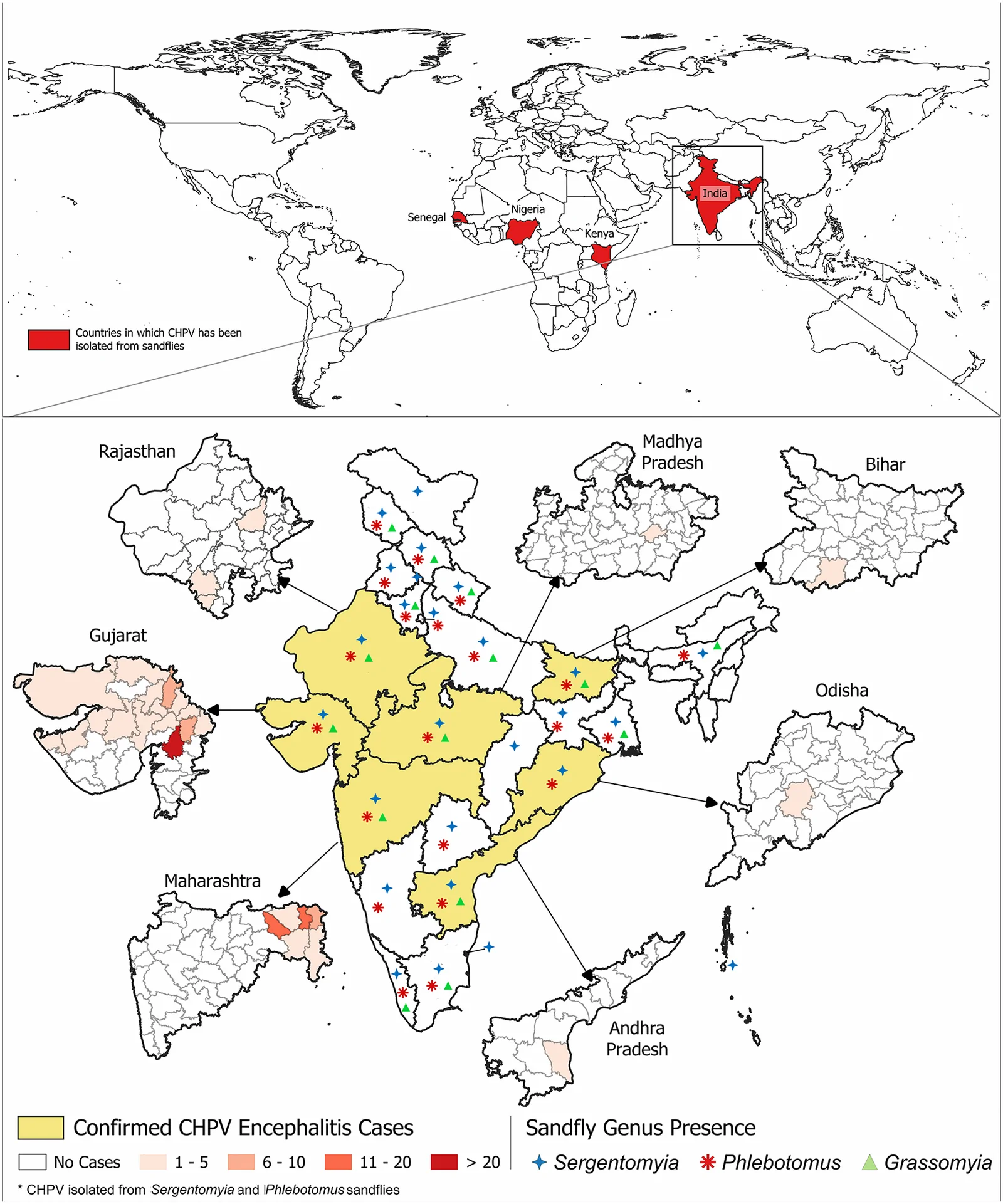
Global distribution of Chandipura virus (CHPV) human cases, vector isolations, and sandfly species occurrence. The map illustrates confirmed human CHPV outbreaks across Indian states (with case numbers indicated by proportional symbols), regions in India and Africa where CHPV has been isolated from sandfly species (*Sergentomyia* spp. and *Phlebotomus* spp.), and the documented distribution of sandfly species in India. Data were compiled from published outbreak reports and entomological surveys. Base administrative boundaries (shapefile) obtained from Survey of India (Government of India) (https://onlinemaps.surveyofindia.gov.in/Digital_Product_Show.aspx); used with reproduction rights as per SoI copyright policy. https://surveyofindia.gov.in/pages/copyright-policy.

To understand how CHPV persists and spreads, we mapped its geographic distribution relative to potential vectors. The spatial distribution of CHPV cases and vector isolations is shown in Fig 2. In India, confirmed human cases have been reported from multiple states (S3 Table). The largest clusters were documented in Andhra Pradesh (Warangal, 46 cases; Karimnagar, 17 cases; Nizamabad, 5 cases; Medak, 2 cases; Nalgonda, 2 cases; Mehboobnagar, 2 cases; Nellore, 2 cases; Khammam, 1 case; Adilabad, 1 case), totalling 78 cases. In Maharashtra, outbreaks occurred in Bhandara (12 cases), Wardha (11 cases), Nagpur (6 cases), Gondia (6 cases), Gadchiroli (4 cases), and Chandrapur (2 cases), totalling 41 cases. Gujarat reported the widest distribution, with confirmed cases in Vadodara (22 cases), Sabarkantha (6 cases), Panchmahal (6 cases), Mehsana (4 cases), Aravalli (3 cases), Kheda (3 cases), Ahmedabad city (3 cases), Dahod (2 cases), and single cases from Mahisagar, Surendranagar, Gandhinagar, Jamnagar, Morbi, Banaskantha, Devbhumi Dwarka, and Kutch. In total, Gujarat has reported 61 cases; however, only 39 could be attributed to specific districts, with 22 cases remaining unattributed to a particular location. Smaller case clusters have also been reported from Madhya Pradesh (Jabalpur, 4 cases), Odisha (Gudrigao village, Daringbadi block, 4 cases), Rajasthan (Udaipur, 1 case; Dungarpur, 1 case; Shahpura, 1 case), and Bihar (Singhapar village, 1 case). These counts represent cases identifiable from the compiled sources and should not be interpreted as comprehensive estimates of cumulative national incidence. Vector isolation studies suggest sandflies play a role in CHPV ecology. In India, the first isolation of CHPV was from *Phlebotomus* spp. sandflies collected in Aurangabad district, Maharashtra, during the late 1960s [35], implicating *Phlebotomus* as a primary vector before later isolations from *Sergentomyia* spp. Subsequently, CHPV RNA was detected in *Sergentomyia* spp. collected in Karimnagar district, then part of Andhra Pradesh, during the 2003 encephalitis outbreak [17].CHPV was later isolated from *Sergentomyia* spp. collected in Nagpur district, Maharashtra [14]. In Africa, CHPV was detected in *Sergentomyia* spp. in Senegal, and in *Phlebotomus* spp. in Nigeria and Kenya [36]. The map also shows the distribution of three sandfly genera in India: Sergentomyia, Phlebotomus, and Grassomyia.

### Whole-genome and gene-specific phylogenetic analyses

Phylogenetic reconstruction using maximum likelihood methods in IQ-TREE with 1,000 ultrafast bootstrap replicates was performed on 23 CHPV genomes from India and Africa (Senegal, Nigeria, and Kenya) along with five representative rhabdoviruses. The analyzed sequences spanned from 1966 to 2024, encompassing both historical and contemporary isolates. The optimal substitution models selected by ModelFinder varied across datasets: GTR + F + I + R3 for the whole-genome and glycoprotein datasets, GTR + F + R4 for the large protein, TIM2 + F + G4 for the matrix protein, GTR + F + G4 for the nucleoprotein, and K3Pu+F + I + R2 for the phosphoprotein.

**Fig 3:**
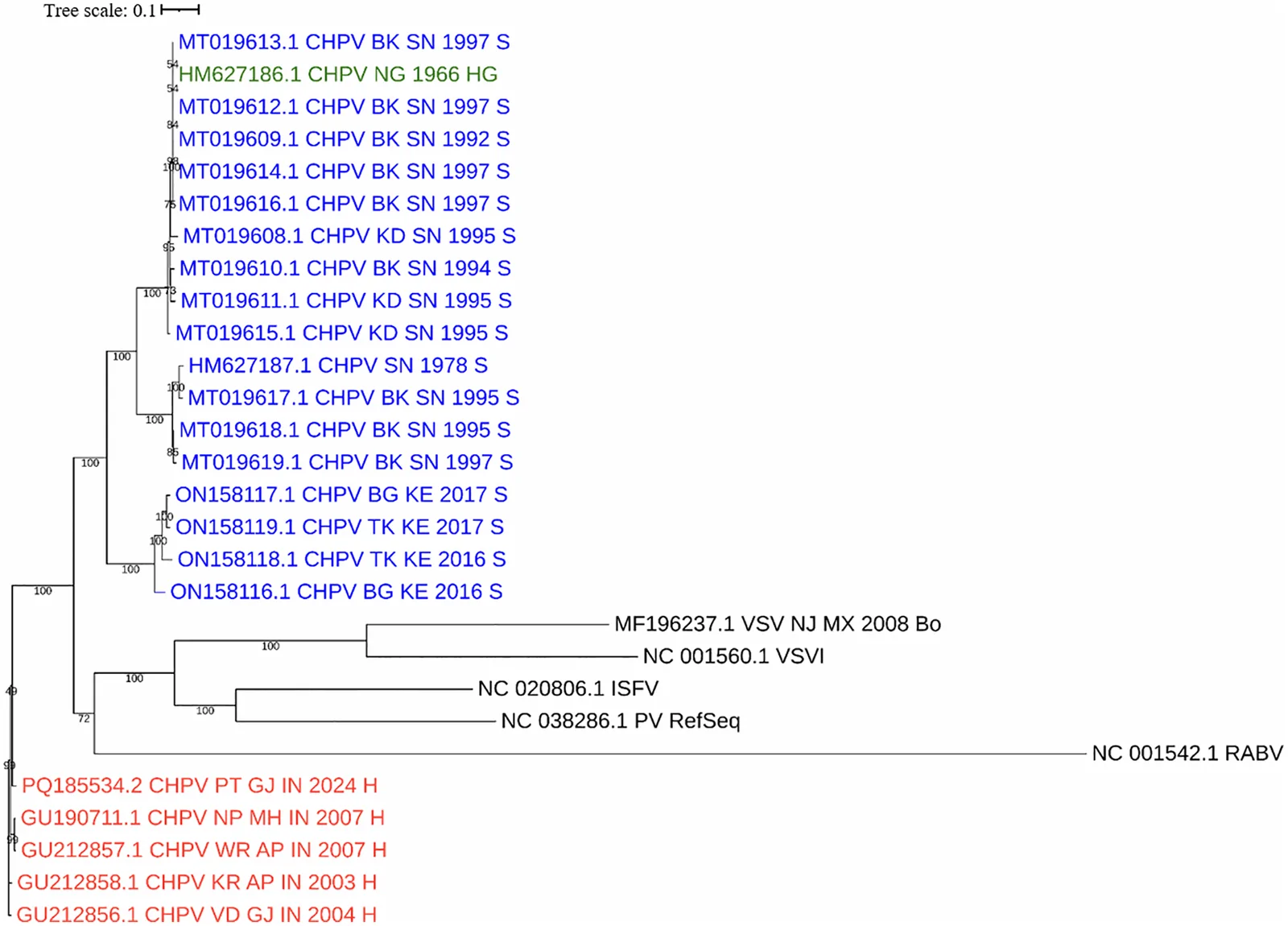
Maximum-likelihood phylogenetic tree based on whole-genome nucleotide sequences of CHPV and other rhabdoviruses. The tree was inferred using IQ-TREE 2.2.0 under the best-fit nucleotide substitution model selected by ModelFinder. Bootstrap support values (1,000 replicates) are shown at the nodes. Included in the analysis are Vesicular stomatitis virus Indiana (VSVI), Vesicular stomatitis virus New Jersey (VSVNJ, bovine (Bo)), Isfahan virus (ISFV), Piry virus (PV), and Rabies virus (RABV) as reference rhabdoviruses. Tip labels indicate host species (Human [H], Sandfly [S], Hedgehog [HG]) and country of origin (e.g., India [IN], Kenya [KE], Senegal [SN], Nigeria [NG], Mexico [MX]), along with regional abbreviations (e.g., Gujarat [GJ], Vadodara [VD], Warangal [WR], Nagpur [NP], Karimnagar [KN], Andhra Pradesh [AP], Maharashtra [MH], Patan [PT], Turkana [TK], Baringo [BG], Kedougou [KD], Barkedji [BK]).

The whole-genome tree revealed geographic and taxonomic separation, as illustrated in Fig 3. CHPV sequences from India formed a clade with strong bootstrap support (100%), distinct from those from Africa. Within the African lineage, isolates from Senegal, Kenya, and Nigeria clustered together but remained separate from the Indian clade. The Nigerian hedgehog isolate from 1966 clustered with Senegalese sandfly-derived CHPV strains. Further resolution within the African lineage reveal subclades corresponding to Kenyan and Senegalese isolates (bootstrap = 96% and 99%, respectively). The most recent Indian isolate from 2024 clustered within the same clade as earlier Indian strains from 2003-2007. When CHPV was analyzed alongside five related rhabdoviruses, including ISFV, PV, VSVI, VSVNJ, and RABV, CHPV clustered closest to ISFV (bootstrap = 100%), followed by PV and vesiculoviruses such as VSV. RABV formed the most distant branch.

**Fig 4:**
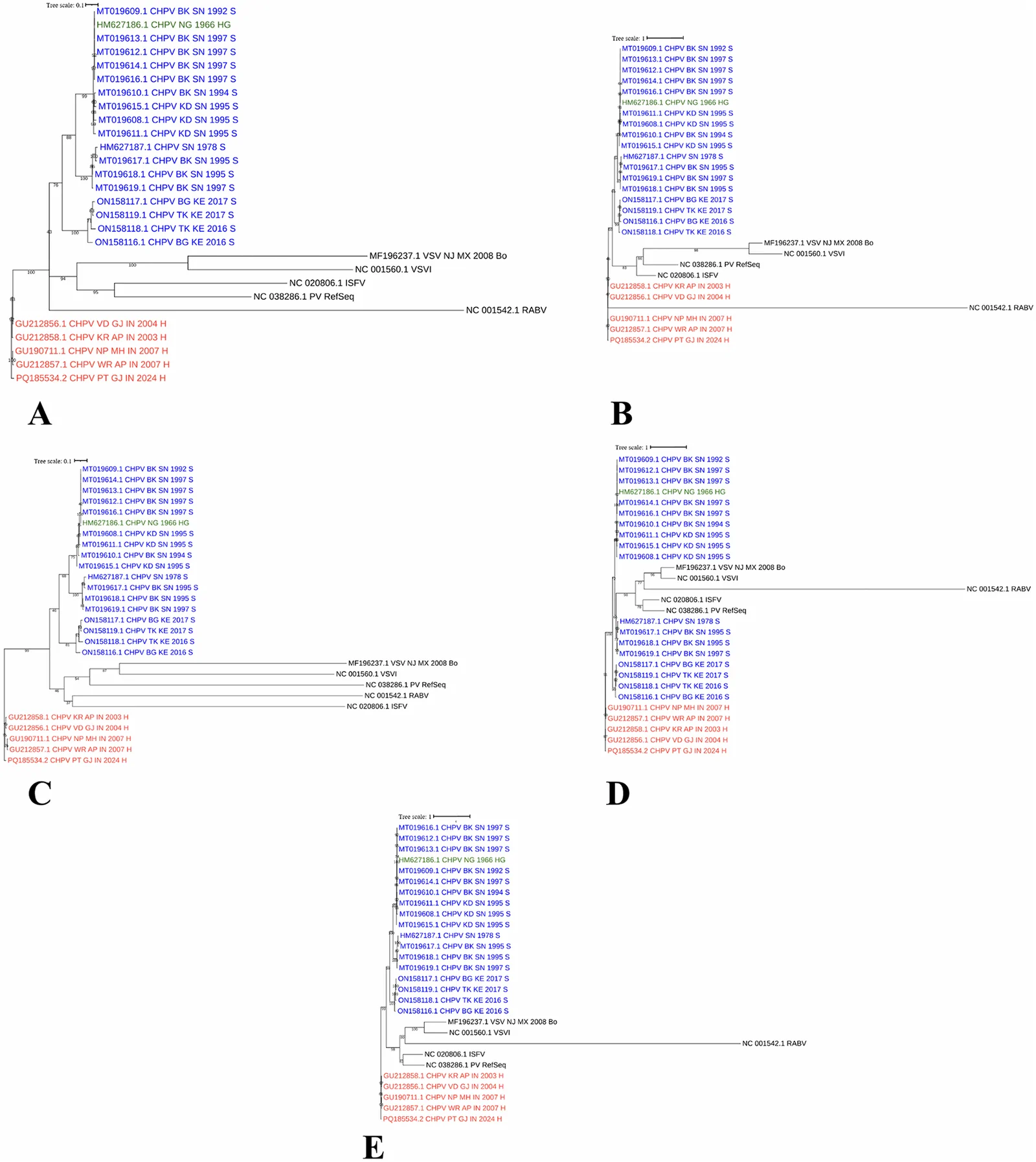
Maximum-likelihood phylogenetic trees of CHPV coding sequences for individual genes. Five phylogenetic trees were constructed from the coding sequences of the glycoprotein, matrix protein, nucleoprotein, phosphoprotein, and large RNA-dependent RNA polymerase labeled A to E. The trees were inferred using IQ-TREE 2.2.0 under the best-fit nucleotide substitution model selected by ModelFinder with bootstrap values from 1,000 replicates indicated at the nodes. The analysis includes CHPV sequences from India and African countries, along with representative rhabdoviruses: Vesicular stomatitis virus Indiana (VSVI), Vesicular stomatitis virus New Jersey (VSVNJ, bovine [Bo]), Isfahan virus (ISFV), Piry virus (PV), and Rabies virus (RABV). Tip labels show host species (Human [H], Sandfly [S], Hedgehog [HG]) and country of origin (India [IN], Kenya [KE], Senegal [SN], Nigeria [NG], Mexico [MX]) with regional abbreviations (Gujarat [GJ], Vadodara [VD], Warangal [WR], Nagpur [NP], Karimnagar [KN], Andhra Pradesh [AP], Maharashtra [MH], Patan [PT], Turkana [TK], Baringo [BG], Kedougou [KD], Barkedji [BK]).

Gene-wise phylogenetic trees (Fig 4) broadly mirrored the whole-genome clustering pattern, with CHPV genes clustering closest to ISFV across most loci. However, variable resolution was observed across genomic regions. The glycoprotein (G) gene tree maintained the separation of Indian and African clades with strong bootstrap support (100%). However, it showed reduced resolution within the African lineage, failing to distinguish Kenyan and Senegalese isolates as separate subclades clearly. In contrast, the large protein, matrix protein, nucleoprotein, and phosphoprotein gene trees resolved these subclades with strong bootstrap support, consistent with the whole-genome topology. In addition, the nucleoprotein, matrix, polymerase, and phosphoprotein genes showed similar clustering patterns relative to the reference rhabdoviruses.

### Temporal signal assessment and Bayesian skyline analysis of CHPV population dynamics

Root-to-tip regression analyses were performed to assess the temporal signal across the CHPV whole genome and individual gene datasets using TempEst. The whole-genome dataset demonstrated a moderate temporal signal (R² = 0.441; r = 0.6641), indicating a positive correlation between sampling time and genetic divergence (S1A Fig). Gene-wise analyses revealed similar patterns, with R² values ranging from 0.3519 to 0.5082 (S1B–S1F Fig). Among the genes, the matrix protein exhibited the strongest temporal signal (R² = 0.5082; r = 0.7129), whereas the phosphoprotein showed comparatively weaker signal (R² = 0.3519; r = 0.5932). The glycoprotein (R² = 0.4366), nucleoprotein (R² = 0.4127), and large protein (R² = 0.4537) displayed moderate levels of clock-like behavior.

**Fig 5:**
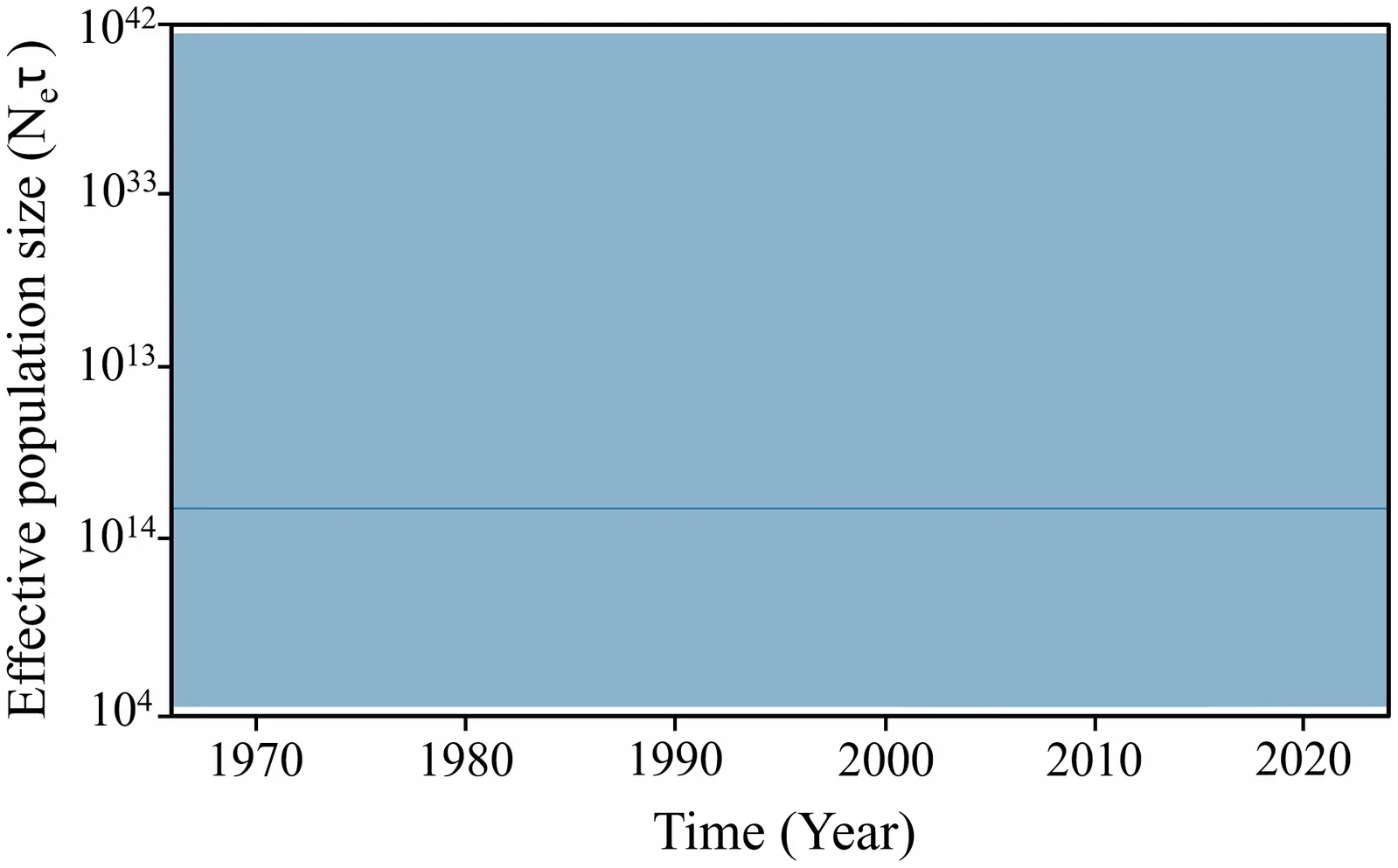
Bayesian skyline plot of CHPV inferred from the whole genome dataset. Bayesian skyline reconstruction of effective population size (N_e_τ) for CHPV based on the complete genome dataset (n = 23), inferred under an uncorrelated lognormal relaxed molecular clock model using BEAST 2.7.7. The solid line represents the median estimate, and the shaded region indicates the 95% highest posterior density (HPD) interval. The x-axis denotes time (years), and the y-axis represents scaled effective population size (N_e_τ).

Bayesian skyline analysis of the CHPV whole genome dataset performed under uncorrelated lognormal uncorrelated lognormal relaxed-clock model revealed a largely constant effective population size (N_e_τ) over time, with no clear evidence of demographic expansion or decline (Fig 5). The 95% highest posterior density (HPD) intervals were broad across the entire time range, indicating substantial uncertainty in the estimates. Analyses performed under a strict molecular clock model produced results comparable to those under a relaxed molecular clock model, with similarly flat skyline trajectories (S2A Fig). Gene-wise skyline analyses of the nucleoprotein, phosphoprotein, matrix, glycoprotein, and large protein-coding regions based on uncorrelated lognormal relaxed clock and strict molecular clock also showed consistent patterns of temporal stability with minor, non-systematic fluctuations, none of which were supported by their corresponding HPD intervals (S2B–S2F and S3A–S3F Figs).

### Pairwise evolutionary distance estimates

**Fig 6:**
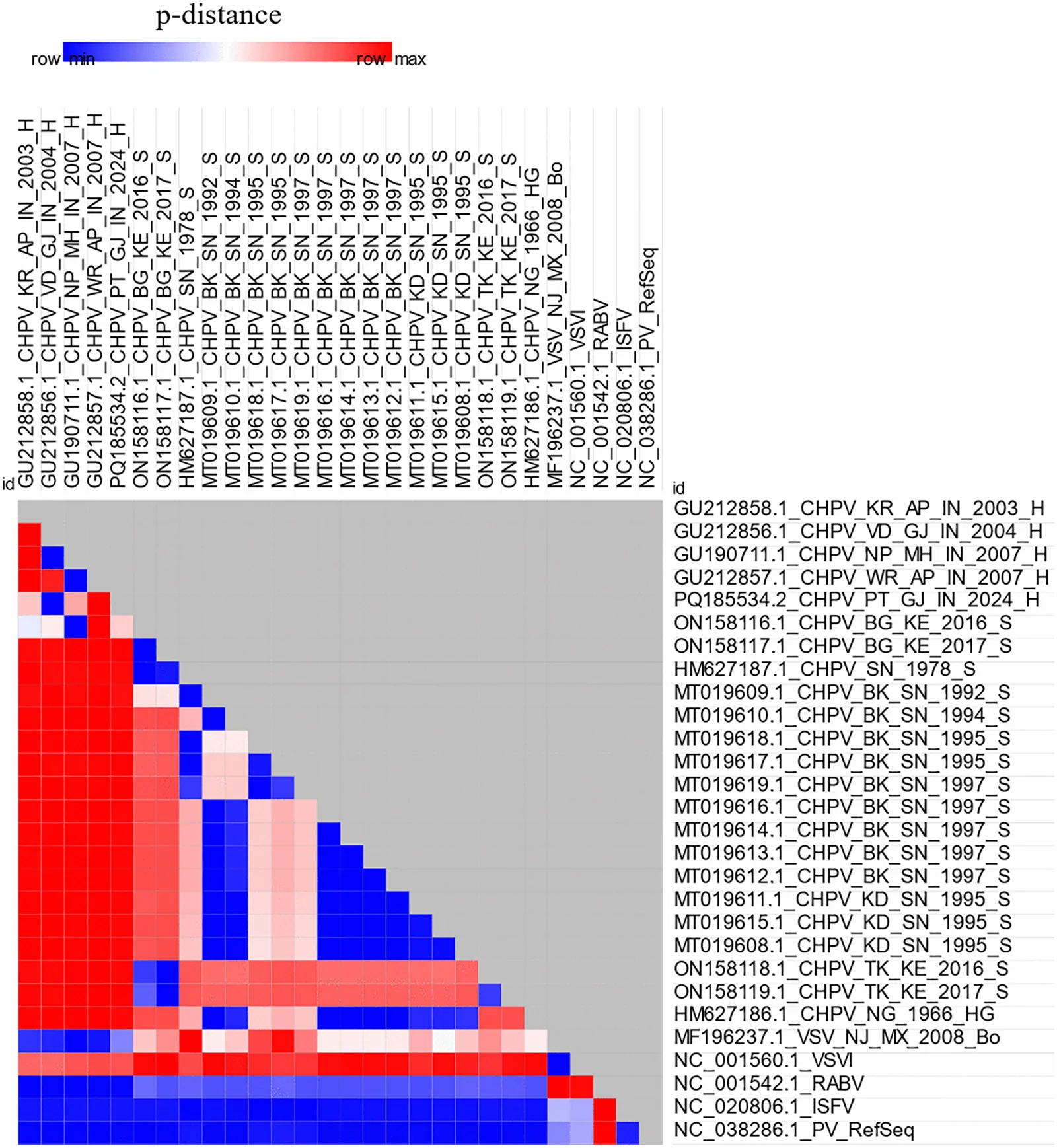
Heatmap representing pairwise nucleotide distances among 23 Chandipura virus (CHPV) isolates and five reference rhabdoviruses, based on full genome sequences. Included reference viruses are Vesicular stomatitis virus Indiana (VSVI), Vesicular stomatitis virus New Jersey (VSVNJ, bovine), Isfahan virus (ISFV), Piry virus (PV), and Rabies virus (RABV).

Whole-genome pairwise evolutionary distances among CHPV isolates with related rhabdoviruses were estimated using the Kimura 2-parameter model to assess intra- and inter-clade diversity, evolutionary relationships, and phylogeographic structure. Pairwise evolutionary distance among the included sequences are illustrated as a heat map in Fig 6. The Indian CHPV clade (n = 5), consisting solely of human isolates collected between 2003 and 2024, exhibited low genetic divergence, with pairwise distances ranging from 0.0148 to 0.0302 and a mean of 0.0239 ± 0.0072.

Conversely, the African CHPV clade (n = 18), composed predominantly of sandfly-derived isolates from Kenya and Senegal, along with a hedgehog-derived isolate from Nigeria, demonstrated greater genetic heterogeneity. Intra-clade distances ranged widely from 0.0000% up to 0.2793, with a mean divergence of 0.0445 ± 0.0589. The near-zero distances observed within subsets of isolates reflect tight clustering, whereas the upper range values indicate deeper divergence among isolates from different times or locations.

Inter-clade comparisons between Indian and African CHPV isolates revealed markedly higher genetic divergence, with pairwise distances ranging narrowly between 0.2720 and 0.2851 and a mean of 0.2792 ± 0.0046.

Comparative analysis with other related rhabdoviruses, including VSVNJ, VSVI, RABV, ISFV, and PV showed extensive genetic divergence from CHPV. The inter-genus distances ranged from 0.5185 to 0.7975, with a mean of 0.6634 ± 0.0790. Among the non-CHPV rhabdoviruses, pairwise genetic distances ranged from 0.5120 to 1.022, with a mean of 0.773 ± 0.170.

Additionally, protein-specific nucleotide divergence analyses were performed for five key CHPV proteins (glycoprotein, matrix, nucleoprotein, polymerase, and phosphoprotein). Nucleotide divergence within Indian CHPV isolates was low across all five protein genes, ranging from approximately 0.0246 ± 0.0003 to 0.0300 ± 0.0180, indicating high sequence conservation. In contrast, African isolates exhibit substantially higher nucleotide diversity, with mean divergence values ranging from 0.180 ± 0.0600 to 0.2422 ± 0.0532. Inter-group nucleotide divergence between Indian and African isolates is markedly higher for all proteins, ranging from 0.2470 ± 0.013 to 0.3250 ± 0.0380 substitution per site.

Similarly, protein-specific amino acid divergence analyses were performed for the same five key CHPV proteins. Amino acid divergence within Indian CHPV isolates remains low across all proteins, ranging from approximately 0.16% ± 0.13% to 0.76% ± 0.54%, indicating strong sequence conservation. In contrast, African isolates show notably higher amino acid diversity, with mean divergence values ranging from 6.23% ± 1.97% to 14.11% ± 2.82%. Inter-group amino acid divergence between Indian and African isolates is similarly elevated for all proteins, ranging from 7.54% ± 0.68% to 15.94% ± 2.36%.

### Selection pressure analysis and mapping of predicted B-cell epitopes in CHPV glycoprotein, matrix, and nucleoprotein

Analysis of the complete dataset (n = 23) indicated that all five CHPV proteins are subjected predominantly to purifying selection. Despite purifying selection at the gene level, MEME identified evidence of episodic diversifying selection at specific codons. The estimated global dN/dS (ω) ratios were 0.0355 for the glycoprotein gene, 0.0287 for matrix protein, 0.0320 for N, 0.0812 for P, and 0.0311 for large protein. All ω values were substantially below 1, indicating pervasive evolutionary constraint across both structural and non-structural proteins. No evidence of gene-wide positive selection was observed. Among the five genes, the P gene exhibited the highest ω value, although it remained well below neutrality.

To evaluate differences in selective pressures between human- and sandfly-derived sequences, SLAC analyses were conducted separately for human-derived isolates (n = 5) and non-human derived isolates (n = 18), comprising 17 sandfly-derived isolates and one hedgehog-derived isolate. In both groups, all genes exhibited ω values below 1, confirming that purifying selection predominates irrespective of host origin. For the glycoprotein, matrix protein, and large protein genes, ω values were moderately higher in human-derived sequences (0.0567, 0.0584, and 0.0490, respectively) compared with sandfly-derived sequences (0.0343, 0.0230, and 0.0264), suggesting relatively reduced evolutionary constraint in human isolates for these proteins. In contrast, the nucleoprotein gene showed a lower ω value in human-derived sequences (0.0164) than in sandfly-derived sequences (0.0268), while the P gene displayed comparable ω values between human (0.0646) and sandfly (0.0801) isolates. Overall, the observed differences in ω values between human and sandfly isolates were modest and gene-dependent, and no consistent genome-wide pattern of differential selective pressure was detected. Host comparisons are inherently limited by the currently available complete CHPV genomic dataset (n = 23).

Further, a combined selection-pressure, epitope-prediction, and structural mapping analysis was performed for the CHPV glycoprotein, matrix protein, and nucleoprotein to identify adaptive signatures and their spatial relevance to predicted B-cell epitope regions. Structural modelling using AlphaFold3 provided reliable confidence metrics for all three proteins. The glycoprotein exhibited a pTM score of 0.52, indicating that the predicted model meets the minimum threshold for reliable fold similarity. The matrix protein showed a pTM score of 0.71, consistent with a moderately confident global fold. The nucleoprotein yielded a pTM score of 0.94, reflecting high structural reliability of the predicted model. Per-residue pLDDT confidence was inferred from the colour-coded AlphaFold output. Codon sites under episodic diversifying selection (MEME, p < 0.05) were mapped alongside BepiPred 2.0-predicted linear B-cell epitopes to evaluate their spatial relationship. The detailed results for each protein are described in the following subsections.

#### Glycoprotein.

**Fig 7:**
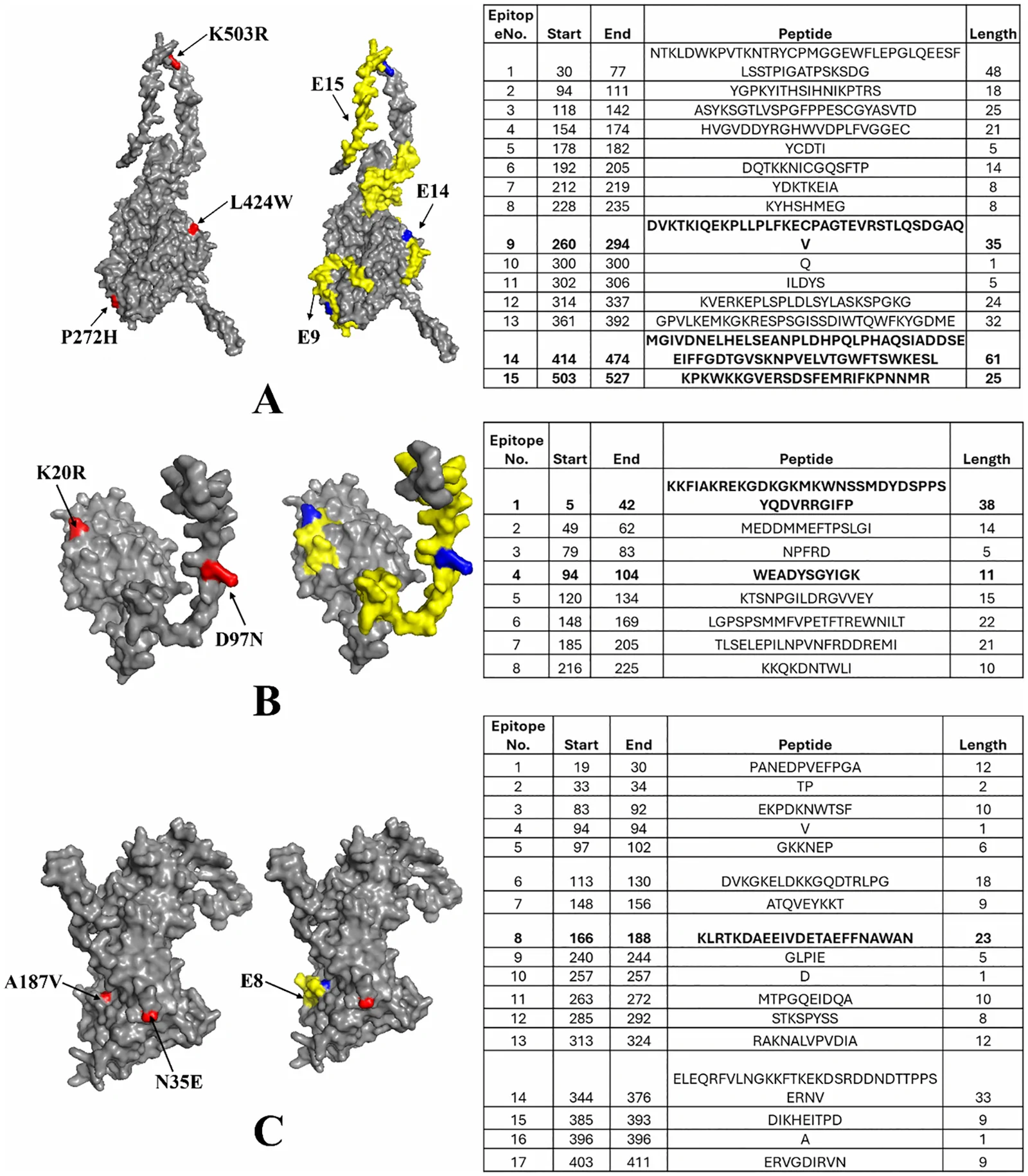
Structural mapping of episodic diversifying sites and predicted B-cell linear epitopes in CHPV structural proteins. Surface representations of (A) glycoprotein, (B) matrix protein, and (C) nucleoprotein are shown with residues under episodic diversifying selection (MEME) colored red, predicted B-cell linear epitopes (IEDB BepiPred) highlighted in yellow, and residues overlapping both categories indicated in blue. Each panel includes a table listing the predicted B-cell epitopes alongside the corresponding protein sequence positions, facilitating correlation between structural features and immunologically relevant sites.

Analysis of episodic diversifying selection on the CHPV glycoprotein identified several codon positions exhibiting amino acid changes with potential structural or functional implications have been summarized in S4 Table and demonstrated structurally in Fig 7A. MEME detected evidence of episodic positive selection at specific codons within the CHPV glycoprotein, with the complete site-by-site results are presented in S5 Table. Notably, P272H, L424W, and K503R showed variant prevalences in 1/23 sequences (4.35%), 1/23 sequences (4.35%), and 4/23 sequences (17.39%), respectively. All three glycoprotein sites (positions 272, 424, and 503) overlapped with predicted linear B-cell epitopes (epitopes E9, E14, and E15, respectively).

#### Matrix protein.

Episodic diversifying selection analysis of the CHPV matrix protein revealed two codon positions under variation: K20R in 8/23 sequences (34.78%) and D97N in 1/23 sequences (4.35%) (S4 Table and Fig 7B). MEME detected evidence of episodic positive selection at specific codons within the CHPV matrix protein, with the complete site-by-site results presented in S6 Table. Both matrix protein sites (positions 20 and 97) overlapped with predicted linear B-cell epitopes (epitopes E1 and E4, respectively).

#### Nucleoprotein.

Analysis of the CHPV nucleoprotein identified two codon positions under episodic diversifying selection: N35E in 5/23 sequences (21.74%) and A187V with A → V in 1/23 sequences (4.35%) (S5 Table (This should be S4 Table, not S5 Table) and Fig 7C). MEME detected evidence of episodic positive selection at specific codons within the CHPV nucleoprotein, with the complete site-by-site results presented in S7 Table. The substitution at position 35 is non-conservative, potentially altering local structure and protein interactions due to the change from a polar uncharged residue to a negatively charged residue. In contrast, the change at position 187 is conservative and is likely to have only minor effects on local packing. Among the 17 predicted linear B-cell epitopes, only one nucleoprotein site (position 187) overlapped with an epitope (epitope E8). Position 35 (N35E) did not overlap with any predicted B-cell epitope.

## Discussion

CHPV has long been recognized for its association with acute encephalitis outbreaks in India, yet its broader ecology, vector associations, and evolutionary dynamics remain incompletely understood. Mapping of human case distributions in this study reinforces earlier findings identifying Andhra Pradesh, Maharashtra, and Gujarat as endemic regions, with additional sporadic detections in Madhya Pradesh, Odisha, Rajasthan, and Bihar [37–40]. These areas overlap with the distribution of *Sergentomyia* and *Phlebotomus* sandflies, both of which have been detected carrying CHPV RNA in India and Africa. *Grassomyia* spp., though not directly linked to viral isolation, were also mapped to provide ecological context and potential leads for future entomological surveillance.

At present, evidence of sandfly involvement remains circumstantial but compelling. Both *Sergentomyia* and *Phlebotomus* species have been implicated as potential vectors through molecular detection of CHPV RNA in India and Africa. However, definitive confirmation is still lacking, as no experimental transmission studies have yet demonstrated vector competence. This leaves open the possibility that sandflies may act as incidental carriers rather than true biological vectors. Nevertheless, repeated virus detections from outbreak zones, coupled with human-vector habitat overlap, support the need for integrative ecological, entomological, and molecular approaches. Parallel genomic sequencing of CHPV from vectors, reservoir hosts, and humans, beyond outbreak investigations, would help uncover cryptic transmission cycles and resolve uncertainties around viral maintenance.

Phylogenetic analyses revealed clear geographic structuring of CHPV, with distinct clustering of Indian and African lineages, suggesting long-term regional separation and limited recent gene flow between these regions. The grouping of the Nigerian hedgehog isolate with Senegalese strains, along with the presence of distinct subclades within Africa, points to localized diversification within African transmission cycles. In contrast, the clustering of recent Indian isolates with earlier strains indicates persistence of a relatively conserved lineage over time. This pattern is consistent with strong evolutionary constraint and limited sequence divergence within the available Indian lineage. The biological consequences of this evolutionary pattern remain unclear. Comparative analysis with other rhabdoviruses consistently placed CHPV closest to ISFV, supporting its phylogenetic placement within the vesiculovirus group. Although gene-wise trees broadly recapitulated whole-genome patterns, differences in resolution across loci highlight the importance of genome-wide data for resolving fine-scale phylogeographic relationships.

Root-to-tip regression analyses indicated a moderate temporal signal across the CHPV datasets, suggesting partial clock-like behaviour with some variation among genomic regions. Consistent with this, Bayesian skyline analyses under both relaxed and strict molecular clock models revealed largely stable population dynamics over time, with no clear evidence of sustained demographic change. However, the broad uncertainty associated with these estimates and the lack of consistent trends across gene-wise analyses highlight the limited resolution of the dataset. Collectively, these findings support the use of molecular clock-based approaches for CHPV while emphasizing cautious interpretation of evolutionary timelines and demographic inferences due to dataset limitations.

The low genetic divergence within the Indian clade (range: 1.48%–3.02%) reflects strong evolutionary constraints and suggests recent common ancestry among these isolates, consistent with epidemiological patterns showing localized circulation of CHPV in India. In contrast, the greater genetic heterogeneity observed in the African clade (range: 0.00%–27.93%) may reflect a combination of broader temporal sampling, geographic heterogeneity, host differences, and uneven sampling. The pronounced genetic differentiation between Indian and African lineages (range: 27.20%–28.51%) further highlights the importance of geographic isolation and ecological specialization in shaping CHPV evolution. This strong phylogeographic structuring has important implications for molecular epidemiology, vaccine design, and diagnostic development, as lineage-specific genetic variations could influence viral phenotypes, antigenicity, and transmission characteristics.

Episodic diversifying selection detected in glycoprotein, matrix protein, and nucleoprotein genes underscores the virus’s capacity for adaptive evolution, despite pervasive purifying selection across the genome. Mapping of these sites onto predicted B-cell epitopes further highlighted regions of immunological relevance. All three glycoprotein diversifying sites (positions 272, 424, and 503) overlapped with predicted B-cell epitopes (epitopes E9, E14, and E15), raising the possibility that some of these sites may be relevant to antigenic variation. Both matrix protein sites (positions 20 and 97) showed overlap with epitopes (E1 and E4). In contrast, among the two nucleoprotein diversifying sites, only position 187 (epitope E8) overlapped with a predicted epitope, while position 35 (N35E) was outside predicted antigenic domains. These findings indicate that immunogenic pressures vary among structural proteins. These findings are consistent with prior studies showing that surface glycoproteins in rhabdoviruses are subject to greater antigenic selection than internal structural proteins.

Together, these results emphasize the importance of routine and expanded genomic surveillance of CHPV. Current genomic data are heavily skewed toward outbreak-associated human isolates, leaving critical gaps in our understanding of viral diversity in vectors and potential wildlife reservoirs. Sequencing CHPV from circulating, non-outbreak strains, across regions, hosts, and time periods, would enhance phylogeographic analyses, enable early detection of emerging variants, and inform vaccine and diagnostic design. In addition, integrating genomic data with ecological and epidemiological surveillance could improve outbreak prediction and guide vector control strategies in endemic and at-risk regions.

Finally, although CHPV appears to evolve slowly at the whole-genome level, minor variation in antigenically exposed regions may impact vaccine efficacy or diagnostic performance. Continuous monitoring of structural genes under selection, especially those with epitope overlap, is essential for anticipating potential antigenic variation and updating serological tools. As sequencing becomes more accessible, its application to CHPV monitoring from sandflies, reservoir hosts, and subclinical infections will be central to unravelling its transmission ecology and mitigating its public health threat.

## Research gaps and future perspectives

The structure of the available datasets inherently shapes our comparative analysis, and several gaps must be acknowledged when interpreting the observed lineage patterns. The following are the key research gaps:

(i) **Host bias in sequencing**: The African sequences are almost entirely derived from Phlebotomine sandflies (and one hedgehog) collected between 1966 and 2017, whereas the Indian sequences originate exclusively from human clinical cases sampled during outbreaks between 2003 and 2024. This limited representation, with only five human-derived strains available for analysis, may not fully capture the intrahost evolutionary pressures and genetic diversity present within human populations. These two sources reflect fundamentally different host-associated selective environments: vertebrate isolates are shaped by adaptive immune pressures, while specific features of sandfly biology constrain vector-derived viruses. Consequently, the lineages detected in humans represent only those variants able to traverse vertebrate immune defences. In contrast, additional viral diversity may persist within vectors but remain unsampled or may have gone extinct.
(ii) **Sampling bias due to outbreak-associated bottlenecks**: The Indian dataset is dominated by outbreak-associated bottlenecks, capturing only those CHPV variants capable of causing symptomatic human disease. In endemic settings where adult populations are likely seropositive and asymptomatic, these outbreak isolates may represent relatively rare escape or spillover-competent variants, rather than the complete diversity circulating in India.
(iii) **Temporal and geographic confounding**: The African sequences span over five decades, while the Indian human isolates are concentrated in the past two decades. This means that some of the apparent divergence may reflect temporal separation rather than purely geographic structure. Similarly, reservoir systems differ. Hedgehogs are established hosts in Africa, whereas the primary reservoir in India remains uncertain, introducing ecological and environmental variation that cannot be fully ascertained with current data.
(iv) **Unconfirmed immune selection on diversifying sites**: Although episodic diversifying sites were identified, these signals cannot be confidently attributed to immune-relevant selection without paired human and vector sequences from the same region and timeframe. Considering these constraints, a more conservative interpretation is that our analyses reveal deep lineage separation between African vector-associated CHPV and Indian human-associated CHPV, while recognizing that observed diversity patterns likely arise from a combination of temporal differences, host-specific filters, ecological variation, and uneven sampling, rather than reflecting intrinsic differences in evolutionary rate. Nonetheless, our analysis robustly demonstrates this consistent lineage divergence, providing an important foundation for understanding global CHPV diversity and highlighting the need for integrated, longitudinal genomic surveillance across hosts and ecosystems.

## Conclusions

This study provides an integrated perspective on CHPV epidemiology, evolution, and antigenic potential. District-level case mapping and sandfly distribution analyses support vector associations and identify geographic hotspots. In contrast, phylogenetic analyses revealed deep geographic structuring between Indian and African strains, with relatively low genetic diversity among the available Indian human-derived isolates. Gene-level analyses highlight differential evolutionary pressures across structural proteins, with glycoprotein, matrix, and nucleoprotein exhibiting episodic diversifying sites, several of which overlapped predicted B-cell epitopes, revealing regions of immunological significance.

The findings emphasize the necessity of systematic genomic surveillance of CHPV across hosts, vectors, and geographic regions, including non-outbreak-associated viruses. Routine sequencing can provide critical insights into viral evolution, cryptic circulation, and potential antigenic changes. Applications include improved diagnostics, informed vaccine design, refined phylogeographic and evolutionary models, and early warning for outbreak preparedness. Expanded sequencing across multiple hosts and vectors will thus enhance our understanding of CHPV ecology and support proactive public health interventions.

## Supporting information

S1 Table Comparative overview of Chandipura virus and related Rhabdoviruses used as outgroups in its phylogenetic analysis, all characterized by negative-sense single-stranded RNA genomes and bullet-shaped virions. (DOCX)

S2 Table Reference genomes of related rhabdoviruses used as outgroups in the phylogenetic analysis of Chandipura virus whole genomes. (DOCX)

S3 Table Summary of confirmed Chandipura virus cases reported from different regions of India. (DOCX)

S4 Table Codon sites under episodic diversifying selection identified by MEME in the glycoprotein, matrix, and nucleocapsid proteins of Chandipura virus. (DOCX)

S5 Table Comprehensive site-by-site MEME analysis of the Chandipura virus (CHPV) glycoprotein gene identifying codons under episodic diversifying selection. (DOCX)

S6 Table Comprehensive site-by-site MEME analysis of the Chandipura virus matrix protein gene identifying codons under episodic diversifying selection. (DOCX)

S7 Table Comprehensive site-by-site MEME analysis of the Chandipura virus nucleoprotein gene identifying codons under episodic diversifying selection. (DOCX)

S1 Fig Temporal signal analysis of Chandipura virus (CHPV) sequences using TempEst. Root-to-tip regression plots showing the correlation between genetic divergence and sampling time for CHPV sequences. Panels depict: (A) whole genome, (B) glycoprotein (G), (C) matrix protein (M), (D) nucleoprotein (N), (E) phosphoprotein (P), and (F) large protein (L). Temporal signal was assessed to evaluate the suitability of the datasets for molecular clock analyses, with root-to-tip correlation coefficients indicated for each panel.
(DOCX)

S2 Fig Bayesian skyline plots of Chandipura virus protein-coding genes inferred under a relaxed molecular clock model. Bayesian skyline reconstructions of effective population size (N_e_τ) for individual Chandipura virus (CHPV) protein-coding genes inferred under an uncorrelated lognormal relaxed molecular clock model using BEAST 2.7.7. Panels represent: (A) glycoprotein (G), (B) matrix protein (M), (C) nucleoprotein (N), (D) phosphoprotein (P), and (E) large polymerase protein (L). In each panel, the solid line represents the median estimate, and the shaded region indicates the 95% highest posterior density (HPD) interval. The x-axis denotes time (years), and the y-axis represents scaled effective population size (N_e_τ).
(DOCX)

S3 Fig Bayesian skyline plots of Chandipura virus genomes and protein-coding genes inferred under a strict molecular clock model. Bayesian skyline reconstructions of effective population size (N_e_τ) for the Chandipura virus (CHPV) whole genome and individual protein-coding genes inferred under a strict molecular clock model using BEAST 2.7.7. Panels represent: (A) whole genome, (B) glycoprotein (G), (C) matrix protein (M), (D) nucleoprotein (N), (E) phosphoprotein (P), and (F) large polymerase protein (L). In each panel, the solid line represents the median estimate, and the shaded region indicates the 95% highest posterior density (HPD) interval. The x-axis denotes time (years), and the y-axis represents scaled effective population size (N_e_τ).
(DOCX)
