# Supplementary material for: Unravelling the genomic epidemiology and antigenic evolution of Chandipura virus: A genetically constrained neurotropic threat
Source: PLoS Negl Trop Dis. 2026 Aug 3;20(8):e0014565. doi: 10.1371/journal.pntd.0014565 (PMC13489527; doi:10.1371/journal.pntd.0014565)
Supplement: Supplementary file 1 [file pntd.0014565.s001.docx]

**S1 Table. Comparative overview of Chandipura virus and related Rhabdoviruses used as outgroups in its phylogenetic analysis, all characterized by negative-sense single-stranded RNA genomes and bullet-shaped virions.**

| **Virus** | **Family** | **Genus** | **Genome Length** | **Pathogenesis** | **Transmission Mode** | **Hosts** | **Disease** | **Symptoms** | **Detection Methods** |
| --- | --- | --- | --- | --- | --- | --- | --- | --- | --- |
| Chandipura | Rhabdoviridae | Vesiculovirus | ~11.1 kb | Mild to severe encephalitis, often fatal in children | Possibly sandfly | Primary: Humans (esp. children), sandflies; Potential reservoirs: Hedgehogs, rodents | Acute encephalitis | Sudden high fever, vomiting, convulsions, altered mental status, rapid coma/death (children) | RT-PCR, Real-time RT-PCR, virus isolation, immunofluorescence, ELISA |
| VS-NJ | Rhabdoviridae | Vesiculovirus | ~11.1 kb | Vesicular lesions, flu-like symptoms | Black flies, sandflies, direct contact | Horses, cattle, pigs, wildlife | Vesicular stomatitis | Fever, oral vesicles, ulcers on nose/hooves, lameness | RT-PCR, virus isolation, ELISA |
| VS-IND | Rhabdoviridae | Vesiculovirus | ~11.1 kb | Vesicular lesions, flu-like symptoms | Black flies, sandflies, direct contact | Horses, cattle, pigs | Vesicular stomatitis | Fever, excessive salivation, oral/hoof blisters, lameness | RT-PCR, virus isolation, ELISA |
| Isfahan | Rhabdoviridae | Vesiculovirus | ~11.1 kb | Mild febrile illness | Sandfly | Humans, gerbils, rodents | Febrile illness | Mild fever, headache; occasionally subclinical | Neutralization tests, PCR, virus isolation |
| Piry | Rhabdoviridae | Vesiculovirus | ~11.1 kb | Mild vesicular lesions | Possibly mosquitoes/sandflies | Rodents, rarely humans | Mild vesicular/febrile illness | Mild fever, malaise (human cases very rare) | Neutralizing antibody, PCR, isolation |
| Rabies | Rhabdoviridae | Lyssavirus | ~12 kb | Severe encephalitis, fatal | Mammalian bite/saliva | Mammals (bats, dogs, cats, humans) | Rabies encephalitis | Fever, pain/itch at site, anxiety, agitation, hydrophobia, paralysis, coma | Direct fluorescent antibody test, RT-PCR, ELISA, virus isolation |
