# Supplementary material for: Unravelling the genomic epidemiology and antigenic evolution of Chandipura virus: A genetically constrained neurotropic threat
Source: PLoS Negl Trop Dis. 2026 Aug 3;20(8):e0014565. doi: 10.1371/journal.pntd.0014565 (PMC13489527; doi:10.1371/journal.pntd.0014565)
Supplement: Supplementary file 2 [file pntd.0014565.s002.docx]

**S2 Table. Reference genomes of related rhabdoviruses used as outgroups in the phylogenetic analysis of Chandipura virus whole genomes.**

| **NCBI Accession Number** | **Virus Name** | **Genome Length (bp)** |
| --- | --- | --- |
| NC_001560.1 | Vesicular stomatitis Indiana virus | 11161 |
| MF196237.1 | Vesicular stomatitis New Jersey virus | 11107 |
| NC_020806.1 | Isfahan virus | 11088 |
| NC_038286.1 | Piry virus | 11271 |
| NC_001542.1 | Rabies virus | 11932 |
