# Supplementary material for: Unravelling the genomic epidemiology and antigenic evolution of Chandipura virus: A genetically constrained neurotropic threat
Source: PLoS Negl Trop Dis. 2026 Aug 3;20(8):e0014565. doi: 10.1371/journal.pntd.0014565 (PMC13489527; doi:10.1371/journal.pntd.0014565)
Supplement: Supplementary file 3 [file pntd.0014565.s003.docx]

**S3 Table. Summary of confirmed Chandipura virus cases reported from different regions of India.**

| **State** | **District / Region** | **No. of confirmed CHPV cases** | **Data collection month/year** | **Reference** |
| --- | --- | --- | --- | --- |
| **Gujarat** | Sabarkantha | 6 | June to August, 2024 | 1 |
|  | Panchmahal | 6 | June to August, 2024 | 2 |
|  | Mehsana | 4 | June to August, 2024 | 3 |
|  | Aravalli | 3 | June to August, 2024 | 4 |
|  | Kheda | 3 | June to August, 2024 | 5 |
|  | Ahmedabad (city) | 3 | June to August, 2024 | 6 |
|  | Dahod | 2 | June to August, 2024 | 7 |
|  | Mahisagar | 1 | June to August, 2024 | 8 |
|  | Surendranagar | 1 | June to August, 2024 | 9 |
|  | Gandhinagar | 1 | June to August, 2024 | 10 |
|  | Jamnagar | 1 | June to August, 2024 | 11 |
|  | Morbi | 1 | June to August, 2024 | 12 |
|  | Vadodara | 1 | June to August, 2024 | 13 |
|  | Banaskantha | 1 | June to August, 2024 | 14 |
|  | Devbhumi Dwarka | 1 | June to August, 2024 | 15 |
|  | Kutch | 1 | June to August, 2024 | 16 |
|  | Vadodara | 22 | 2005 | 17 |
|  | Panchmahal | 4 | 2005 | 18 |
| **Rajasthan** | Udaipur | 1 | June to August, 2024 | 19 |
|  | Dungarpur | 1 | June to August, 2024 | 20 |
|  | Shahpura (Bhilwara) | 1 | June to August, 2024 | 21 |
| **Bihar** | Singha par village of Neemachak Bathani block, Gaya district, Bihar | 1 | June, 2016 | 22 |
| **Odisha** | Gudrigaon village, Daringibadi block, Kandhmal district, Odisha | 4 | 17 to 24 September, 2009 | 23 |
| **Andhra Pradesh** | Warangal | 46 | May 2005 to April 2006, June to September, 2003 | 24 |
|  | Karimnagar | 17 | May 2005 to April 2006, June to September, 2003 | 25 |
|  | Khammam | 1 | May 2005 to April 2006, June to September, 2003 | 26 |
|  | Adilabad | 1 | May 2005 to April 2006, June to September, 2003 | 27 |
|  | Nizamabad | 5 | June to September, 2003 | 28 |
|  | Medak | 2 | June to September, 2003 | 29 |
|  | Nalgonda | 2 | June to September, 2003 | 30 |
|  | Mehboob nagar | 2 | June to September, 2003 | 31 |
|  | Nellore | 2 | June to September, 2003 | 32 |
| **Maharashtra** | Nagpur division in Maharashtra State |  | June to September, 2007 | 33 |
|  | Bhandara | 12 | June to September, 2007 | 34 |
|  | Wardha | 11 | June to September, 2007 | 35 |
|  | Nagpur | 4 | June to September, 2007 | 36 |
|  | Gondia | 6 | June to September, 2007 | 37 |
|  | Chandrapur | 2 | June to September, 2007 | 38 |
|  | Gadchiroli | 4 | June to September, 2007 | 39 |
| **Madhya Pradesh** | Jabalpur | 1 | 1983 | 40 |

**References**

1. https://www.hindustantimes.com/india-news/gujarat-records-48-aes-deaths-39-confirmed-cases-of-chandipura-virus-101722066014164.html. 2024
2. https://www.hindustantimes.com/india-news/gujarat-records-48-aes-deaths-39-confirmed-cases-of-chandipura-virus-101722066014164.html. 2024
3. https://www.hindustantimes.com/india-news/gujarat-records-48-aes-deaths-39-confirmed-cases-of-chandipura-virus-101722066014164.html. 2024
4. https://www.hindustantimes.com/india-news/gujarat-records-48-aes-deaths-39-confirmed-cases-of-chandipura-virus-101722066014164.html. 2024
5. https://www.hindustantimes.com/india-news/gujarat-records-48-aes-deaths-39-confirmed-cases-of-chandipura-virus-101722066014164.html. 2024
6. https://www.hindustantimes.com/india-news/gujarat-records-48-aes-deaths-39-confirmed-cases-of-chandipura-virus-101722066014164.html. 2024
7. https://www.hindustantimes.com/india-news/gujarat-records-48-aes-deaths-39-confirmed-cases-of-chandipura-virus-101722066014164.html. 2024
8. https://www.thehindu.com/news/national/chandipura-virus-death-toll-rises-to-15-as-the-virus-spreads-in-more-districts-in-gujarat/article68415494.ece. 2024
9. https://www.thehindu.com/news/national/chandipura-virus-death-toll-rises-to-15-as-the-virus-spreads-in-more-districts-in-gujarat/article68415494.ece. 2024
10. https://www.thehindu.com/news/national/chandipura-virus-death-toll-rises-to-15-as-the-virus-spreads-in-more-districts-in-gujarat/article68415494.ece. 2024
11. https://www.thehindu.com/news/national/chandipura-virus-death-toll-rises-to-15-as-the-virus-spreads-in-more-districts-in-gujarat/article68415494.ece. 2024
12. https://www.thehindu.com/news/national/chandipura-virus-death-toll-rises-to-15-as-the-virus-spreads-in-more-districts-in-gujarat/article68415494.ece. 2024
13. https://www.thehindu.com/news/national/chandipura-virus-death-toll-rises-to-15-as-the-virus-spreads-in-more-districts-in-gujarat/article68415494.ece. 2024
14. https://www.thehindu.com/news/national/chandipura-virus-death-toll-rises-to-15-as-the-virus-spreads-in-more-districts-in-gujarat/article68415494.ece. 2024
15. https://www.thehindu.com/news/national/chandipura-virus-death-toll-rises-to-15-as-the-virus-spreads-in-more-districts-in-gujarat/article68415494.ece. 2024
16. https://www.thehindu.com/news/national/chandipura-virus-death-toll-rises-to-15-as-the-virus-spreads-in-more-districts-in-gujarat/article68415494.ece. 2024
17. Chadha, M. S., Arankalle, V. A., Jadi, R. S., Joshi, M. V., Thakare, J. P., Mahadev, P. V., & Mishra, A. C. (2005). An outbreak of Chandipura virus encephalitis in the eastern districts of Gujarat state, India. The American journal of tropical medicine and hygiene, 73(3), 566–570. 2005
18. Chadha, M. S., Arankalle, V. A., Jadi, R. S., Joshi, M. V., Thakare, J. P., Mahadev, P. V., & Mishra, A. C. (2005). An outbreak of Chandipura virus encephalitis in the eastern districts of Gujarat state, India. The American journal of tropical medicine and hygiene, 73(3), 566–570. 2005
19. https://rajasthan.ndtv.in/rajasthan-news/second-death-due-to-chandipura-virus-in-rajasthan-dead-body-of-2-year-old-girl-reached-itadiya-village-in-ppe-kit-6298602. 2024
20. https://timesofindia.indiatimes.com/city/jaipur/1st-case-of-chandipura-in-rajasthan-confirmed-3-year-old-in-dungarpur/articleshow/112105592.cms. 2024
21. https://rajasthan.ndtv.in/rajasthan-news/second-death-due-to-chandipura-virus-in-rajasthan-dead-body-of-2-year-old-girl-reached-itadiya-village-in-ppe-kit-6298602. 2024
22. Diwakar Singh Dinesh, Roshan Kamal Topno, Krishna Pandey, Rishikesh Kumar, Ganesh Chandra Sahoo, Maneesh Kumar, Banke Bihari Singh, Wakil Paswan and Pradeep Das. 2019. Chandipura Virus recognized among AES for the First Time in Bihar, India.Int.J.Curr.Microbiol.App.Sci. 7(12): 3658-3663. doi: https://doi.org/10.20546/ijcmas.2018.712.414. 2018
23. Dwibedi, B., Sabat, J., Hazra, R. K., Kumar, A., Dinesh, D. S., & Kar, S. K. (2015). Chandipura virus infection causing encephalitis in a tribal population of Odisha in eastern India. The National medical journal of India, 28(4), 185–187. ttps://pubmed.ncbi.nlm.nih.gov/27132726/. 2015
24. Tandale, B. V., Tikute, S. S., Arankalle, V. A., Sathe, P. S., Joshi, M. V., Ranadive, S. N., Kanojia, P. C., Eshwarachary, D., Kumarswamy, M., & Mishra, A. C. (2008). Chandipura virus: a major cause of acute encephalitis in children in North Telangana, Andhra Pradesh, India. Journal of medical virology, 80(1), 118–124. https://doi.org/10.1002/jmv.21041. 2008
25. Tandale, B. V., Tikute, S. S., Arankalle, V. A., Sathe, P. S., Joshi, M. V., Ranadive, S. N., Kanojia, P. C., Eshwarachary, D., Kumarswamy, M., & Mishra, A. C. (2008). Chandipura virus: a major cause of acute encephalitis in children in North Telangana, Andhra Pradesh, India. Journal of medical virology, 80(1), 118–124. https://doi.org/10.1002/jmv.21041. 2008
26. Tandale, B. V., Tikute, S. S., Arankalle, V. A., Sathe, P. S., Joshi, M. V., Ranadive, S. N., Kanojia, P. C., Eshwarachary, D., Kumarswamy, M., & Mishra, A. C. (2008). Chandipura virus: a major cause of acute encephalitis in children in North Telangana, Andhra Pradesh, India. Journal of medical virology, 80(1), 118–124. https://doi.org/10.1002/jmv.21041. 2008
27. Tandale, B. V., Tikute, S. S., Arankalle, V. A., Sathe, P. S., Joshi, M. V., Ranadive, S. N., Kanojia, P. C., Eshwarachary, D., Kumarswamy, M., & Mishra, A. C. (2008). Chandipura virus: a major cause of acute encephalitis in children in North Telangana, Andhra Pradesh, India. Journal of medical virology, 80(1), 118–124. https://doi.org/10.1002/jmv.21041. 2008
28. Rao, B. L., Basu, A., Wairagkar, N. S., Gore, M. M., Arankalle, V. A., Thakare, J. P., Jadi, R. S., Rao, K. A., & Mishra, A. C. (2004). A large outbreak of acute encephalitis with high fatality rate in children in Andhra Pradesh, India, in 2003, associated with Chandipura virus. Lancet (London, England), 364(9437), 869–874. https://doi.org/10.1016/S0140-6736(04)16982-1. 2004
29. Rao, B. L., Basu, A., Wairagkar, N. S., Gore, M. M., Arankalle, V. A., Thakare, J. P., Jadi, R. S., Rao, K. A., & Mishra, A. C. (2004). A large outbreak of acute encephalitis with high fatality rate in children in Andhra Pradesh, India, in 2003, associated with Chandipura virus. Lancet (London, England), 364(9437), 869–874. https://doi.org/10.1016/S0140-6736(04)16982-1. 2004
30. Rao, B. L., Basu, A., Wairagkar, N. S., Gore, M. M., Arankalle, V. A., Thakare, J. P., Jadi, R. S., Rao, K. A., & Mishra, A. C. (2004). A large outbreak of acute encephalitis with high fatality rate in children in Andhra Pradesh, India, in 2003, associated with Chandipura virus. Lancet (London, England), 364(9437), 869–874. https://doi.org/10.1016/S0140-6736(04)16982-1. 2004
31. Rao, B. L., Basu, A., Wairagkar, N. S., Gore, M. M., Arankalle, V. A., Thakare, J. P., Jadi, R. S., Rao, K. A., & Mishra, A. C. (2004). A large outbreak of acute encephalitis with high fatality rate in children in Andhra Pradesh, India, in 2003, associated with Chandipura virus. Lancet (London, England), 364(9437), 869–874. https://doi.org/10.1016/S0140-6736(04)16982-1. 2004
32. Rao, B. L., Basu, A., Wairagkar, N. S., Gore, M. M., Arankalle, V. A., Thakare, J. P., Jadi, R. S., Rao, K. A., & Mishra, A. C. (2004). A large outbreak of acute encephalitis with high fatality rate in children in Andhra Pradesh, India, in 2003, associated with Chandipura virus. Lancet (London, England), 364(9437), 869–874. https://doi.org/10.1016/S0140-6736(04)16982-1. 2004
33. Gurav, Y. K., Tandale, B. V., Jadi, R. S., Gunjikar, R. S., Tikute, S. S., Jamgaonkar, A. V., Khadse, R. K., Jalgaonkar, S. V., Arankalle, V. A., & Mishra, A. C. (2010). Chandipura virus encephalitis outbreak among children in Nagpur division, Maharashtra, 2007. The Indian journal of medical research, 132, 395–399. 2010
34. Gurav, Y. K., Tandale, B. V., Jadi, R. S., Gunjikar, R. S., Tikute, S. S., Jamgaonkar, A. V., Khadse, R. K., Jalgaonkar, S. V., Arankalle, V. A., & Mishra, A. C. (2010). Chandipura virus encephalitis outbreak among children in Nagpur division, Maharashtra, 2007. The Indian journal of medical research, 132, 395–399. 2010
35. Gurav, Y. K., Tandale, B. V., Jadi, R. S., Gunjikar, R. S., Tikute, S. S., Jamgaonkar, A. V., Khadse, R. K., Jalgaonkar, S. V., Arankalle, V. A., & Mishra, A. C. (2010). Chandipura virus encephalitis outbreak among children in Nagpur division, Maharashtra, 2007. The Indian journal of medical research, 132, 395–399. 2010
36. Gurav, Y. K., Tandale, B. V., Jadi, R. S., Gunjikar, R. S., Tikute, S. S., Jamgaonkar, A. V., Khadse, R. K., Jalgaonkar, S. V., Arankalle, V. A., & Mishra, A. C. (2010). Chandipura virus encephalitis outbreak among children in Nagpur division, Maharashtra, 2007. The Indian journal of medical research, 132, 395–399. 2010
37. Gurav, Y. K., Tandale, B. V., Jadi, R. S., Gunjikar, R. S., Tikute, S. S., Jamgaonkar, A. V., Khadse, R. K., Jalgaonkar, S. V., Arankalle, V. A., & Mishra, A. C. (2010). Chandipura virus encephalitis outbreak among children in Nagpur division, Maharashtra, 2007. The Indian journal of medical research, 132, 395–399. 2010
38. Gurav, Y. K., Tandale, B. V., Jadi, R. S., Gunjikar, R. S., Tikute, S. S., Jamgaonkar, A. V., Khadse, R. K., Jalgaonkar, S. V., Arankalle, V. A., & Mishra, A. C. (2010). Chandipura virus encephalitis outbreak among children in Nagpur division, Maharashtra, 2007. The Indian journal of medical research, 132, 395–399. 2010
39. Gurav, Y. K., Tandale, B. V., Jadi, R. S., Gunjikar, R. S., Tikute, S. S., Jamgaonkar, A. V., Khadse, R. K., Jalgaonkar, S. V., Arankalle, V. A., & Mishra, A. C. (2010). Chandipura virus encephalitis outbreak among children in Nagpur division, Maharashtra, 2007. The Indian journal of medical research, 132, 395–399. 2010
40. Rodrigues JR, Singh PB, Dave DS, Prasan R, Ayachit VL, et al. 1983. Isolation of Chandipura virus from the blood in acute encephalopathy syndrome. Indian J Med Res 77:303–307. 1983
