# Supplementary material for: Unravelling the genomic epidemiology and antigenic evolution of Chandipura virus: A genetically constrained neurotropic threat
Source: PLoS Negl Trop Dis. 2026 Aug 3;20(8):e0014565. doi: 10.1371/journal.pntd.0014565 (PMC13489527; doi:10.1371/journal.pntd.0014565)
Supplement: Supplementary file 4 [file pntd.0014565.s004.docx]

**S4 Table. Codon Sites Under Episodic Diversifying Selection Identified by MEME in the Glycoprotein, Matrix, and Nucleocapsid Proteins of Chandipura Virus.**

| **Gene** | **Codon Position** | **Nucleotide Change** | **Amino Acid Change** | **Variant Prevalence (%)** | **Possible implications** |
| --- | --- | --- | --- | --- | --- |
| Glycoprotein | 272 | CCA → CAT | Proline (P) → Histidine (H) | 4.35% | Non-conservative: May affect local structure due to change from rigid to polar/basic residue |
|  | 424 | TTG → TGG | Leucine (L) → Tryptophan (W) | 4.35% | Non-conservative: Large aromatic side chain; could impact protein folding or binding |
|  | 503 | AAG → AGG | Lysine (K) → Arginine (R) | 17.39% | Semi-conservative: Both basic, but differ in charge distribution and flexibility |
| Matrix protein | 20 | AAA → AGA | Lysine (K) → Arginine (R) | 34.78% | Semi-conservative: Both are basic, positively charged residues; minimal impact on structure or local interactions is expected. |
|  | 97 | GAT → GAC / AAC | Aspartic acid (D) → Aspartic acid (D) / Asparagine (N) | 4.35% | Mostly conservative: D is preserved with GAT→GAC; D→N is semi-conservative (acidic to polar), which could slightly affect local interactions. |
| Nucleoprotein | 35 | AAT → GAG | Asparagine (N) → Glutamic acid (E) | 21.74% | Non-conservative: Polar, uncharged asparagine replaced by negatively charged glutamic acid, which could affect local structure and protein interactions. |
|  | 187 | GCA → GTG | Alanine (A) → Valine (V) | 4.35% | Conservative: Both are small, nonpolar residues; slight increase in side-chain bulk could modestly affect local packing but is unlikely to drastically alter protein structure. |
