# Supplementary material for: Unravelling the genomic epidemiology and antigenic evolution of Chandipura virus: A genetically constrained neurotropic threat
Source: PLoS Negl Trop Dis. 2026 Aug 3;20(8):e0014565. doi: 10.1371/journal.pntd.0014565 (PMC13489527; doi:10.1371/journal.pntd.0014565)
Supplement: Supplementary file 5 [file pntd.0014565.s005.docx]

**S5_Table. Comprehensive site-by-site MEME analysis of the Chandipura virus (CHPV) glycoprotein gene identifying codons under episodic diversifying selection.**

| **Part.** | **Codon** | **α** | **β1** | **p1** | **β+** | **p+** | **LRT** | **p-value** | **# branches under selection** | **q** | **Class** |
| --- | --- | --- | --- | --- | --- | --- | --- | --- | --- | --- | --- |
| 1 | 1 | 0 | 0 | 1 | 0 | 0 | 0 | 1 | 0 | 1 | Invariable |
| 1 | 2 | 0.001 | 0 | 0 | 15.523 | 1 | 2.984 | 0.108 | 1 | 1 | Neutral |
| 1 | 3 | 3.937 | 0 | 0 | 1.505 | 1 | 0 | 0.667 | 0 | 1 | Neutral |
| 1 | 4 | 4.115 | 0 | 0 | 49.632 | 1 | 2.736 | 0.123 | 1 | 1 | Neutral |
| 1 | 5 | 0 | 0 | 0 | 1.888 | 1 | 1.393 | 0.254 | 1 | 1 | Neutral |
| 1 | 6 | 9.45 | 0 | 0 | 77.284 | 1 | 1.839 | 0.199 | 2 | 1 | Neutral |
| 1 | 7 | 0 | 0 | 0 | 3.76 | 1 | 1.904 | 0.192 | 0 | 1 | Neutral |
| 1 | 8 | 0 | 0 | 0 | 7.036 | 1 | 0.69 | 0.382 | 0 | 1 | Neutral |
| 1 | 9 | 6.245 | 0.998 | 0 | 24.983 | 1 | 0.623 | 0.397 | 0 | 1 | Neutral |
| 1 | 10 | 2.087 | 1.799 | 0 | 2.065 | 1 | 0 | 0.667 | 0 | 1 | Neutral |
| 1 | 11 | 0 | 0 | 0 | 2.508 | 1 | 1.951 | 0.187 | 0 | 1 | Neutral |
| 1 | 12 | 0 | 0 | 0 | 40.624 | 1 | 1.934 | 0.189 | 0 | 1 | Neutral |
| 1 | 13 | 10.899 | 5.682 | 0 | 6.331 | 1 | 0 | 0.667 | 0 | 1 | Neutral |
| 1 | 14 | 2.763 | 2.598 | 0 | 2.608 | 1 | 0 | 0.667 | 0 | 1 | Neutral |
| 1 | 15 | 3.862 | 0.326 | 0 | 4.274 | 1 | 0.01 | 0.64 | 0 | 1 | Neutral |
| 1 | 16 | 3.17 | 0 | 0 | 0.738 | 1 | 0 | 0.667 | 0 | 1 | Neutral |
| 1 | 17 | 3.012 | 0 | 0 | 15.756 | 1 | 0.896 | 0.337 | 2 | 1 | Neutral |
| 1 | 18 | 10.188 | 0 | 0 | 23.385 | 1 | 0.386 | 0.463 | 2 | 1 | Neutral |
| 1 | 19 | 29.215 | 2.197 | 0 | 2.958 | 1 | 0 | 0.667 | 0 | 1 | Neutral |
| 1 | 20 | 0.007 | 0.004 | 0 | 22.864 | 1 | 1.88 | 0.194 | 1 | 1 | Neutral |
| 1 | 21 | 0 | 0 | 1 | 0 | 0 | 0 | 1 | 0 | 1 | Invariable |
| 1 | 22 | 22.442 | 0 | 0 | 2.422 | 1 | 0 | 0.667 | 0 | 1 | Neutral |
| 1 | 23 | 2.563 | 0 | 0 | 1.094 | 1 | 0 | 0.667 | 0 | 1 | Neutral |
| 1 | 24 | 0 | 0 | 1 | 0 | 0 | 0 | 1 | 0 | 1 | Invariable |
| 1 | 25 | 0 | 0 | 1 | 0 | 0 | 0 | 1 | 0 | 1 | Invariable |
| 1 | 26 | 14.778 | 0 | 0 | 4.559 | 1 | 0 | 0.667 | 0 | 1 | Neutral |
| 1 | 27 | 64.712 | 0 | 0 | 2.934 | 1 | 0 | 0.667 | 0 | 1 | Neutral |
| 1 | 28 | 4.408 | 0 | 0 | 0.908 | 1 | 0 | 0.667 | 0 | 1 | Neutral |
| 1 | 29 | 6.638 | 0 | 0 | 1.197 | 1 | 0 | 0.667 | 0 | 1 | Neutral |
| 1 | 30 | 3.691 | 0.715 | 0 | 0.566 | 1 | 0 | 0.667 | 0 | 1 | Neutral |
| 1 | 31 | 8.771 | 0 | 0 | 2.289 | 1 | 0 | 0.667 | 0 | 1 | Neutral |
| 1 | 32 | 7.011 | 0 | 0 | 1.162 | 1 | 0 | 0.667 | 0 | 1 | Neutral |
| 1 | 33 | 8.284 | 0 | 0 | 1.281 | 1 | 0 | 0.667 | 0 | 1 | Neutral |
| 1 | 34 | 2.628 | 0 | 0 | 0.449 | 1 | 0 | 0.667 | 0 | 1 | Neutral |
| 1 | 35 | 0 | 0 | 1 | 0 | 0 | 0 | 1 | 0 | 1 | Invariable |
| 1 | 36 | 3.205 | 0 | 0 | 0.426 | 1 | 0 | 0.667 | 0 | 1 | Neutral |
| 1 | 37 | 6.457 | 0 | 0 | 1.103 | 1 | 0 | 0.667 | 0 | 1 | Neutral |
| 1 | 38 | 2.96 | 0 | 0 | 0.782 | 1 | 0 | 0.667 | 0 | 1 | Neutral |
| 1 | 39 | 16.667 | 0 | 0 | 2.869 | 1 | 0 | 0.667 | 0 | 1 | Neutral |
| 1 | 40 | 6.174 | 0 | 0 | 157.733 | 1 | 1.904 | 0.192 | 1 | 1 | Neutral |
| 1 | 41 | 6.849 | 0 | 0 | 1.363 | 1 | 0 | 0.667 | 0 | 1 | Neutral |
| 1 | 42 | 2.914 | 0 | 0 | 0.799 | 1 | 0 | 0.667 | 0 | 1 | Neutral |
| 1 | 43 | 8.539 | 0 | 0 | 1.589 | 1 | 0 | 0.667 | 0 | 1 | Neutral |
| 1 | 44 | 16.73 | 0 | 0 | 3.415 | 1 | 0 | 0.667 | 0 | 1 | Neutral |
| 1 | 45 | 3.285 | 0 | 0 | 0.176 | 1 | 0 | 0.667 | 0 | 1 | Neutral |
| 1 | 46 | 9.826 | 0 | 0 | 2.418 | 1 | 0 | 0.667 | 0 | 1 | Neutral |
| 1 | 47 | 0 | 0 | 1 | 0 | 0 | 0 | 1 | 0 | 1 | Invariable |
| 1 | 48 | 2.891 | 0 | 0 | 0.49 | 1 | 0 | 0.667 | 0 | 1 | Neutral |
| 1 | 49 | 12.7 | 0 | 0 | 2.281 | 1 | 0 | 0.667 | 0 | 1 | Neutral |
| 1 | 50 | 4.793 | 0 | 0 | 1.021 | 1 | 0 | 0.667 | 0 | 1 | Neutral |
| 1 | 51 | 0 | 0 | 1 | 0 | 0 | 0 | 1 | 0 | 1 | Invariable |
| 1 | 52 | 9.048 | 0 | 0 | 1.38 | 1 | 0 | 0.667 | 0 | 1 | Neutral |
| 1 | 53 | 13.634 | 0 | 0 | 4.34 | 1 | 0 | 0.667 | 0 | 1 | Neutral |
| 1 | 54 | 4.743 | 0 | 0 | 61.073 | 1 | 0.664 | 0.388 | 1 | 1 | Neutral |
| 1 | 55 | 16.965 | 0 | 0 | 2.683 | 1 | 0 | 0.667 | 0 | 1 | Neutral |
| 1 | 56 | 9.111 | 0 | 0 | 21.619 | 1 | 0.189 | 0.533 | 1 | 1 | Neutral |
| 1 | 57 | 15.895 | 0 | 0 | 2.8 | 1 | 0 | 0.667 | 0 | 1 | Neutral |
| 1 | 58 | 8.331 | 0 | 0 | 1.796 | 1 | 0 | 0.667 | 0 | 1 | Neutral |
| 1 | 59 | 4.4 | 0 | 0 | 0.873 | 1 | 0 | 0.667 | 0 | 1 | Neutral |
| 1 | 60 | 18.877 | 0 | 0 | 3.086 | 1 | 0 | 0.667 | 0 | 1 | Neutral |
| 1 | 61 | 5.167 | 0 | 0 | 1.099 | 1 | 0 | 0.667 | 0 | 1 | Neutral |
| 1 | 62 | 5.114 | 0 | 0 | 0.983 | 1 | 0 | 0.667 | 0 | 1 | Neutral |
| 1 | 63 | 6.393 | 0 | 0 | 13.481 | 1 | 0.324 | 0.483 | 0 | 1 | Neutral |
| 1 | 64 | 11.945 | 0 | 0 | 1.434 | 1 | 0 | 0.667 | 0 | 1 | Neutral |
| 1 | 65 | 2.836 | 0 | 0 | 0.15 | 1 | 0 | 0.667 | 0 | 1 | Neutral |
| 1 | 66 | 6.338 | 0 | 0 | 1.288 | 1 | 0 | 0.667 | 0 | 1 | Neutral |
| 1 | 67 | 25.195 | 0 | 0 | 3.509 | 1 | 0 | 0.667 | 0 | 1 | Neutral |
| 1 | 68 | 7.473 | 0 | 0 | 2.04 | 1 | 0 | 0.667 | 0 | 1 | Neutral |
| 1 | 69 | 3.527 | 0 | 0 | 0.808 | 1 | 0 | 0.667 | 0 | 1 | Neutral |
| 1 | 70 | 35.352 | 0 | 0 | 7.542 | 1 | 0 | 0.667 | 0 | 1 | Neutral |
| 1 | 71 | 1.896 | 0 | 0 | 0.312 | 1 | 0 | 0.667 | 0 | 1 | Neutral |
| 1 | 72 | 5.771 | 0 | 0 | 1.2 | 1 | 0 | 0.667 | 0 | 1 | Neutral |
| 1 | 73 | 12.911 | 0 | 0 | 2.452 | 1 | 0 | 0.667 | 0 | 1 | Neutral |
| 1 | 74 | 35.506 | 0 | 0 | 4.174 | 1 | 0 | 0.667 | 0 | 1 | Neutral |
| 1 | 75 | 2.248 | 0 | 0 | 0.439 | 1 | 0 | 0.667 | 0 | 1 | Neutral |
| 1 | 76 | 7.459 | 0 | 0 | 1.46 | 1 | 0 | 0.667 | 0 | 1 | Neutral |
| 1 | 77 | 3.286 | 0 | 0 | 0.857 | 1 | 0 | 0.667 | 0 | 1 | Neutral |
| 1 | 78 | 17.357 | 0 | 0 | 2.5 | 1 | 0 | 0.667 | 0 | 1 | Neutral |
| 1 | 79 | 14.394 | 0 | 0 | 1.586 | 1 | 0 | 0.667 | 0 | 1 | Neutral |
| 1 | 80 | 8.166 | 0 | 0 | 1.546 | 1 | 0 | 0.667 | 0 | 1 | Neutral |
| 1 | 81 | 6.63 | 0 | 0 | 1.197 | 1 | 0 | 0.667 | 0 | 1 | Neutral |
| 1 | 82 | 6.59 | 0 | 0 | 1.352 | 1 | 0 | 0.667 | 0 | 1 | Neutral |
| 1 | 83 | 14.347 | 0 | 0 | 2.62 | 1 | 0 | 0.667 | 0 | 1 | Neutral |
| 1 | 84 | 4.216 | 0 | 0 | 0.871 | 1 | 0 | 0.667 | 0 | 1 | Neutral |
| 1 | 85 | 0 | 0 | 1 | 0 | 0 | 0 | 1 | 0 | 1 | Invariable |
| 1 | 86 | 14.406 | 0 | 0 | 2.946 | 1 | 0 | 0.667 | 0 | 1 | Neutral |
| 1 | 87 | 6.678 | 0 | 0 | 1.334 | 1 | 0 | 0.667 | 0 | 1 | Neutral |
| 1 | 88 | 11.713 | 0 | 0 | 1.817 | 1 | 0 | 0.667 | 0 | 1 | Neutral |
| 1 | 89 | 10.382 | 0 | 0 | 1.292 | 1 | 0 | 0.667 | 0 | 1 | Neutral |
| 1 | 90 | 14.041 | 0 | 0 | 2.466 | 1 | 0 | 0.667 | 0 | 1 | Neutral |
| 1 | 91 | 3.547 | 0 | 0 | 0.798 | 1 | 0 | 0.667 | 0 | 1 | Neutral |
| 1 | 92 | 21.603 | 0 | 0 | 6.271 | 1 | 0 | 0.667 | 0 | 1 | Neutral |
| 1 | 93 | 0 | 0 | 1 | 0 | 0 | 0 | 1 | 0 | 1 | Invariable |
| 1 | 94 | 0 | 0 | 1 | 0 | 0 | 0 | 1 | 0 | 1 | Invariable |
| 1 | 95 | 2.173 | 0 | 0 | 0.357 | 1 | 0 | 0.667 | 0 | 1 | Neutral |
| 1 | 96 | 9.012 | 0 | 0 | 0.861 | 1 | 0 | 0.667 | 0 | 1 | Neutral |
| 1 | 97 | 19.384 | 0 | 0 | 3.133 | 1 | 0 | 0.667 | 0 | 1 | Neutral |
| 1 | 98 | 5.629 | 0 | 0 | 1.178 | 1 | 0 | 0.667 | 0 | 1 | Neutral |
| 1 | 99 | 3.005 | 0 | 0 | 0.704 | 1 | 0 | 0.667 | 0 | 1 | Neutral |
| 1 | 100 | 14.059 | 0 | 0 | 2.206 | 1 | 0 | 0.667 | 0 | 1 | Neutral |
| 1 | 101 | 2.618 | 0 | 0 | 0.623 | 1 | 0 | 0.667 | 0 | 1 | Neutral |
| 1 | 102 | 2.173 | 0 | 0 | 0.357 | 1 | 0 | 0.667 | 0 | 1 | Neutral |
| 1 | 103 | 11.628 | 0 | 0 | 3.199 | 1 | 0 | 0.667 | 0 | 1 | Neutral |
| 1 | 104 | 7.834 | 0 | 0 | 1.501 | 1 | 0 | 0.667 | 0 | 1 | Neutral |
| 1 | 105 | 9.048 | 0 | 0 | 1.38 | 1 | 0 | 0.667 | 0 | 1 | Neutral |
| 1 | 106 | 1.965 | 0 | 0 | 0.34 | 1 | 0 | 0.667 | 0 | 1 | Neutral |
| 1 | 107 | 0 | 0 | 1 | 0 | 0 | 0 | 1 | 0 | 1 | Invariable |
| 1 | 108 | 8.863 | 0 | 0 | 1.636 | 1 | 0 | 0.667 | 0 | 1 | Neutral |
| 1 | 109 | 4.21 | 0 | 0 | 0.484 | 1 | 0 | 0.667 | 0 | 1 | Neutral |
| 1 | 110 | 4.071 | 0 | 0 | 0.908 | 1 | 0 | 0.667 | 0 | 1 | Neutral |
| 1 | 111 | 0 | 0 | 1 | 0 | 0 | 0 | 1 | 0 | 1 | Invariable |
| 1 | 112 | 10.039 | 0 | 0 | 1.78 | 1 | 0 | 0.667 | 0 | 1 | Neutral |
| 1 | 113 | 47.58 | 0 | 0 | 2.305 | 1 | 0 | 0.667 | 0 | 1 | Neutral |
| 1 | 114 | 7.834 | 0 | 0 | 1.501 | 1 | 0 | 0.667 | 0 | 1 | Neutral |
| 1 | 115 | 17.496 | 0 | 0 | 9.523 | 1 | 0 | 0.667 | 0 | 1 | Neutral |
| 1 | 116 | 7.025 | 0 | 0 | 1.619 | 1 | 0 | 0.667 | 0 | 1 | Neutral |
| 1 | 117 | 9.253 | 0 | 0 | 2.513 | 1 | 0 | 0.667 | 0 | 1 | Neutral |
| 1 | 118 | 2.739 | 0 | 0 | 0.249 | 1 | 0 | 0.667 | 0 | 1 | Neutral |
| 1 | 119 | 14.669 | 0 | 0 | 4.998 | 1 | 0 | 0.667 | 0 | 1 | Neutral |
| 1 | 120 | 2.727 | 0 | 0 | 0.465 | 1 | 0 | 0.667 | 0 | 1 | Neutral |
| 1 | 121 | 3.003 | 0 | 0 | 0.704 | 1 | 0 | 0.667 | 0 | 1 | Neutral |
| 1 | 122 | 5.526 | 0.958 | 0 | 0.899 | 1 | 0 | 0.667 | 0 | 1 | Neutral |
| 1 | 123 | 8.217 | 0 | 0 | 1.812 | 1 | 0 | 0.667 | 0 | 1 | Neutral |
| 1 | 124 | 7.034 | 0 | 0 | 1.887 | 1 | 0 | 0.667 | 0 | 1 | Neutral |
| 1 | 125 | 14.44 | 0 | 0 | 2.641 | 1 | 0 | 0.667 | 0 | 1 | Neutral |
| 1 | 126 | 12.123 | 1.217 | 0 | 1.368 | 1 | 0 | 0.667 | 0 | 1 | Neutral |
| 1 | 127 | 17.461 | 0.753 | 0 | 0.925 | 1 | 0 | 0.667 | 0 | 1 | Neutral |
| 1 | 128 | 12.548 | 0 | 0 | 2.264 | 1 | 0 | 0.667 | 0 | 1 | Neutral |
| 1 | 129 | 9.292 | 0 | 0 | 2.093 | 1 | 0 | 0.667 | 0 | 1 | Neutral |
| 1 | 130 | 5.629 | 0 | 0 | 1.178 | 1 | 0 | 0.667 | 0 | 1 | Neutral |
| 1 | 131 | 13.478 | 0 | 0 | 2.624 | 1 | 0 | 0.667 | 0 | 1 | Neutral |
| 1 | 132 | 13.182 | 0 | 0 | 1.86 | 1 | 0 | 0.667 | 0 | 1 | Neutral |
| 1 | 133 | 5.202 | 0.802 | 0 | 0.76 | 1 | 0 | 0.667 | 0 | 1 | Neutral |
| 1 | 134 | 3.937 | 0 | 0 | 1.505 | 1 | 0 | 0.667 | 0 | 1 | Neutral |
| 1 | 135 | 0 | 0 | 1 | 0 | 0 | 0 | 1 | 0 | 1 | Invariable |
| 1 | 136 | 5.905 | 0 | 0 | 1.423 | 1 | 0 | 0.667 | 0 | 1 | Neutral |
| 1 | 137 | 0 | 0 | 1 | 0 | 0 | 0 | 1 | 0 | 1 | Invariable |
| 1 | 138 | 7.84 | 0 | 0 | 1.502 | 1 | 0 | 0.667 | 0 | 1 | Neutral |
| 1 | 139 | 7.389 | 0.525 | 0 | 3.501 | 1 | 0 | 0.667 | 0 | 1 | Neutral |
| 1 | 140 | 5.253 | 0 | 0 | 0.58 | 1 | 0 | 0.667 | 0 | 1 | Neutral |
| 1 | 141 | 6.966 | 0 | 0 | 1.609 | 1 | 0 | 0.667 | 0 | 1 | Neutral |
| 1 | 142 | 5.114 | 0 | 0 | 0.983 | 1 | 0 | 0.667 | 0 | 1 | Neutral |
| 1 | 143 | 28.547 | 0 | 0 | 7.11 | 1 | 0 | 0.667 | 0 | 1 | Neutral |
| 1 | 144 | 0 | 0 | 1 | 0 | 0 | 0 | 1 | 0 | 1 | Invariable |
| 1 | 145 | 18.882 | 0 | 0 | 2.624 | 1 | 0 | 0.667 | 0 | 1 | Neutral |
| 1 | 146 | 9.397 | 0 | 0 | 1.989 | 1 | 0 | 0.667 | 0 | 1 | Neutral |
| 1 | 147 | 9.564 | 0 | 0 | 1.894 | 1 | 0 | 0.667 | 0 | 1 | Neutral |
| 1 | 148 | 9.385 | 0 | 0 | 100.246 | 1 | 2.278 | 0.157 | 2 | 1 | Neutral |
| 1 | 149 | 0 | 0 | 1 | 0 | 0 | 0 | 1 | 0 | 1 | Invariable |
| 1 | 150 | 4.815 | 0 | 0 | 1.867 | 1 | 0 | 0.667 | 0 | 1 | Neutral |
| 1 | 151 | 18.158 | 0 | 0 | 2.792 | 1 | 0 | 0.667 | 0 | 1 | Neutral |
| 1 | 152 | 3.556 | 0 | 0 | 0.682 | 1 | 0 | 0.667 | 0 | 1 | Neutral |
| 1 | 153 | 0 | 0 | 1 | 0 | 0 | 0 | 1 | 0 | 1 | Invariable |
| 1 | 154 | 3.283 | 0 | 0 | 0.761 | 1 | 0 | 0.667 | 0 | 1 | Neutral |
| 1 | 155 | 15.001 | 0 | 0 | 4.605 | 1 | 0 | 0.667 | 0 | 1 | Neutral |
| 1 | 156 | 9.935 | 0 | 0 | 1.917 | 1 | 0 | 0.667 | 0 | 1 | Neutral |
| 1 | 157 | 0 | 0 | 1 | 0 | 0 | 0 | 1 | 0 | 1 | Invariable |
| 1 | 158 | 0 | 0 | 1 | 0 | 0 | 0 | 1 | 0 | 1 | Invariable |
| 1 | 159 | 3.655 | 0 | 0 | 0.749 | 1 | 0 | 0.667 | 0 | 1 | Neutral |
| 1 | 160 | 3.059 | 0 | 0 | 0.272 | 1 | 0 | 0.667 | 0 | 1 | Neutral |
| 1 | 161 | 10.709 | 0 | 0 | 1.947 | 1 | 0 | 0.667 | 0 | 1 | Neutral |
| 1 | 162 | 1.726 | 0 | 0 | 0.188 | 1 | 0 | 0.667 | 0 | 1 | Neutral |
| 1 | 163 | 29.259 | 0 | 0 | 3.636 | 1 | 0 | 0.667 | 0 | 1 | Neutral |
| 1 | 164 | 0 | 0 | 1 | 0 | 0 | 0 | 1 | 0 | 1 | Invariable |
| 1 | 165 | 2.626 | 0 | 0 | 21.571 | 1 | 0.759 | 0.366 | 1 | 1 | Neutral |
| 1 | 166 | 9.048 | 0 | 0 | 1.38 | 1 | 0 | 0.667 | 0 | 1 | Neutral |
| 1 | 167 | 6.064 | 0 | 0 | 1.607 | 1 | 0 | 0.667 | 0 | 1 | Neutral |
| 1 | 168 | 14.87 | 0 | 0 | 2.987 | 1 | 0 | 0.667 | 0 | 1 | Neutral |
| 1 | 169 | 0 | 0 | 1 | 0 | 0 | 0 | 1 | 0 | 1 | Invariable |
| 1 | 170 | 6.643 | 0 | 0 | 1.826 | 1 | 0 | 0.667 | 0 | 1 | Neutral |
| 1 | 171 | 12.292 | 0 | 0 | 2.03 | 1 | 0 | 0.667 | 0 | 1 | Neutral |
| 1 | 172 | 5.712 | 0 | 0 | 1.387 | 1 | 0 | 0.667 | 0 | 1 | Neutral |
| 1 | 173 | 8.708 | 0 | 0 | 1.558 | 1 | 0 | 0.667 | 0 | 1 | Neutral |
| 1 | 174 | 11.558 | 0 | 0 | 1.813 | 1 | 0 | 0.667 | 0 | 1 | Neutral |
| 1 | 175 | 12.574 | 0 | 0 | 2.059 | 1 | 0 | 0.667 | 0 | 1 | Neutral |
| 1 | 176 | 4.183 | 0 | 0 | 0.682 | 1 | 0 | 0.667 | 0 | 1 | Neutral |
| 1 | 177 | 21.094 | 0 | 0 | 3.879 | 1 | 0 | 0.667 | 0 | 1 | Neutral |
| 1 | 178 | 7.095 | 0 | 0 | 1.399 | 1 | 0 | 0.667 | 0 | 1 | Neutral |
| 1 | 179 | 0 | 0 | 1 | 0 | 0 | 0 | 1 | 0 | 1 | Invariable |
| 1 | 180 | 8.146 | 0 | 0 | 1.543 | 1 | 0 | 0.667 | 0 | 1 | Neutral |
| 1 | 181 | 2.88 | 0 | 0 | 0.678 | 1 | 0 | 0.667 | 0 | 1 | Neutral |
| 1 | 182 | 2.423 | 0 | 0 | 0.416 | 1 | 0 | 0.667 | 0 | 1 | Neutral |
| 1 | 183 | 3.655 | 0 | 0 | 0.749 | 1 | 0 | 0.667 | 0 | 1 | Neutral |
| 1 | 184 | 5.886 | 0 | 0 | 1.218 | 1 | 0 | 0.667 | 0 | 1 | Neutral |
| 1 | 185 | 1.606 | 0 | 0 | 0.274 | 1 | 0 | 0.667 | 0 | 1 | Neutral |
| 1 | 186 | 7.025 | 0 | 0 | 1.619 | 1 | 0 | 0.667 | 0 | 1 | Neutral |
| 1 | 187 | 10.486 | 0 | 0 | 1.832 | 1 | 0 | 0.667 | 0 | 1 | Neutral |
| 1 | 188 | 0 | 0 | 1 | 0 | 0 | 0 | 1 | 0 | 1 | Invariable |
| 1 | 189 | 8.716 | 0.765 | 0 | 0.942 | 1 | 0 | 0.667 | 0 | 1 | Neutral |
| 1 | 190 | 9.109 | 0 | 0 | 1.832 | 1 | 0 | 0.667 | 0 | 1 | Neutral |
| 1 | 191 | 10.15 | 0.819 | 0 | 1.63 | 1 | 0 | 0.667 | 0 | 1 | Neutral |
| 1 | 192 | 2.628 | 0 | 0 | 0.449 | 1 | 0 | 0.667 | 0 | 1 | Neutral |
| 1 | 193 | 5.951 | 0 | 0 | 9.537 | 1 | 0.049 | 0.605 | 1 | 1 | Neutral |
| 1 | 194 | 12.577 | 0 | 0 | 6.556 | 1 | 0 | 0.667 | 0 | 1 | Neutral |
| 1 | 195 | 5.089 | 0 | 0 | 0.98 | 1 | 0 | 0.667 | 0 | 1 | Neutral |
| 1 | 196 | 0 | 0 | 1 | 0 | 0 | 0 | 1 | 0 | 1 | Invariable |
| 1 | 197 | 50.532 | 0 | 0 | 3.204 | 1 | 0 | 0.667 | 0 | 1 | Neutral |
| 1 | 198 | 4.382 | 0 | 0 | 0.811 | 1 | 0 | 0.667 | 0 | 1 | Neutral |
| 1 | 199 | 3.285 | 0 | 0 | 0.176 | 1 | 0 | 0.667 | 0 | 1 | Neutral |
| 1 | 200 | 12.131 | 0 | 0 | 4.007 | 1 | 0 | 0.667 | 0 | 1 | Neutral |
| 1 | 201 | 0 | 0 | 1 | 0 | 0 | 0 | 1 | 0 | 1 | Invariable |
| 1 | 202 | 13.203 | 0 | 0 | 2.338 | 1 | 0 | 0.667 | 0 | 1 | Neutral |
| 1 | 203 | 6.677 | 0 | 0 | 1.109 | 1 | 0 | 0.667 | 0 | 1 | Neutral |
| 1 | 204 | 7.891 | 0 | 0 | 28.023 | 1 | 0.355 | 0.472 | 1 | 1 | Neutral |
| 1 | 205 | 6.457 | 0 | 0 | 1.103 | 1 | 0 | 0.667 | 0 | 1 | Neutral |
| 1 | 206 | 5.537 | 0 | 0 | 1.018 | 1 | 0 | 0.667 | 0 | 1 | Neutral |
| 1 | 207 | 9.249 | 0 | 0 | 1.967 | 1 | 0 | 0.667 | 0 | 1 | Neutral |
| 1 | 208 | 7.834 | 0 | 0 | 1.501 | 1 | 0 | 0.667 | 0 | 1 | Neutral |
| 1 | 209 | 9.69 | 0 | 0 | 2.031 | 1 | 0 | 0.667 | 0 | 1 | Neutral |
| 1 | 210 | 2.822 | 0 | 0 | 0.462 | 1 | 0 | 0.667 | 0 | 1 | Neutral |
| 1 | 211 | 7.566 | 0 | 0 | 1.709 | 1 | 0 | 0.667 | 0 | 1 | Neutral |
| 1 | 212 | 0 | 0 | 1 | 0 | 0 | 0 | 1 | 0 | 1 | Invariable |
| 1 | 213 | 14.581 | 0 | 0 | 49.996 | 1 | 0.326 | 0.482 | 1 | 1 | Neutral |
| 1 | 214 | 8.685 | 0 | 0 | 48.817 | 1 | 0.701 | 0.379 | 1 | 1 | Neutral |
| 1 | 215 | 15.401 | 0 | 0 | 2.736 | 1 | 0 | 0.667 | 0 | 1 | Neutral |
| 1 | 216 | 11.765 | 0 | 0 | 1.004 | 1 | 0 | 0.667 | 0 | 1 | Neutral |
| 1 | 217 | 21.791 | 1.843 | 0 | 2.246 | 1 | 0 | 0.667 | 0 | 1 | Neutral |
| 1 | 218 | 19.344 | 0 | 0 | 3.492 | 1 | 0 | 0.667 | 0 | 1 | Neutral |
| 1 | 219 | 14.609 | 1.998 | 0 | 6.541 | 1 | 0 | 0.667 | 0 | 1 | Neutral |
| 1 | 220 | 20.512 | 0 | 0 | 3.035 | 1 | 0 | 0.667 | 0 | 1 | Neutral |
| 1 | 221 | 5.444 | 0 | 0 | 1.259 | 1 | 0 | 0.667 | 0 | 1 | Neutral |
| 1 | 222 | 9.983 | 0 | 0 | 78.892 | 1 | 0.878 | 0.341 | 1 | 1 | Neutral |
| 1 | 223 | 12.557 | 0.901 | 0 | 1.024 | 1 | 0 | 0.667 | 0 | 1 | Neutral |
| 1 | 224 | 5.389 | 0 | 0 | 1.139 | 1 | 0 | 0.667 | 0 | 1 | Neutral |
| 1 | 225 | 7.834 | 0 | 0 | 1.501 | 1 | 0 | 0.667 | 0 | 1 | Neutral |
| 1 | 226 | 0 | 0 | 1 | 0 | 0 | 0 | 1 | 0 | 1 | Invariable |
| 1 | 227 | 11.828 | 0 | 0 | 1.425 | 1 | 0 | 0.667 | 0 | 1 | Neutral |
| 1 | 228 | 20.152 | 0 | 0 | 3.203 | 1 | 0 | 0.667 | 0 | 1 | Neutral |
| 1 | 229 | 15.965 | 0 | 0 | 2.666 | 1 | 0 | 0.667 | 0 | 1 | Neutral |
| 1 | 230 | 0 | 0 | 1 | 0 | 0 | 0 | 1 | 0 | 1 | Invariable |
| 1 | 231 | 2.836 | 0 | 0 | 0.15 | 1 | 0 | 0.667 | 0 | 1 | Neutral |
| 1 | 232 | 2.717 | 0 | 0 | 0.644 | 1 | 0 | 0.667 | 0 | 1 | Neutral |
| 1 | 233 | 0 | 0 | 1 | 0 | 0 | 0 | 1 | 0 | 1 | Invariable |
| 1 | 234 | 31.638 | 0 | 0 | 6.5 | 1 | 0 | 0.667 | 0 | 1 | Neutral |
| 1 | 235 | 7.081 | 0 | 0 | 1.628 | 1 | 0 | 0.667 | 0 | 1 | Neutral |
| 1 | 236 | 13.5 | 0 | 0 | 4.312 | 1 | 0 | 0.667 | 0 | 1 | Neutral |
| 1 | 237 | 7.582 | 0 | 0 | 1.896 | 1 | 0 | 0.667 | 0 | 1 | Neutral |
| 1 | 238 | 2.822 | 0 | 0 | 0.462 | 1 | 0 | 0.667 | 0 | 1 | Neutral |
| 1 | 239 | 0 | 0 | 1 | 0 | 0 | 0 | 1 | 0 | 1 | Invariable |
| 1 | 240 | 6.726 | 0 | 0 | 1.345 | 1 | 0 | 0.667 | 0 | 1 | Neutral |
| 1 | 241 | 4.573 | 0 | 0 | 0.811 | 1 | 0 | 0.667 | 0 | 1 | Neutral |
| 1 | 242 | 7.456 | 1.167 | 0 | 1.168 | 1 | 0 | 0.667 | 0 | 1 | Neutral |
| 1 | 243 | 5.629 | 0 | 0 | 1.178 | 1 | 0 | 0.667 | 0 | 1 | Neutral |
| 1 | 244 | 39.829 | 0 | 0 | 5.421 | 1 | 0 | 0.667 | 0 | 1 | Neutral |
| 1 | 245 | 7.081 | 0 | 0 | 1.534 | 1 | 0 | 0.667 | 0 | 1 | Neutral |
| 1 | 246 | 5.367 | 0 | 0 | 1.322 | 1 | 0 | 0.667 | 0 | 1 | Neutral |
| 1 | 247 | 22.97 | 0.85 | 0 | 3.611 | 1 | 0 | 0.667 | 0 | 1 | Neutral |
| 1 | 248 | 16.329 | 0 | 0 | 2.834 | 1 | 0 | 0.667 | 0 | 1 | Neutral |
| 1 | 249 | 17.205 | 0 | 0 | 2.923 | 1 | 0 | 0.667 | 0 | 1 | Neutral |
| 1 | 250 | 0 | 0 | 1 | 0 | 0 | 0 | 1 | 0 | 1 | Invariable |
| 1 | 251 | 2.698 | 0 | 0 | 0.766 | 1 | 0 | 0.667 | 0 | 1 | Neutral |
| 1 | 252 | 1.782 | 0 | 0 | 0.308 | 1 | 0 | 0.667 | 0 | 1 | Neutral |
| 1 | 253 | 18.579 | 0 | 0 | 2.6 | 1 | 0 | 0.667 | 0 | 1 | Neutral |
| 1 | 254 | 2.493 | 0 | 0 | 0.428 | 1 | 0 | 0.667 | 0 | 1 | Neutral |
| 1 | 255 | 5.317 | 0 | 0 | 0.945 | 1 | 0 | 0.667 | 0 | 1 | Neutral |
| 1 | 256 | 0 | 0 | 1 | 0 | 0 | 0 | 1 | 0 | 1 | Invariable |
| 1 | 257 | 13.437 | 0 | 0 | 3.74 | 1 | 0 | 0.667 | 0 | 1 | Neutral |
| 1 | 258 | 0 | 0 | 1 | 0 | 0 | 0 | 1 | 0 | 1 | Invariable |
| 1 | 259 | 13.649 | 0 | 0 | 4.323 | 1 | 0 | 0.667 | 0 | 1 | Neutral |
| 1 | 260 | 41.472 | 0 | 0 | 2.231 | 1 | 0 | 0.667 | 0 | 1 | Neutral |
| 1 | 261 | 18.972 | 9.656 | 0 | 14.066 | 1 | 0 | 0.667 | 0 | 1 | Neutral |
| 1 | 262 | 4.615 | 0 | 0 | 0.907 | 1 | 0 | 0.667 | 0 | 1 | Neutral |
| 1 | 263 | 13.285 | 0 | 0 | 3.752 | 1 | 0 | 0.667 | 0 | 1 | Neutral |
| 1 | 264 | 8.895 | 0 | 0 | 1.64 | 1 | 0 | 0.667 | 0 | 1 | Neutral |
| 1 | 265 | 6.182 | 0 | 0 | 1.264 | 1 | 0 | 0.667 | 0 | 1 | Neutral |
| 1 | 266 | 14.705 | 0 | 0 | 2.941 | 1 | 0 | 0.667 | 0 | 1 | Neutral |
| 1 | 267 | 4.185 | 0 | 0 | 104.288 | 1 | 1.72 | 0.212 | 1 | 1 | Neutral |
| 1 | 268 | 8.708 | 0 | 0 | 1.558 | 1 | 0 | 0.667 | 0 | 1 | Neutral |
| 1 | 269 | 3.691 | 1.266 | 0 | 1.171 | 1 | 0 | 0.667 | 0 | 1 | Neutral |
| 1 | 270 | 5.559 | 0 | 0 | 1.359 | 1 | 0 | 0.667 | 0 | 1 | Neutral |
| 1 | 271 | 0 | 0 | 1 | 0 | 0 | 0 | 1 | 0 | 1 | Invariable |
| **1** | **272** | **17.487** | **0** | **0** | **1,00,000** | **1** | **12.603** | **0.001** | **1** | **0.418** | **Diversifying** |
| 1 | 273 | 8.507 | 0 | 0 | 2.677 | 1 | 0 | 0.667 | 0 | 1 | Neutral |
| 1 | 274 | 131.279 | 0 | 0 | 6.034 | 1 | 0 | 0.667 | 0 | 1 | Neutral |
| 1 | 275 | 20.152 | 0 | 0 | 3.203 | 1 | 0 | 0.667 | 0 | 1 | Neutral |
| 1 | 276 | 9.723 | 0 | 0 | 2.087 | 1 | 0 | 0.667 | 0 | 1 | Neutral |
| 1 | 277 | 13.286 | 0 | 0 | 2.652 | 1 | 0 | 0.667 | 0 | 1 | Neutral |
| 1 | 278 | 2.182 | 0 | 0 | 0.377 | 1 | 0 | 0.667 | 0 | 1 | Neutral |
| 1 | 279 | 11.819 | 0 | 0 | 2.317 | 1 | 0 | 0.667 | 0 | 1 | Neutral |
| 1 | 280 | 1.885 | 0 | 0 | 0.32 | 1 | 0 | 0.667 | 0 | 1 | Neutral |
| 1 | 281 | 17.462 | 0 | 0 | 2.949 | 1 | 0 | 0.667 | 0 | 1 | Neutral |
| 1 | 282 | 38.2 | 0 | 0 | 5.427 | 1 | 0 | 0.667 | 0 | 1 | Neutral |
| 1 | 283 | 20.588 | 0 | 0 | 4.401 | 1 | 0 | 0.667 | 0 | 1 | Neutral |
| 1 | 284 | 4.016 | 0 | 0 | 1.11 | 1 | 0 | 0.667 | 0 | 1 | Neutral |
| 1 | 285 | 11.068 | 0 | 0 | 60.926 | 1 | 0.635 | 0.395 | 1 | 1 | Neutral |
| 1 | 286 | 12.806 | 0 | 0 | 2.859 | 1 | 0 | 0.667 | 0 | 1 | Neutral |
| 1 | 287 | 9.561 | 0 | 0 | 2.4 | 1 | 0 | 0.667 | 0 | 1 | Neutral |
| 1 | 288 | 0 | 0 | 1 | 0 | 0 | 0 | 1 | 0 | 1 | Invariable |
| 1 | 289 | 21.78 | 0 | 0 | 6.118 | 1 | 0 | 0.667 | 0 | 1 | Neutral |
| 1 | 290 | 0 | 0 | 1 | 0 | 0 | 0 | 1 | 0 | 1 | Invariable |
| 1 | 291 | 6.544 | 0 | 0 | 1.314 | 1 | 0 | 0.667 | 0 | 1 | Neutral |
| 1 | 292 | 3.988 | 0 | 0 | 0.411 | 1 | 0 | 0.667 | 0 | 1 | Neutral |
| 1 | 293 | 5.992 | 0 | 0 | 1.235 | 1 | 0 | 0.667 | 0 | 1 | Neutral |
| 1 | 294 | 5.444 | 0 | 0 | 1.259 | 1 | 0 | 0.667 | 0 | 1 | Neutral |
| 1 | 295 | 1.849 | 0 | 0 | 0.142 | 1 | 0 | 0.667 | 0 | 1 | Neutral |
| 1 | 296 | 13.64 | 0 | 0 | 2.414 | 1 | 0 | 0.667 | 0 | 1 | Neutral |
| 1 | 297 | 16.607 | 0 | 0 | 2.863 | 1 | 0 | 0.667 | 0 | 1 | Neutral |
| 1 | 298 | 3.467 | 0 | 0 | 0.186 | 1 | 0 | 0.667 | 0 | 1 | Neutral |
| 1 | 299 | 3.508 | 0 | 0 | 0.805 | 1 | 0 | 0.667 | 0 | 1 | Neutral |
| 1 | 300 | 6.088 | 0 | 0 | 1.25 | 1 | 0 | 0.667 | 0 | 1 | Neutral |
| 1 | 301 | 1.522 | 0 | 0 | 0.156 | 1 | 0 | 0.667 | 0 | 1 | Neutral |
| 1 | 302 | 10.611 | 1.025 | 0 | 1.114 | 1 | 0 | 0.667 | 0 | 1 | Neutral |
| 1 | 303 | 12.868 | 0 | 0 | 2.457 | 1 | 0 | 0.667 | 0 | 1 | Neutral |
| 1 | 304 | 7.459 | 0 | 0 | 1.46 | 1 | 0 | 0.667 | 0 | 1 | Neutral |
| 1 | 305 | 13.069 | 0 | 0 | 2.109 | 1 | 0 | 0.667 | 0 | 1 | Neutral |
| 1 | 306 | 11.441 | 0 | 0 | 1.939 | 1 | 0 | 0.667 | 0 | 1 | Neutral |
| 1 | 307 | 11.136 | 0 | 0 | 1.905 | 1 | 0 | 0.667 | 0 | 1 | Neutral |
| 1 | 308 | 5.629 | 0 | 0 | 1.178 | 1 | 0 | 0.667 | 0 | 1 | Neutral |
| 1 | 309 | 18.353 | 0 | 0 | 3.386 | 1 | 0 | 0.667 | 0 | 1 | Neutral |
| 1 | 310 | 40.381 | 0 | 0 | 1.734 | 1 | 0 | 0.667 | 0 | 1 | Neutral |
| 1 | 311 | 6.774 | 0 | 0 | 1.835 | 1 | 0 | 0.667 | 0 | 1 | Neutral |
| 1 | 312 | 0 | 0 | 1 | 0 | 0 | 0 | 1 | 0 | 1 | Invariable |
| 1 | 313 | 8.362 | 0.886 | 0 | 0.869 | 1 | 0 | 0.667 | 0 | 1 | Neutral |
| 1 | 314 | 32.855 | 0 | 0 | 4.577 | 1 | 0 | 0.667 | 0 | 1 | Neutral |
| 1 | 315 | 7.596 | 0 | 0 | 1.713 | 1 | 0 | 0.667 | 0 | 1 | Neutral |
| 1 | 316 | 0 | 0 | 0 | 0.799 | 1 | 0.496 | 0.43 | 1 | 1 | Neutral |
| 1 | 317 | 32.108 | 0 | 0 | 10.326 | 1 | 0 | 0.667 | 0 | 1 | Neutral |
| 1 | 318 | 0 | 0 | 1 | 0 | 0 | 0 | 1 | 0 | 1 | Invariable |
| 1 | 319 | 14.979 | 0 | 0 | 2.293 | 1 | 0 | 0.667 | 0 | 1 | Neutral |
| 1 | 320 | 15.763 | 0 | 0 | 2.775 | 1 | 0 | 0.667 | 0 | 1 | Neutral |
| 1 | 321 | 8.178 | 0 | 0 | 1.789 | 1 | 0 | 0.667 | 0 | 1 | Neutral |
| 1 | 322 | 2.188 | 0 | 0 | 0.378 | 1 | 0 | 0.667 | 0 | 1 | Neutral |
| 1 | 323 | 17.285 | 0 | 0 | 2.753 | 1 | 0 | 0.667 | 0 | 1 | Neutral |
| 1 | 324 | 4.411 | 0 | 0 | 1.46 | 1 | 0 | 0.667 | 0 | 1 | Neutral |
| 1 | 325 | 0 | 0 | 1 | 0 | 0 | 0 | 1 | 0 | 1 | Invariable |
| 1 | 326 | 0 | 0 | 1 | 0 | 0 | 0 | 1 | 0 | 1 | Invariable |
| 1 | 327 | 21.45 | 0 | 0 | 3.939 | 1 | 0 | 0.667 | 0 | 1 | Neutral |
| 1 | 328 | 35.692 | 0 | 0 | 6.673 | 1 | 0 | 0.667 | 0 | 1 | Neutral |
| 1 | 329 | 2.673 | 0 | 0 | 0.554 | 1 | 0 | 0.667 | 0 | 1 | Neutral |
| 1 | 330 | 7.081 | 0 | 0 | 1.534 | 1 | 0 | 0.667 | 0 | 1 | Neutral |
| 1 | 331 | 14.48 | 0 | 0 | 2.246 | 1 | 0 | 0.667 | 0 | 1 | Neutral |
| 1 | 332 | 4.615 | 0 | 0 | 0.907 | 1 | 0 | 0.667 | 0 | 1 | Neutral |
| 1 | 333 | 2.221 | 0 | 0 | 0.383 | 1 | 0 | 0.667 | 0 | 1 | Neutral |
| 1 | 334 | 14.516 | 0 | 0 | 3.165 | 1 | 0 | 0.667 | 0 | 1 | Neutral |
| 1 | 335 | 2.324 | 0 | 0 | 0.4 | 1 | 0 | 0.667 | 0 | 1 | Neutral |
| 1 | 336 | 8.708 | 0 | 0 | 1.558 | 1 | 0 | 0.667 | 0 | 1 | Neutral |
| 1 | 337 | 2.206 | 0 | 0 | 44.459 | 1 | 1.432 | 0.249 | 1 | 1 | Neutral |
| 1 | 338 | 1.997 | 0 | 0 | 0.329 | 1 | 0 | 0.667 | 0 | 1 | Neutral |
| 1 | 339 | 10.208 | 0 | 0 | 1.8 | 1 | 0 | 0.667 | 0 | 1 | Neutral |
| 1 | 340 | 57.173 | 0 | 0 | 14.583 | 1 | 0 | 0.667 | 0 | 1 | Neutral |
| 1 | 341 | 37.139 | 0 | 0 | 9.156 | 1 | 0 | 0.667 | 0 | 1 | Neutral |
| 1 | 342 | 17.067 | 0 | 0 | 2.909 | 1 | 0 | 0.667 | 0 | 1 | Neutral |
| 1 | 343 | 0 | 0 | 1 | 0 | 0 | 0 | 1 | 0 | 1 | Invariable |
| 1 | 344 | 2.509 | 0 | 0 | 0.268 | 1 | 0 | 0.667 | 0 | 1 | Neutral |
| 1 | 345 | 7.236 | 0 | 0 | 1.753 | 1 | 0 | 0.667 | 0 | 1 | Neutral |
| 1 | 346 | 3.098 | 0 | 0 | 0.723 | 1 | 0 | 0.667 | 0 | 1 | Neutral |
| 1 | 347 | 7.046 | 0 | 0 | 1.217 | 1 | 0 | 0.667 | 0 | 1 | Neutral |
| 1 | 348 | 10.554 | 0 | 0 | 2.151 | 1 | 0 | 0.667 | 0 | 1 | Neutral |
| 1 | 349 | 62.217 | 0 | 0 | 2.279 | 1 | 0 | 0.667 | 0 | 1 | Neutral |
| 1 | 350 | 16.322 | 0 | 0 | 4.316 | 1 | 0 | 0.667 | 0 | 1 | Neutral |
| 1 | 351 | 6.995 | 0 | 0 | 1.123 | 1 | 0 | 0.667 | 0 | 1 | Neutral |
| 1 | 352 | 7.124 | 0 | 0 | 1.403 | 1 | 0 | 0.667 | 0 | 1 | Neutral |
| 1 | 353 | 17.285 | 0 | 0 | 2.753 | 1 | 0 | 0.667 | 0 | 1 | Neutral |
| 1 | 354 | 8.554 | 0 | 0 | 1.597 | 1 | 0 | 0.667 | 0 | 1 | Neutral |
| 1 | 355 | 15.297 | 0 | 0 | 4.688 | 1 | 0 | 0.667 | 0 | 1 | Neutral |
| 1 | 356 | 12.083 | 0 | 0 | 2.179 | 1 | 0 | 0.667 | 0 | 1 | Neutral |
| 1 | 357 | 0 | 0 | 1 | 0 | 0 | 0 | 1 | 0 | 1 | Invariable |
| 1 | 358 | 0 | 0 | 1 | 0 | 0 | 0 | 1 | 0 | 1 | Invariable |
| 1 | 359 | 6.93 | 0 | 0 | 1.153 | 1 | 0 | 0.667 | 0 | 1 | Neutral |
| 1 | 360 | 0 | 0 | 1 | 0 | 0 | 0 | 1 | 0 | 1 | Invariable |
| 1 | 361 | 1.896 | 0 | 0 | 0.312 | 1 | 0 | 0.667 | 0 | 1 | Neutral |
| 1 | 362 | 2.6 | 0 | 0 | 0.45 | 1 | 0 | 0.667 | 0 | 1 | Neutral |
| 1 | 363 | 5.411 | 0 | 0 | 51.108 | 1 | 0.997 | 0.318 | 1 | 1 | Neutral |
| 1 | 364 | 8.133 | 0 | 0 | 3.276 | 1 | 0 | 0.667 | 0 | 1 | Neutral |
| 1 | 365 | 9.787 | 0 | 0 | 19.434 | 1 | 0.127 | 0.56 | 1 | 1 | Neutral |
| 1 | 366 | 4.793 | 0 | 0 | 1.021 | 1 | 0 | 0.667 | 0 | 1 | Neutral |
| 1 | 367 | 4.362 | 1.071 | 0 | 0.801 | 1 | 0 | 0.667 | 0 | 1 | Neutral |
| 1 | 368 | 3.003 | 0 | 0 | 0.704 | 1 | 0 | 0.667 | 0 | 1 | Neutral |
| 1 | 369 | 5.632 | 0 | 0 | 0.996 | 1 | 0 | 0.667 | 0 | 1 | Neutral |
| 1 | 370 | 8.708 | 0 | 0 | 1.558 | 1 | 0 | 0.667 | 0 | 1 | Neutral |
| 1 | 371 | 7.369 | 0 | 0 | 1.433 | 1 | 0 | 0.667 | 0 | 1 | Neutral |
| 1 | 372 | 8.489 | 0 | 0 | 1.771 | 1 | 0 | 0.667 | 0 | 1 | Neutral |
| 1 | 373 | 17.706 | 0 | 0 | 5.157 | 1 | 0 | 0.667 | 0 | 1 | Neutral |
| 1 | 374 | 2.934 | 1.074 | 0 | 0.982 | 1 | 0 | 0.667 | 0 | 1 | Neutral |
| 1 | 375 | 8.999 | 0 | 0 | 1.384 | 1 | 0 | 0.667 | 0 | 1 | Neutral |
| 1 | 376 | 4.87 | 0 | 0 | 0.571 | 1 | 0 | 0.667 | 0 | 1 | Neutral |
| 1 | 377 | 10.078 | 0 | 0 | 1.784 | 1 | 0 | 0.667 | 0 | 1 | Neutral |
| 1 | 378 | 16.738 | 0.916 | 0 | 3.583 | 1 | 0 | 0.667 | 0 | 1 | Neutral |
| 1 | 379 | 3.819 | 0 | 0 | 4.016 | 1 | -0.004 | 0.667 | 1 | 1 | Neutral |
| 1 | 380 | 19.238 | 0.815 | 0 | 1.863 | 1 | 0 | 0.667 | 0 | 1 | Neutral |
| 1 | 381 | 4.157 | 0 | 0 | 0.777 | 1 | 0 | 0.667 | 0 | 1 | Neutral |
| 1 | 382 | 0 | 0 | 1 | 0 | 0 | 0 | 1 | 0 | 1 | Invariable |
| 1 | 383 | 2.004 | 0.792 | 0 | 1.269 | 1 | 0 | 0.667 | 0 | 1 | Neutral |
| 1 | 384 | 4.638 | 0 | 0 | 0.584 | 1 | 0 | 0.667 | 0 | 1 | Neutral |
| 1 | 385 | 0 | 0 | 1 | 0 | 0 | 0 | 1 | 0 | 1 | Invariable |
| 1 | 386 | 0 | 0 | 1 | 0 | 0 | 0 | 1 | 0 | 1 | Invariable |
| 1 | 387 | 0 | 0 | 1 | 0 | 0 | 0 | 1 | 0 | 1 | Invariable |
| 1 | 388 | 19.845 | 0 | 0 | 2.698 | 1 | 0 | 0.667 | 0 | 1 | Neutral |
| 1 | 389 | 1.885 | 0 | 0 | 0.32 | 1 | 0 | 0.667 | 0 | 1 | Neutral |
| 1 | 390 | 4.902 | 0 | 0 | 0.983 | 1 | 0 | 0.667 | 0 | 1 | Neutral |
| 1 | 391 | 0 | 0 | 1 | 0 | 0 | 0 | 1 | 0 | 1 | Invariable |
| 1 | 392 | 33.253 | 0 | 0 | 7.154 | 1 | 0 | 0.667 | 0 | 1 | Neutral |
| 1 | 393 | 5.632 | 0 | 0 | 0.988 | 1 | 0 | 0.667 | 0 | 1 | Neutral |
| 1 | 394 | 9.653 | 0 | 0 | 3.538 | 1 | 0 | 0.667 | 0 | 0.998 | Neutral |
| 1 | 395 | 10.263 | 0 | 0 | 1.8 | 1 | 0 | 0.667 | 0 | 0.995 | Neutral |
| 1 | 396 | 16.659 | 0 | 0 | 2.868 | 1 | 0 | 0.667 | 0 | 0.993 | Neutral |
| 1 | 397 | 2.064 | 0 | 0 | 0.357 | 1 | 0 | 0.667 | 0 | 0.99 | Neutral |
| 1 | 398 | 9.225 | 0 | 0 | 0.744 | 1 | 0 | 0.667 | 0 | 0.987 | Neutral |
| 1 | 399 | 14.688 | 0 | 0 | 4.666 | 1 | 0 | 0.667 | 0 | 0.984 | Neutral |
| 1 | 400 | 6.089 | 0 | 0 | 1.25 | 1 | 0 | 0.667 | 0 | 0.981 | Neutral |
| 1 | 401 | 13.616 | 0 | 0 | 2.536 | 1 | 0 | 0.667 | 0 | 0.979 | Neutral |
| 1 | 402 | 3.728 | 0 | 0 | 3.579 | 1 | 0 | 0.667 | 0 | 0.976 | Neutral |
| 1 | 403 | 19.891 | 1.476 | 0 | 24.754 | 1 | 0.015 | 0.635 | 0 | 1 | Neutral |
| 1 | 404 | 4.454 | 0 | 0 | 0.371 | 1 | 0 | 0.667 | 0 | 0.973 | Neutral |
| 1 | 405 | 16.679 | 0 | 0 | 2.87 | 1 | 0 | 0.667 | 0 | 0.971 | Neutral |
| 1 | 406 | 8.955 | 0 | 0 | 1.838 | 1 | 0 | 0.667 | 0 | 0.968 | Neutral |
| 1 | 407 | 3.655 | 0 | 0 | 0.749 | 1 | 0 | 0.667 | 0 | 0.965 | Neutral |
| 1 | 408 | 1.896 | 0 | 0 | 0.312 | 1 | 0 | 0.667 | 0 | 0.963 | Neutral |
| 1 | 409 | 0 | 0 | 1 | 0 | 0 | 0 | 1 | 0 | 1 | Invariable |
| 1 | 410 | 0 | 0 | 1 | 0 | 0 | 0 | 1 | 0 | 1 | Invariable |
| 1 | 411 | 2.74 | 0 | 0 | 0.554 | 1 | 0 | 0.667 | 0 | 0.96 | Neutral |
| 1 | 412 | 1.965 | 0 | 0 | 0.34 | 1 | 0 | 0.667 | 0 | 0.958 | Neutral |
| 1 | 413 | 12.276 | 0 | 0 | 4.04 | 1 | 0 | 0.667 | 0 | 0.955 | Neutral |
| 1 | 414 | 0 | 0 | 1 | 0 | 0 | 0 | 1 | 0 | 1 | Invariable |
| 1 | 415 | 15.359 | 0 | 0 | 2.731 | 1 | 0 | 0.667 | 0 | 0.952 | Neutral |
| 1 | 416 | 0 | 0 | 1 | 0 | 0 | 0 | 1 | 0 | 1 | Invariable |
| 1 | 417 | 29.398 | 0 | 0 | 9.924 | 1 | 0 | 0.667 | 0 | 0.95 | Neutral |
| 1 | 418 | 16.862 | 0 | 0 | 3.466 | 1 | 0 | 0.667 | 0 | 0.947 | Neutral |
| 1 | 419 | 22.667 | 0 | 0 | 2.901 | 1 | 0 | 0.667 | 0 | 0.945 | Neutral |
| 1 | 420 | 4.793 | 0 | 0 | 1.021 | 1 | 0 | 0.667 | 0 | 0.942 | Neutral |
| 1 | 421 | 9.059 | 0 | 0 | 3.254 | 1 | 0 | 0.667 | 0 | 0.94 | Neutral |
| 1 | 422 | 3.283 | 0 | 0 | 0.761 | 1 | 0 | 0.667 | 0 | 0.937 | Neutral |
| 1 | 423 | 19.34 | 0 | 0 | 2.659 | 1 | 0 | 0.667 | 0 | 0.935 | Neutral |
| **1** | **424** | **14.757** | **0** | **0** | **328.144** | **1** | **3.85** | **0.068** | **2** | **1** | **Diversifying** |
| 1 | 425 | 20.796 | 0 | 0 | 3.403 | 1 | 0 | 0.667 | 0 | 0.932 | Neutral |
| 1 | 426 | 20.649 | 0 | 0 | 2.758 | 1 | 0 | 0.667 | 0 | 0.93 | Neutral |
| 1 | 427 | 16.2 | 0 | 0 | 2.402 | 1 | 0 | 0.667 | 0 | 0.927 | Neutral |
| 1 | 428 | 3.283 | 0 | 0 | 0.761 | 1 | 0 | 0.667 | 0 | 0.925 | Neutral |
| 1 | 429 | 11.211 | 0 | 0 | 1.907 | 1 | 0 | 0.667 | 0 | 0.923 | Neutral |
| 1 | 430 | 17.371 | 0 | 0 | 4.731 | 1 | 0 | 0.667 | 0 | 0.92 | Neutral |
| 1 | 431 | 0 | 0 | 1 | 0 | 0 | 0 | 1 | 0 | 1 | Invariable |
| 1 | 432 | 43.525 | 0 | 0 | 1.917 | 1 | 0 | 0.667 | 0 | 0.918 | Neutral |
| 1 | 433 | 24.696 | 0 | 0 | 8.887 | 1 | 0 | 0.667 | 0 | 0.915 | Neutral |
| 1 | 434 | 7.381 | 0 | 0 | 1.206 | 1 | 0 | 0.667 | 0 | 0.913 | Neutral |
| 1 | 435 | 13.339 | 0 | 0 | 4.256 | 1 | 0 | 0.667 | 0 | 0.911 | Neutral |
| 1 | 436 | 2.588 | 0 | 0 | 0.136 | 1 | 0 | 0.667 | 0 | 0.908 | Neutral |
| 1 | 437 | 0 | 0 | 1 | 0 | 0 | 0 | 1 | 0 | 1 | Invariable |
| 1 | 438 | 4.115 | 0 | 0 | 0.916 | 1 | 0 | 0.667 | 0 | 0.906 | Neutral |
| 1 | 439 | 9.154 | 0 | 0 | 107.552 | 1 | 1.241 | 0.277 | 1 | 1 | Neutral |
| 1 | 440 | 17.369 | 0 | 0 | 5.093 | 1 | 0 | 0.667 | 0 | 0.904 | Neutral |
| 1 | 441 | 11.342 | 0 | 0 | 1.652 | 1 | 0 | 0.667 | 0 | 0.901 | Neutral |
| 1 | 442 | 10.198 | 0 | 0 | 1.799 | 1 | 0 | 0.667 | 0 | 0.899 | Neutral |
| 1 | 443 | 13.741 | 0 | 0 | 2.176 | 1 | 0 | 0.667 | 0 | 0.897 | Neutral |
| 1 | 444 | 34.832 | 0 | 0 | 4.434 | 1 | 0 | 0.667 | 0 | 0.895 | Neutral |
| 1 | 445 | 28.637 | 0 | 0 | 6.503 | 1 | 0 | 0.667 | 0 | 0.892 | Neutral |
| 1 | 446 | 5.317 | 0 | 0 | 0.945 | 1 | 0 | 0.667 | 0 | 0.89 | Neutral |
| 1 | 447 | 31.227 | 0 | 0 | 4.24 | 1 | 0 | 0.667 | 0 | 0.888 | Neutral |
| 1 | 448 | 2.093 | 0 | 0 | 0.344 | 1 | 0 | 0.667 | 0 | 0.886 | Neutral |
| 1 | 449 | 3.655 | 0 | 0 | 0.749 | 1 | 0 | 0.667 | 0 | 0.883 | Neutral |
| 1 | 450 | 40.646 | 0 | 0 | 1.927 | 1 | 0 | 0.667 | 0 | 0.881 | Neutral |
| 1 | 451 | 28.322 | 0 | 0 | 6.539 | 1 | 0 | 0.667 | 0 | 0.879 | Neutral |
| 1 | 452 | 12.928 | 0 | 0 | 2.095 | 1 | 0 | 0.667 | 0 | 0.877 | Neutral |
| 1 | 453 | 7.136 | 0 | 0 | 1.814 | 1 | 0 | 0.667 | 0 | 0.875 | Neutral |
| 1 | 454 | 5.314 | 0 | 0 | 0.569 | 1 | 0 | 0.667 | 0 | 0.872 | Neutral |
| 1 | 455 | 13.379 | 0 | 0 | 3.791 | 1 | 0 | 0.667 | 0 | 0.87 | Neutral |
| 1 | 456 | 15.982 | 0 | 0 | 3.142 | 1 | 0 | 0.667 | 0 | 0.868 | Neutral |
| 1 | 457 | 32.636 | 0 | 0 | 4.006 | 1 | 0 | 0.667 | 0 | 0.866 | Neutral |
| 1 | 458 | 4.902 | 0 | 0 | 0.983 | 1 | 0 | 0.667 | 0 | 0.864 | Neutral |
| 1 | 459 | 5.734 | 0 | 0 | 1.079 | 1 | 0 | 0.667 | 0 | 0.862 | Neutral |
| 1 | 460 | 42.625 | 0 | 0 | 4.322 | 1 | 0 | 0.667 | 0 | 0.86 | Neutral |
| 1 | 461 | 0 | 0 | 1 | 0 | 0 | 0 | 1 | 0 | 1 | Invariable |
| 1 | 462 | 28.698 | 0 | 0 | 2.247 | 1 | 0 | 0.667 | 0 | 0.858 | Neutral |
| 1 | 463 | 7.797 | 0 | 0 | 2.352 | 1 | 0 | 0.667 | 0 | 0.856 | Neutral |
| 1 | 464 | 23.016 | 0 | 0 | 3.343 | 1 | 0 | 0.667 | 0 | 0.853 | Neutral |
| 1 | 465 | 5.314 | 0 | 0 | 0.569 | 1 | 0 | 0.667 | 0 | 0.851 | Neutral |
| 1 | 466 | 0 | 0 | 1 | 0 | 0 | 0 | 1 | 0 | 1 | Invariable |
| 1 | 467 | 12.063 | 0 | 0 | 1.675 | 1 | 0 | 0.667 | 0 | 0.849 | Neutral |
| 1 | 468 | 10.172 | 0 | 0 | 2.476 | 1 | 0 | 0.667 | 0 | 0.847 | Neutral |
| 1 | 469 | 8.915 | 0 | 0 | 1.643 | 1 | 0 | 0.667 | 0 | 0.845 | Neutral |
| 1 | 470 | 0 | 0 | 1 | 0 | 0 | 0 | 1 | 0 | 1 | Invariable |
| 1 | 471 | 8.489 | 0 | 0 | 1.771 | 1 | 0 | 0.667 | 0 | 0.843 | Neutral |
| 1 | 472 | 18.889 | 0 | 0 | 3.638 | 1 | 0 | 0.667 | 0 | 0.841 | Neutral |
| 1 | 473 | 14.239 | 0 | 0 | 2.045 | 1 | 0 | 0.667 | 0 | 0.839 | Neutral |
| 1 | 474 | 17.806 | 0 | 0 | 2.995 | 1 | 0 | 0.667 | 0 | 0.837 | Neutral |
| 1 | 475 | 2.88 | 0 | 0 | 0.678 | 1 | 0 | 0.667 | 0 | 0.835 | Neutral |
| 1 | 476 | 2.064 | 0 | 0 | 0.357 | 1 | 0 | 0.667 | 0 | 0.833 | Neutral |
| 1 | 477 | 6.533 | 0 | 0 | 39.064 | 1 | 0.712 | 0.376 | 1 | 1 | Neutral |
| 1 | 478 | 2.206 | 0 | 0 | 0.363 | 1 | 0 | 0.667 | 0 | 0.831 | Neutral |
| 1 | 479 | 2.929 | 0 | 0 | 0.881 | 1 | 0 | 0.667 | 0 | 0.829 | Neutral |
| 1 | 480 | 8.031 | 0 | 0 | 1.813 | 1 | 0 | 0.667 | 0 | 0.827 | Neutral |
| 1 | 481 | 30.402 | 0 | 0 | 3.867 | 1 | 0 | 0.667 | 0 | 0.826 | Neutral |
| 1 | 482 | 49.276 | 0 | 0 | 9.168 | 1 | 0 | 0.667 | 0 | 0.824 | Neutral |
| 1 | 483 | 9.571 | 0 | 0 | 2.855 | 1 | 0 | 0.667 | 0 | 0.822 | Neutral |
| 1 | 484 | 7.656 | 0 | 0 | 1.723 | 1 | 0 | 0.667 | 0 | 0.82 | Neutral |
| 1 | 485 | 1.774 | 0 | 0 | 0.291 | 1 | 0 | 0.667 | 0 | 0.818 | Neutral |
| 1 | 486 | 10.437 | 0 | 0 | 2.135 | 1 | 0 | 0.667 | 0 | 0.816 | Neutral |
| 1 | 487 | 17.746 | 0 | 0 | 2.533 | 1 | 0 | 0.667 | 0 | 0.814 | Neutral |
| 1 | 488 | 65.644 | 0 | 0 | 15.682 | 1 | 0 | 0.667 | 0 | 0.812 | Neutral |
| 1 | 489 | 18.171 | 0 | 0 | 5.215 | 1 | 0 | 0.667 | 0 | 0.81 | Neutral |
| 1 | 490 | 1.774 | 0 | 0 | 0.291 | 1 | 0 | 0.667 | 0 | 0.809 | Neutral |
| 1 | 491 | 5.412 | 0 | 0 | 1.254 | 1 | 0 | 0.667 | 0 | 0.807 | Neutral |
| 1 | 492 | 5.751 | 0 | 0 | 0.623 | 1 | 0 | 0.667 | 0 | 0.805 | Neutral |
| 1 | 493 | 6.677 | 0 | 0 | 1.109 | 1 | 0 | 0.667 | 0 | 0.803 | Neutral |
| 1 | 494 | 3.547 | 0 | 0 | 0.798 | 1 | 0 | 0.667 | 0 | 0.801 | Neutral |
| 1 | 495 | 5.606 | 0 | 0 | 1.367 | 1 | 0 | 0.667 | 0 | 0.799 | Neutral |
| 1 | 496 | 3.082 | 0 | 0 | 0.279 | 1 | 0 | 0.667 | 0 | 0.798 | Neutral |
| 1 | 497 | 5.614 | 0 | 0 | 1.507 | 1 | 0 | 0.667 | 0 | 0.796 | Neutral |
| 1 | 498 | 8.869 | 0 | 0 | 90.519 | 1 | 1.02 | 0.314 | 1 | 1 | Neutral |
| 1 | 499 | 15.436 | 1.677 | 0 | 1.7 | 1 | 0 | 0.667 | 0 | 0.794 | Neutral |
| 1 | 500 | 9.049 | 0.777 | 0 | 0.625 | 1 | 0 | 0.667 | 0 | 0.792 | Neutral |
| 1 | 501 | 3.111 | 0 | 0 | 0.529 | 1 | 0 | 0.667 | 0 | 0.79 | Neutral |
| 1 | 502 | 10.675 | 0 | 0 | 4.811 | 1 | 0 | 0.667 | 0 | 0.789 | Neutral |
| **1** | **503** | **3.752** | **0** | **0** | **973.394** | **1** | **3.575** | **0.079** | **2** | **1** | **Diversifying** |
| 1 | 504 | 11.251 | 1.729 | 0 | 1.829 | 1 | 0 | 0.667 | 0 | 0.787 | Neutral |
| 1 | 505 | 10.188 | 0 | 0 | 1.797 | 1 | 0 | 0.667 | 0 | 0.785 | Neutral |
| 1 | 506 | 0 | 0 | 1 | 0 | 0 | 0 | 1 | 0 | 1 | Invariable |
| 1 | 507 | 38.676 | 0 | 0 | 4.693 | 1 | 0 | 0.667 | 0 | 0.783 | Neutral |
| 1 | 508 | 14.098 | 0 | 0 | 2.21 | 1 | 0 | 0.667 | 0 | 0.782 | Neutral |
| 1 | 509 | 5.075 | 0 | 0 | 121.859 | 1 | 1.765 | 0.207 | 1 | 1 | Neutral |
| 1 | 510 | 5.269 | 0 | 0 | 1.116 | 1 | 0 | 0.667 | 0 | 0.78 | Neutral |
| 1 | 511 | 13.658 | 0 | 0 | 20.977 | 1 | 0.036 | 0.615 | 1 | 1 | Neutral |
| 1 | 512 | 5.403 | 0 | 0 | 1.141 | 1 | 0 | 0.667 | 0 | 0.778 | Neutral |
| 1 | 513 | 8.846 | 0 | 0 | 1.634 | 1 | 0 | 0.667 | 0 | 0.777 | Neutral |
| 1 | 514 | 45.906 | 0 | 0 | 3.318 | 1 | 0 | 0.667 | 0 | 0.775 | Neutral |
| 1 | 515 | 16.862 | 0 | 0 | 3.466 | 1 | 0 | 0.667 | 0 | 0.773 | Neutral |
| 1 | 516 | 14.948 | 0 | 0 | 2.29 | 1 | 0 | 0.667 | 0 | 0.771 | Neutral |
| 1 | 517 | 31.289 | 0 | 0 | 3.923 | 1 | 0 | 0.667 | 0 | 0.77 | Neutral |
| 1 | 518 | 0 | 0 | 1 | 0 | 0 | 0 | 1 | 0 | 1 | Invariable |
| 1 | 519 | 11.791 | 0 | 0 | 3.906 | 1 | 0 | 0.667 | 0 | 0.768 | Neutral |
| 1 | 520 | 1.793 | 0.745 | 0 | 24.884 | 1 | 0.505 | 0.428 | 0 | 1 | Neutral |
| 1 | 521 | 5.114 | 0 | 0 | 0.983 | 1 | 0 | 0.667 | 0 | 0.766 | Neutral |
| 1 | 522 | 0 | 0 | 1 | 0 | 0 | 0 | 1 | 0 | 1 | Invariable |
| 1 | 523 | 15.481 | 0 | 0 | 4.725 | 1 | 0 | 0.667 | 0 | 0.765 | Neutral |
| 1 | 524 | 5.161 | 0 | 0 | 1.008 | 1 | 0 | 0.667 | 0 | 0.763 | Neutral |
| 1 | 525 | 18.882 | 0 | 0 | 2.624 | 1 | 0 | 0.667 | 0 | 0.761 | Neutral |
| 1 | 526 | 0 | 0 | 1 | 0 | 0 | 0 | 1 | 0 | 1 | Invariable |
| 1 | 527 | 5.787 | 0 | 0 | 1.714 | 1 | 0 | 0.667 | 0 | 0.76 | Neutral |
| 1 | 528 | 2.88 | 0 | 0 | 0.678 | 1 | 0 | 0.667 | 0 | 0.758 | Neutral |
| 1 | 529 | 21.895 | 0 | 0 | 8.216 | 1 | 0 | 0.667 | 0 | 0.757 | Neutral |
| 1 | 530 | 16.817 | 0 | 0 | 3.396 | 1 | 0 | 0.667 | 0 | 0.755 | Neutral |
