# Supplementary material for: Unravelling the genomic epidemiology and antigenic evolution of Chandipura virus: A genetically constrained neurotropic threat
Source: PLoS Negl Trop Dis. 2026 Aug 3;20(8):e0014565. doi: 10.1371/journal.pntd.0014565 (PMC13489527; doi:10.1371/journal.pntd.0014565)
Supplement: Supplementary file 6 [file pntd.0014565.s006.docx]

**S6 Table. Comprehensive site-by-site MEME analysis of the Chandipura virus matrix protein gene identifying codons under episodic diversifying selection.**

| **Part.** | **Codon** | **α** | **β1** | **p1** | **β+** | **p+** | **LRT** | **p-value** | **# branches under selection** | **q** | **Class** |
| --- | --- | --- | --- | --- | --- | --- | --- | --- | --- | --- | --- |
| 1 | 1 | 0 | 0 | 1 | 0 | 0 | 0 | 1 | 0 | 1 | Invariable |
| 1 | 2 | 5.065 | 0 | 0 | 0.787 | 1 | 0 | 0.667 | 0 | 1 | Neutral |
| 1 | 3 | 7.43 | 0 | 0 | 2.626 | 1 | 0 | 0.667 | 0 | 1 | Neutral |
| 1 | 4 | 5.735 | 1.146 | 0 | 1.093 | 1 | 0 | 0.667 | 0 | 1 | Neutral |
| 1 | 5 | 0 | 0 | 1 | 0 | 0 | 0 | 1 | 0 | 1 | Invariable |
| 1 | 6 | 6.338 | 0 | 0 | 1.288 | 1 | 0 | 0.667 | 0 | 1 | Neutral |
| 1 | 7 | 5.692 | 0 | 0 | 0.733 | 1 | 0 | 0.667 | 0 | 1 | Neutral |
| 1 | 8 | 4.519 | 0 | 0 | 0.513 | 1 | 0 | 0.667 | 0 | 1 | Neutral |
| 1 | 9 | 17.473 | 0 | 0 | 2.77 | 1 | 0 | 0.667 | 0 | 1 | Neutral |
| 1 | 10 | 8.199 | 1.081 | 0 | 0.357 | 1 | 0 | 0.667 | 0 | 1 | Neutral |
| 1 | 11 | 0.001 | 0 | 0 | 0.992 | 1 | 0.572 | 0.41 | 0 | 1 | Neutral |
| 1 | 12 | 4.611 | 0 | 0 | 0.244 | 1 | 0 | 0.667 | 0 | 1 | Neutral |
| 1 | 13 | 0 | 0 | 1 | 0 | 0 | 0 | 1 | 0 | 1 | Invariable |
| 1 | 14 | 10.925 | 0 | 0 | 47.303 | 1 | 0.493 | 0.431 | 1 | 1 | Neutral |
| 1 | 15 | 0.001 | 0 | 0 | 1.068 | 1 | 0.434 | 0.448 | 0 | 1 | Neutral |
| 1 | 16 | 0 | 0 | 1 | 0 | 0 | 0 | 1 | 0 | 1 | Invariable |
| 1 | 17 | 3.296 | 0 | 0 | 0.551 | 1 | 0 | 0.667 | 0 | 1 | Neutral |
| 1 | 18 | 61.076 | 0 | 0 | 11.055 | 1 | 0 | 0.667 | 0 | 1 | Neutral |
| 1 | 19 | 0 | 0 | 1 | 0 | 0 | 0 | 1 | 0 | 1 | Invariable |
| **1** | **20** | **0** | **0** | **0** | **95.725** | **1** | **5.272** | **0.033** | **2** | **1** | **Diversifying** |
| 1 | 21 | 0 | 0 | 1 | 0 | 0 | 0 | 1 | 0 | 1 | Invariable |
| 1 | 22 | 8.831 | 0.89 | 0 | 2.137 | 1 | 0 | 0.667 | 0 | 1 | Neutral |
| 1 | 23 | 11.604 | 0.998 | 0 | 0.983 | 1 | 0 | 0.667 | 0 | 1 | Neutral |
| 1 | 24 | 3.634 | 1.075 | 0 | 0.986 | 1 | 0 | 0.667 | 0 | 1 | Neutral |
| 1 | 25 | 0 | 0 | 1 | 0 | 0 | 0 | 1 | 0 | 1 | Invariable |
| 1 | 26 | 0 | 0 | 1 | 0 | 0 | 0 | 1 | 0 | 1 | Invariable |
| 1 | 27 | 21.027 | 0 | 0 | 3.281 | 1 | 0 | 0.667 | 0 | 1 | Neutral |
| 1 | 28 | 24.155 | 0 | 0 | 2.517 | 1 | 0 | 0.667 | 0 | 1 | Neutral |
| 1 | 29 | 2.404 | 0 | 0 | 0.413 | 1 | 0 | 0.667 | 0 | 1 | Neutral |
| 1 | 30 | 0 | 0 | 1 | 0 | 0 | 0 | 1 | 0 | 1 | Invariable |
| 1 | 31 | 5.274 | 0 | 0 | 1.871 | 1 | 0 | 0.667 | 0 | 1 | Neutral |
| 1 | 32 | 0 | 0 | 1 | 0 | 0 | 0 | 1 | 0 | 1 | Invariable |
| 1 | 33 | 36.354 | 0 | 0 | 5.459 | 1 | 0 | 0.667 | 0 | 1 | Neutral |
| 1 | 34 | 20.275 | 0 | 0 | 3.214 | 1 | 0 | 0.667 | 0 | 1 | Neutral |
| 1 | 35 | 0 | 0 | 1 | 0 | 0 | 0 | 1 | 0 | 1 | Invariable |
| 1 | 36 | 8.21 | 0.975 | 0 | 0.899 | 1 | 0 | 0.667 | 0 | 1 | Neutral |
| 1 | 37 | 12.042 | 0 | 0 | 2.174 | 1 | 0 | 0.667 | 0 | 1 | Neutral |
| 1 | 38 | 3.158 | 0 | 0 | 0.531 | 1 | 0 | 0.667 | 0 | 1 | Neutral |
| 1 | 39 | 14.619 | 0 | 0 | 2.65 | 1 | 0 | 0.667 | 0 | 1 | Neutral |
| 1 | 40 | 3.296 | 0 | 0 | 0.551 | 1 | 0 | 0.667 | 0 | 1 | Neutral |
| 1 | 41 | 32.038 | 0 | 0 | 5.185 | 1 | 0 | 0.667 | 0 | 1 | Neutral |
| 1 | 42 | 13.741 | 0 | 0 | 2.55 | 1 | 0 | 0.667 | 0 | 1 | Neutral |
| 1 | 43 | 3.307 | 1.078 | 0 | 1.173 | 1 | 0 | 0.667 | 0 | 1 | Neutral |
| 1 | 44 | 19.84 | 0 | 0 | 4.284 | 1 | 0 | 0.667 | 0 | 1 | Neutral |
| 1 | 45 | 6.459 | 0 | 0 | 1.302 | 1 | 0 | 0.667 | 0 | 1 | Neutral |
| 1 | 46 | 5.774 | 0 | 0 | 1.309 | 1 | 0 | 0.667 | 0 | 1 | Neutral |
| 1 | 47 | 36.302 | 0 | 0 | 5.492 | 1 | 0 | 0.667 | 0 | 1 | Neutral |
| 1 | 48 | 9.766 | 0 | 0 | 2.125 | 1 | 0 | 0.667 | 0 | 1 | Neutral |
| 1 | 49 | 0 | 0 | 1 | 0 | 0 | 0 | 1 | 0 | 1 | Invariable |
| 1 | 50 | 0.002 | 0 | 0 | 0.945 | 1 | 0.288 | 0.495 | 1 | 1 | Neutral |
| 1 | 51 | 0 | 0 | 1 | 0 | 0 | 0 | 1 | 0 | 1 | Invariable |
| 1 | 52 | 18.693 | 0.798 | 0 | 2.673 | 1 | 0 | 0.667 | 0 | 1 | Neutral |
| 1 | 53 | 0 | 0 | 1 | 0 | 0 | 0 | 1 | 0 | 1 | Invariable |
| 1 | 54 | 0 | 0 | 1 | 0 | 0 | 0 | 1 | 0 | 1 | Invariable |
| 1 | 55 | 6.04 | 0 | 0 | 0.307 | 1 | 0 | 0.667 | 0 | 1 | Neutral |
| 1 | 56 | 0 | 0 | 0 | 1.318 | 1 | 0.62 | 0.398 | 0 | 1 | Neutral |
| 1 | 57 | 3.047 | 0 | 0 | 5.274 | 1 | 0.144 | 0.553 | 0 | 1 | Neutral |
| 1 | 58 | 9.614 | 0 | 0 | 1.929 | 1 | 0 | 0.667 | 0 | 1 | Neutral |
| 1 | 59 | 6.325 | 0 | 0 | 1.498 | 1 | 0 | 0.667 | 0 | 1 | Neutral |
| 1 | 60 | 12.287 | 0 | 0 | 2.529 | 1 | 0 | 0.667 | 0 | 1 | Neutral |
| 1 | 61 | 3.541 | 0 | 0 | 0.56 | 1 | 0 | 0.667 | 0 | 1 | Neutral |
| 1 | 62 | 6.342 | 0 | 0 | 0.319 | 1 | 0 | 0.667 | 0 | 1 | Neutral |
| 1 | 63 | 4.893 | 0 | 0 | 0.765 | 1 | 0 | 0.667 | 0 | 1 | Neutral |
| 1 | 64 | 7.875 | 0 | 0 | 1.507 | 1 | 0 | 0.667 | 0 | 1 | Neutral |
| 1 | 65 | 10.259 | 0 | 0 | 2.119 | 1 | 0 | 0.667 | 0 | 1 | Neutral |
| 1 | 66 | 8.551 | 0 | 0 | 1.347 | 1 | 0 | 0.667 | 0 | 1 | Neutral |
| 1 | 67 | 0 | 0 | 0 | 1.081 | 1 | 0.685 | 0.383 | 0 | 1 | Neutral |
| 1 | 68 | 0 | 0 | 1 | 0 | 0 | 0 | 1 | 0 | 1 | Invariable |
| 1 | 69 | 8.937 | 0 | 0 | 0.712 | 1 | 0 | 0.667 | 0 | 1 | Neutral |
| 1 | 70 | 6.04 | 0 | 0 | 0.307 | 1 | 0 | 0.667 | 0 | 1 | Neutral |
| 1 | 71 | 0 | 0 | 1 | 0 | 0 | 0 | 1 | 0 | 1 | Invariable |
| 1 | 72 | 7.993 | 0 | 0 | 1.523 | 1 | 0 | 0.667 | 0 | 1 | Neutral |
| 1 | 73 | 11.862 | 0 | 0 | 3.927 | 1 | 0 | 0.667 | 0 | 1 | Neutral |
| 1 | 74 | 16.045 | 0 | 0 | 2.388 | 1 | 0 | 0.667 | 0 | 1 | Neutral |
| 1 | 75 | 1.96 | 0 | 0 | 0.199 | 1 | 0 | 0.667 | 0 | 1 | Neutral |
| 1 | 76 | 18.971 | 0 | 0 | 3.779 | 1 | 0 | 0.667 | 0 | 1 | Neutral |
| 1 | 77 | 10.461 | 0 | 0 | 2.012 | 1 | 0 | 0.667 | 0 | 1 | Neutral |
| 1 | 78 | 8.378 | 0 | 0 | 1.574 | 1 | 0 | 0.667 | 0 | 1 | Neutral |
| 1 | 79 | 5.231 | 0 | 0 | 0.815 | 1 | 0 | 0.667 | 0 | 1 | Neutral |
| 1 | 80 | 5.838 | 0 | 0 | 1.211 | 1 | 0 | 0.667 | 0 | 1 | Neutral |
| 1 | 81 | 9.137 | 0 | 0 | 1.747 | 1 | 0 | 0.667 | 0 | 1 | Neutral |
| 1 | 82 | 12.187 | 0.633 | 0 | 3.314 | 1 | 0 | 0.667 | 0 | 1 | Neutral |
| 1 | 83 | 6.177 | 0 | 0 | 0.313 | 1 | 0 | 0.667 | 0 | 1 | Neutral |
| 1 | 84 | 5.231 | 0 | 0 | 0.815 | 1 | 0 | 0.667 | 0 | 1 | Neutral |
| 1 | 85 | 0 | 0 | 1 | 0 | 0 | 0 | 1 | 0 | 1 | Invariable |
| 1 | 86 | 0 | 0 | 1 | 0 | 0 | 0 | 1 | 0 | 1 | Invariable |
| 1 | 87 | 18.143 | 0 | 0 | 3.016 | 1 | 0 | 0.667 | 0 | 1 | Neutral |
| 1 | 88 | 2.422 | 0 | 0 | 0.397 | 1 | 0 | 0.667 | 0 | 1 | Neutral |
| 1 | 89 | 19.189 | 0 | 0 | 3.505 | 1 | 0 | 0.667 | 0 | 1 | Neutral |
| 1 | 90 | 2.155 | 0 | 0 | 0.372 | 1 | 0 | 0.667 | 0 | 1 | Neutral |
| 1 | 91 | 0 | 0 | 1 | 0 | 0 | 0 | 1 | 0 | 1 | Invariable |
| 1 | 92 | 10.041 | 0 | 0 | 1.78 | 1 | 0 | 0.667 | 0 | 1 | Neutral |
| 1 | 93 | 5.878 | 0 | 0 | 0.415 | 1 | 0 | 0.667 | 0 | 1 | Neutral |
| 1 | 94 | 0 | 0 | 1 | 0 | 0 | 0 | 1 | 0 | 1 | Invariable |
| 1 | 95 | 10.526 | 0 | 0 | 1.836 | 1 | 0 | 0.667 | 0 | 1 | Neutral |
| 1 | 96 | 13.941 | 0 | 0 | 2.976 | 1 | 0 | 0.667 | 0 | 1 | Neutral |
| **1** | **97** | **11.775** | **0** | **0** | **840.868** | **1** | **3.362** | **0.088** | **1** | **1** | **Diversifying** |
| 1 | 98 | 12.283 | 0 | 0 | 1.686 | 1 | 0 | 0.667 | 0 | 1 | Neutral |
| 1 | 99 | 0 | 0 | 0 | 1.048 | 1 | 0.701 | 0.379 | 0 | 1 | Neutral |
| 1 | 100 | 9.693 | 0 | 0 | 2.373 | 1 | 0 | 0.667 | 0 | 1 | Neutral |
| 1 | 101 | 3.844 | 0 | 0 | 0.629 | 1 | 0 | 0.667 | 0 | 1 | Neutral |
| 1 | 102 | 11.863 | 0 | 0 | 2.323 | 1 | 0 | 0.667 | 0 | 1 | Neutral |
| 1 | 103 | 14.015 | 0 | 0 | 2.202 | 1 | 0 | 0.667 | 0 | 1 | Neutral |
| 1 | 104 | 8.551 | 0 | 0 | 1.347 | 1 | 0 | 0.667 | 0 | 1 | Neutral |
| 1 | 105 | 13.741 | 0 | 0 | 2.176 | 1 | 0 | 0.667 | 0 | 1 | Neutral |
| 1 | 106 | 23.383 | 0 | 0 | 2.475 | 1 | 0 | 0.667 | 0 | 1 | Neutral |
| 1 | 107 | 6.51 | 0 | 0 | 0.437 | 1 | 0 | 0.667 | 0 | 1 | Neutral |
| 1 | 108 | 33.082 | 0 | 0 | 7.741 | 1 | 0 | 0.667 | 0 | 1 | Neutral |
| 1 | 109 | 9.403 | 0 | 0 | 1.997 | 1 | 0 | 0.667 | 0 | 1 | Neutral |
| 1 | 110 | 2.513 | 0 | 0 | 0.431 | 1 | 0 | 0.667 | 0 | 1 | Neutral |
| 1 | 111 | 17.021 | 0 | 0 | 2.472 | 1 | 0 | 0.667 | 0 | 1 | Neutral |
| 1 | 112 | 3.823 | 2.369 | 0 | 2.58 | 1 | 0 | 0.667 | 0 | 1 | Neutral |
| 1 | 113 | 10.572 | 0 | 0 | 2.162 | 1 | 0 | 0.667 | 0 | 1 | Neutral |
| 1 | 114 | 12.697 | 0 | 0 | 2.072 | 1 | 0 | 0.667 | 0 | 1 | Neutral |
| 1 | 115 | 4.652 | 0 | 0 | 0.342 | 1 | 0 | 0.667 | 0 | 1 | Neutral |
| 1 | 116 | 6.859 | 0 | 0 | 1.365 | 1 | 0 | 0.667 | 0 | 1 | Neutral |
| 1 | 117 | 9.858 | 0 | 0 | 3.441 | 1 | 0 | 0.667 | 0 | 1 | Neutral |
| 1 | 118 | 4.501 | 0 | 0 | 0.716 | 1 | 0 | 0.667 | 0 | 1 | Neutral |
| 1 | 119 | 22.13 | 0 | 0 | 3.39 | 1 | 0 | 0.667 | 0 | 1 | Neutral |
| 1 | 120 | 20.58 | 0 | 0 | 3.862 | 1 | 0 | 0.667 | 0 | 1 | Neutral |
| 1 | 121 | 2.474 | 0 | 0 | 0.424 | 1 | 0 | 0.667 | 0 | 1 | Neutral |
| 1 | 122 | 2.155 | 0 | 0 | 0.372 | 1 | 0 | 0.667 | 0 | 1 | Neutral |
| 1 | 123 | 20.945 | 0 | 0 | 2.589 | 1 | 0 | 0.667 | 0 | 1 | Neutral |
| 1 | 124 | 3.079 | 0 | 0 | 0.519 | 1 | 0 | 0.667 | 0 | 1 | Neutral |
| 1 | 125 | 6.537 | 0 | 0 | 1.53 | 1 | 0 | 0.667 | 0 | 1 | Neutral |
| 1 | 126 | 4.893 | 0 | 0 | 1.175 | 1 | 0 | 0.667 | 0 | 1 | Neutral |
| 1 | 127 | 8.864 | 0 | 0 | 1.774 | 1 | 0 | 0.667 | 0 | 1 | Neutral |
| 1 | 128 | 32.508 | 0 | 0 | 4.737 | 1 | 0 | 0.667 | 0 | 1 | Neutral |
| 1 | 129 | 4.18 | 0 | 0 | 0.815 | 1 | 0 | 0.667 | 0 | 1 | Neutral |
| 1 | 130 | 2.404 | 0 | 0 | 0.413 | 1 | 0 | 0.667 | 0 | 1 | Neutral |
| 1 | 131 | 27.888 | 0 | 0 | 3.718 | 1 | 0 | 0.667 | 0 | 1 | Neutral |
| 1 | 132 | 23.954 | 0 | 0 | 61.477 | 1 | 0.222 | 0.519 | 1 | 1 | Neutral |
| 1 | 133 | 0 | 0 | 1 | 0 | 0 | 0 | 1 | 0 | 1 | Invariable |
| 1 | 134 | 6.341 | 0 | 0 | 0.492 | 1 | 0 | 0.667 | 0 | 1 | Neutral |
| 1 | 135 | 22.311 | 0 | 0 | 2.449 | 1 | 0 | 0.667 | 0 | 1 | Neutral |
| 1 | 136 | 7.689 | 0 | 0 | 1.482 | 1 | 0 | 0.667 | 0 | 1 | Neutral |
| 1 | 137 | 5.605 | 0 | 0 | 1.174 | 1 | 0 | 0.667 | 0 | 1 | Neutral |
| 1 | 138 | 2.727 | 0 | 0 | 0.465 | 1 | 0 | 0.667 | 0 | 1 | Neutral |
| 1 | 139 | 10.526 | 0 | 0 | 1.836 | 1 | 0 | 0.667 | 0 | 1 | Neutral |
| 1 | 140 | 6.706 | 0 | 0 | 1.293 | 1 | 0 | 0.667 | 0 | 1 | Neutral |
| 1 | 141 | 5.866 | 0 | 0 | 1.47 | 1 | 0 | 0.667 | 0 | 1 | Neutral |
| 1 | 142 | 24.033 | 0 | 0 | 3.982 | 1 | 0 | 0.667 | 0 | 1 | Neutral |
| 1 | 143 | 10.382 | 0 | 0 | 2.49 | 1 | 0 | 0.667 | 0 | 1 | Neutral |
| 1 | 144 | 3.532 | 0 | 0 | 0.585 | 1 | 0 | 0.667 | 0 | 1 | Neutral |
| 1 | 145 | 18.916 | 0 | 0 | 3.089 | 1 | 0 | 0.667 | 0 | 1 | Neutral |
| 1 | 146 | 0 | 0 | 1 | 0 | 0 | 0 | 1 | 0 | 1 | Invariable |
| 1 | 147 | 9.899 | 0 | 0 | 1.763 | 1 | 0 | 0.667 | 0 | 1 | Neutral |
| 1 | 148 | 25.603 | 0 | 0 | 2.192 | 1 | 0 | 0.667 | 0 | 1 | Neutral |
| 1 | 149 | 3.296 | 0 | 0 | 0.551 | 1 | 0 | 0.667 | 0 | 1 | Neutral |
| 1 | 150 | 7.957 | 0 | 0 | 1.518 | 1 | 0 | 0.667 | 0 | 1 | Neutral |
| 1 | 151 | 8.81 | 6.124 | 0 | 7.838 | 1 | 0 | 0.667 | 0 | 1 | Neutral |
| 1 | 152 | 9.748 | 0 | 0 | 2.039 | 1 | 0 | 0.667 | 0 | 1 | Neutral |
| 1 | 153 | 13.11 | 0 | 0 | 3.368 | 1 | 0 | 0.667 | 0 | 1 | Neutral |
| 1 | 154 | 0 | 0 | 1 | 0 | 0 | 0 | 1 | 0 | 1 | Invariable |
| 1 | 155 | 0 | 0 | 1 | 0 | 0 | 0 | 1 | 0 | 1 | Invariable |
| 1 | 156 | 0 | 0 | 1 | 0 | 0 | 0 | 1 | 0 | 1 | Invariable |
| 1 | 157 | 5.628 | 0 | 0 | 1.269 | 1 | 0 | 0.667 | 0 | 1 | Neutral |
| 1 | 158 | 2.999 | 0 | 0 | 0.507 | 1 | 0 | 0.667 | 0 | 1 | Neutral |
| 1 | 159 | 12.884 | 0 | 0 | 2.091 | 1 | 0 | 0.667 | 0 | 1 | Neutral |
| 1 | 160 | 2.919 | 0 | 0 | 0.495 | 1 | 0 | 0.667 | 0 | 1 | Neutral |
| 1 | 161 | 12.697 | 0 | 0 | 2.072 | 1 | 0 | 0.667 | 0 | 1 | Neutral |
| 1 | 162 | 6.224 | 0 | 0 | 1.48 | 1 | 0 | 0.667 | 0 | 1 | Neutral |
| 1 | 163 | 10.985 | 0 | 0 | 2.441 | 1 | 0 | 0.667 | 0 | 1 | Neutral |
| 1 | 164 | 8.853 | 0 | 0 | 1.612 | 1 | 0 | 0.667 | 0 | 1 | Neutral |
| 1 | 165 | 0 | 0 | 1 | 0 | 0 | 0 | 1 | 0 | 1 | Invariable |
| 1 | 166 | 18.777 | 0 | 0 | 3.076 | 1 | 0 | 0.667 | 0 | 1 | Neutral |
| 1 | 167 | 6.847 | 0 | 0 | 1.359 | 1 | 0 | 0.667 | 0 | 1 | Neutral |
| 1 | 168 | 20.064 | 0 | 0 | 2.92 | 1 | 0 | 0.667 | 0 | 1 | Neutral |
| 1 | 169 | 9.66 | 0 | 0 | 2.027 | 1 | 0 | 0.667 | 0 | 1 | Neutral |
| 1 | 170 | 30.65 | 0 | 0 | 4.008 | 1 | 0 | 0.667 | 0 | 1 | Neutral |
| 1 | 171 | 4.138 | 0 | 0 | 0.668 | 1 | 0 | 0.667 | 0 | 1 | Neutral |
| 1 | 172 | 0 | 0 | 1 | 0 | 0 | 0 | 1 | 0 | 1 | Invariable |
| 1 | 173 | 0 | 0 | 1 | 0 | 0 | 0 | 1 | 0 | 1 | Invariable |
| 1 | 174 | 16.823 | 0 | 0 | 2.702 | 1 | 0 | 0.667 | 0 | 1 | Neutral |
| 1 | 175 | 0 | 0 | 1 | 0 | 0 | 0 | 1 | 0 | 1 | Invariable |
| 1 | 176 | 8.726 | 0 | 0 | 3.153 | 1 | 0 | 0.667 | 0 | 1 | Neutral |
| 1 | 177 | 4.853 | 0 | 0 | 0.374 | 1 | 0 | 0.667 | 0 | 1 | Neutral |
| 1 | 178 | 0 | 0 | 1 | 0 | 0 | 0 | 1 | 0 | 1 | Invariable |
| 1 | 179 | 0 | 0 | 1 | 0 | 0 | 0 | 1 | 0 | 1 | Invariable |
| 1 | 180 | 6.722 | 0 | 0 | 1.456 | 1 | 0 | 0.667 | 0 | 1 | Neutral |
| 1 | 181 | 30.377 | 0 | 0 | 3.99 | 1 | 0 | 0.667 | 0 | 1 | Neutral |
| 1 | 182 | 6.338 | 0 | 0 | 1.288 | 1 | 0 | 0.667 | 0 | 1 | Neutral |
| 1 | 183 | 6.47 | 0 | 0 | 1.308 | 1 | 0 | 0.667 | 0 | 1 | Neutral |
| 1 | 184 | 47.419 | 0 | 0 | 11.2 | 1 | 0 | 0.667 | 0 | 0.998 | Neutral |
| 1 | 185 | 4.753 | 0 | 0 | 1.132 | 1 | 0 | 0.667 | 0 | 0.991 | Neutral |
| 1 | 186 | 15.182 | 0 | 0 | 2.822 | 1 | 0 | 0.667 | 0 | 0.985 | Neutral |
| 1 | 187 | 14.626 | 0 | 0 | 4.55 | 1 | 0 | 0.667 | 0 | 0.979 | Neutral |
| 1 | 188 | 43.199 | 0 | 0 | 4.911 | 1 | 0 | 0.667 | 0 | 0.972 | Neutral |
| 1 | 189 | 13.683 | 0 | 0 | 2.654 | 1 | 0 | 0.667 | 0 | 0.966 | Neutral |
| 1 | 190 | 32.229 | 0 | 0 | 3.598 | 1 | 0 | 0.667 | 0 | 0.96 | Neutral |
| 1 | 191 | 18.464 | 0 | 0 | 3.589 | 1 | 0 | 0.667 | 0 | 0.954 | Neutral |
| 1 | 192 | 0 | 0 | 1 | 0 | 0 | 0 | 1 | 0 | 1 | Invariable |
| 1 | 193 | 26.755 | 0 | 0 | 2.518 | 1 | 0 | 0.667 | 0 | 0.948 | Neutral |
| 1 | 194 | 3.108 | 0 | 0 | 0.523 | 1 | 0 | 0.667 | 0 | 0.942 | Neutral |
| 1 | 195 | 10.839 | 0 | 0 | 3.686 | 1 | 0 | 0.667 | 0 | 0.937 | Neutral |
| 1 | 196 | 5.527 | 2.322 | 0 | 2.025 | 1 | 0 | 0.667 | 0 | 0.931 | Neutral |
| 1 | 197 | 0 | 0 | 0 | 2.23 | 1 | 1.034 | 0.311 | 0 | 1 | Neutral |
| 1 | 198 | 3.487 | 1.317 | 0 | 1.281 | 1 | 0 | 0.667 | 0 | 0.925 | Neutral |
| 1 | 199 | 11.844 | 0 | 0 | 2.47 | 1 | 0 | 0.667 | 0 | 0.92 | Neutral |
| 1 | 200 | 12.291 | 0 | 0 | 2.03 | 1 | 0 | 0.667 | 0 | 0.914 | Neutral |
| 1 | 201 | 3.865 | 0 | 0 | 0.631 | 1 | 0 | 0.667 | 0 | 0.909 | Neutral |
| 1 | 202 | 6.458 | 0 | 0 | 1.58 | 1 | 0 | 0.667 | 0 | 0.903 | Neutral |
| 1 | 203 | 10.526 | 0 | 0 | 1.836 | 1 | 0 | 0.667 | 0 | 0.898 | Neutral |
| 1 | 204 | 0 | 0 | 1 | 0 | 0 | 0 | 1 | 0 | 1 | Invariable |
| 1 | 205 | 11.947 | 0 | 0 | 2.333 | 1 | 0 | 0.667 | 0 | 0.893 | Neutral |
| 1 | 206 | 0 | 0 | 1 | 0 | 0 | 0 | 1 | 0 | 1 | Invariable |
| 1 | 207 | 9.216 | 0 | 0 | 1.68 | 1 | 0 | 0.667 | 0 | 0.888 | Neutral |
| 1 | 208 | 2.271 | 0 | 0 | 0.391 | 1 | 0 | 0.667 | 0 | 0.882 | Neutral |
| 1 | 209 | 8.192 | 0 | 0 | 1.549 | 1 | 0 | 0.667 | 0 | 0.877 | Neutral |
| 1 | 210 | 18.233 | 0 | 0 | 2.572 | 1 | 0 | 0.667 | 0 | 0.872 | Neutral |
| 1 | 211 | 0 | 0 | 1 | 0 | 0 | 0 | 1 | 0 | 1 | Invariable |
| 1 | 212 | 8.156 | 0 | 0 | 1.802 | 1 | 0 | 0.667 | 0 | 0.867 | Neutral |
| 1 | 213 | 19.387 | 0 | 0 | 4.425 | 1 | 0 | 0.667 | 0 | 0.863 | Neutral |
| 1 | 214 | 0 | 0 | 1 | 0 | 0 | 0 | 1 | 0 | 1 | Invariable |
| 1 | 215 | 59.77 | 0 | 0 | 8.475 | 1 | 0 | 0.667 | 0 | 0.858 | Neutral |
| 1 | 216 | 6.338 | 0 | 0 | 1.288 | 1 | 0 | 0.667 | 0 | 0.853 | Neutral |
| 1 | 217 | 10.793 | 0 | 0 | 1.867 | 1 | 0 | 0.667 | 0 | 0.848 | Neutral |
| 1 | 218 | 69.2 | 0 | 0 | 6.324 | 1 | 0 | 0.667 | 0 | 0.843 | Neutral |
| 1 | 219 | 30 | 0 | 0 | 1.898 | 1 | 0 | 0.667 | 0 | 0.839 | Neutral |
| 1 | 220 | 22.412 | 0 | 0 | 2.457 | 1 | 0 | 0.667 | 0 | 0.834 | Neutral |
| 1 | 221 | 0 | 0 | 1 | 0 | 0 | 0 | 1 | 0 | 1 | Invariable |
| 1 | 222 | 7.478 | 0 | 0 | 1.453 | 1 | 0 | 0.667 | 0 | 0.83 | Neutral |
| 1 | 223 | 0 | 0 | 1 | 0 | 0 | 0 | 1 | 0 | 1 | Invariable |
| 1 | 224 | 7.5 | 0 | 0 | 0.771 | 1 | 0 | 0.667 | 0 | 0.825 | Neutral |
| 1 | 225 | 6.784 | 0 | 0 | 1.354 | 1 | 0 | 0.667 | 0 | 0.821 | Neutral |
| 1 | 226 | 9.014 | 0 | 0 | 1.794 | 1 | 0 | 0.667 | 0 | 0.816 | Neutral |
| 1 | 227 | 6.338 | 0 | 0 | 1.288 | 1 | 0 | 0.667 | 0 | 0.812 | Neutral |
| 1 | 228 | 5.592 | 0 | 0 | 1.172 | 1 | 0 | 0.667 | 0 | 0.808 | Neutral |
| 1 | 229 | 35.183 | 0 | 0 | 17.387 | 1 | 0 | 0.667 | 0 | 0.804 | Neutral |
