# Supplementary material for: Unravelling the genomic epidemiology and antigenic evolution of Chandipura virus: A genetically constrained neurotropic threat
Source: PLoS Negl Trop Dis. 2026 Aug 3;20(8):e0014565. doi: 10.1371/journal.pntd.0014565 (PMC13489527; doi:10.1371/journal.pntd.0014565)
Supplement: Supplementary file 7 [file pntd.0014565.s007.docx]

**S7 Table. Comprehensive site-by-site MEME analysis of the Chandipura virus nucleoprotein gene identifying codons under episodic diversifying selection.**

| **Part.** | **Codon** | **α** | **β1** | **p1** | **β+** | **p+** | **LRT** | **p-value** | **# branches under selection** | **q** | **Class** |
| --- | --- | --- | --- | --- | --- | --- | --- | --- | --- | --- | --- |
| 1 | 1 | 0 | 0 | 1 | 0 | 0 | 0 | 1 | 0 | 1 | Invariable |
| 1 | 2 | 7.657 | 0 | 0 | 15.345 | 1 | 0.125 | 0.561 | 0 | 1 | Neutral |
| 1 | 3 | 0 | 0 | 1 | 0 | 0 | 0 | 1 | 0 | 1 | Invariable |
| 1 | 4 | 19.491 | 1.595 | 0 | 1.651 | 1 | 0 | 0.667 | 0 | 1 | Neutral |
| 1 | 5 | 9.165 | 0 | 0 | 1.979 | 1 | 0 | 0.667 | 0 | 1 | Neutral |
| 1 | 6 | 6.806 | 0 | 0 | 16.87 | 1 | 0.24 | 0.512 | 1 | 1 | Neutral |
| 1 | 7 | 0 | 0 | 1 | 0 | 0 | 0 | 1 | 0 | 1 | Invariable |
| 1 | 8 | 0 | 0 | 1 | 0 | 0 | 0 | 1 | 0 | 1 | Invariable |
| 1 | 9 | 0 | 0 | 0 | 1.446 | 1 | 0.739 | 0.37 | 1 | 1 | Neutral |
| 1 | 10 | 0 | 0 | 1 | 0 | 0 | 0 | 1 | 0 | 1 | Invariable |
| 1 | 11 | 21.931 | 0 | 0 | 2.599 | 1 | 0 | 0.667 | 0 | 1 | Neutral |
| 1 | 12 | 4.595 | 0 | 0 | 0.353 | 1 | 0 | 0.667 | 0 | 1 | Neutral |
| 1 | 13 | 0 | 0 | 1 | 0 | 0 | 0 | 1 | 0 | 1 | Invariable |
| 1 | 14 | 4.147 | 0 | 0 | 0.425 | 1 | 0 | 0.667 | 0 | 1 | Neutral |
| 1 | 15 | 4.491 | 0 | 0 | 0.715 | 1 | 0 | 0.667 | 0 | 1 | Neutral |
| 1 | 16 | 6.882 | 0 | 0 | 0.612 | 1 | 0 | 0.667 | 0 | 1 | Neutral |
| 1 | 17 | 7.932 | 0 | 0 | 0.787 | 1 | 0 | 0.667 | 0 | 1 | Neutral |
| 1 | 18 | 4.462 | 0 | 0 | 0.336 | 1 | 0 | 0.667 | 0 | 1 | Neutral |
| 1 | 19 | 7.963 | 0 | 0 | 2.931 | 1 | 0 | 0.667 | 0 | 1 | Neutral |
| 1 | 20 | 14.963 | 0 | 0 | 2.585 | 1 | 0 | 0.667 | 0 | 1 | Neutral |
| 1 | 21 | 28.822 | 0 | 0 | 3.467 | 1 | 0 | 0.667 | 0 | 1 | Neutral |
| 1 | 22 | 0 | 0 | 1 | 0 | 0 | 0 | 1 | 0 | 1 | Invariable |
| 1 | 23 | 0 | 0 | 1 | 0 | 0 | 0 | 1 | 0 | 1 | Invariable |
| 1 | 24 | 3.046 | 0 | 0 | 0.356 | 1 | 0 | 0.667 | 0 | 1 | Neutral |
| 1 | 25 | 2.81 | 0 | 0 | 0.288 | 1 | 0 | 0.667 | 0 | 1 | Neutral |
| 1 | 26 | 4.138 | 0 | 0 | 0.668 | 1 | 0 | 0.667 | 0 | 1 | Neutral |
| 1 | 27 | 0 | 0 | 1 | 0 | 0 | 0 | 1 | 0 | 1 | Invariable |
| 1 | 28 | 0 | 0 | 1 | 0 | 0 | 0 | 1 | 0 | 1 | Invariable |
| 1 | 29 | 46.58 | 1.102 | 0 | 1.44 | 1 | 0 | 0.667 | 0 | 1 | Neutral |
| 1 | 30 | 7.281 | 0 | 0 | 106.658 | 1 | 1.169 | 0.288 | 1 | 1 | Neutral |
| 1 | 31 | 0 | 0 | 1 | 0 | 0 | 0 | 1 | 0 | 1 | Invariable |
| 1 | 32 | 0 | 0 | 1 | 0 | 0 | 0 | 1 | 0 | 1 | Invariable |
| 1 | 33 | 0 | 0 | 1 | 0 | 0 | 0 | 1 | 0 | 1 | Invariable |
| 1 | 34 | 26.06 | 0 | 0 | 4.626 | 1 | 0 | 0.667 | 0 | 1 | Neutral |
| **1** | **35** | **0** | **0** | **0** | **27.13** | **1** | **3.904** | **0.067** | **1** | **1** | **Diversifying** |
| 1 | 36 | 26.343 | 0 | 0 | 5.069 | 1 | 0 | 0.667 | 0 | 1 | Neutral |
| 1 | 37 | 27.904 | 1.349 | 0 | 1.219 | 1 | 0 | 0.667 | 0 | 1 | Neutral |
| 1 | 38 | 0 | 0 | 1 | 0 | 0 | 0 | 1 | 0 | 1 | Invariable |
| 1 | 39 | 3.943 | 0 | 0 | 0.613 | 1 | 0 | 0.667 | 0 | 1 | Neutral |
| 1 | 40 | 26.017 | 0 | 0 | 1.974 | 1 | 0 | 0.667 | 0 | 1 | Neutral |
| 1 | 41 | 17.809 | 0 | 0 | 4.013 | 1 | 0 | 0.667 | 0 | 1 | Neutral |
| 1 | 42 | 5.141 | 0 | 0 | 0.796 | 1 | 0 | 0.667 | 0 | 1 | Neutral |
| 1 | 43 | 4.045 | 0 | 0 | 0.627 | 1 | 0 | 0.667 | 0 | 1 | Neutral |
| 1 | 44 | 48.831 | 0 | 0 | 4.191 | 1 | 0 | 0.667 | 0 | 1 | Neutral |
| 1 | 45 | 6.057 | 0 | 0 | 0.362 | 1 | 0 | 0.667 | 0 | 1 | Neutral |
| 1 | 46 | 0 | 0 | 1 | 0 | 0 | 0 | 1 | 0 | 1 | Invariable |
| 1 | 47 | 14.194 | 1.356 | 0 | 1.305 | 1 | 0 | 0.667 | 0 | 1 | Neutral |
| 1 | 48 | 0.015 | 0.006 | 0 | 50.973 | 1 | 1.651 | 0.22 | 1 | 1 | Neutral |
| 1 | 49 | 2.315 | 0 | 0 | 0.399 | 1 | 0 | 0.667 | 0 | 1 | Neutral |
| 1 | 50 | 4.923 | 0 | 0 | 1.109 | 1 | 0 | 0.667 | 0 | 1 | Neutral |
| 1 | 51 | 20.111 | 3.08 | 0 | 2.102 | 1 | 0 | 0.667 | 0 | 1 | Neutral |
| 1 | 52 | 11.158 | 0 | 0 | 1.027 | 1 | 0 | 0.667 | 0 | 1 | Neutral |
| 1 | 53 | 5.242 | 0 | 0 | 0.808 | 1 | 0 | 0.667 | 0 | 1 | Neutral |
| 1 | 54 | 0 | 0 | 1 | 0 | 0 | 0 | 1 | 0 | 1 | Invariable |
| 1 | 55 | 7.647 | 0 | 0 | 1.08 | 1 | 0 | 0.667 | 0 | 1 | Neutral |
| 1 | 56 | 27.621 | 0 | 0 | 3.976 | 1 | 0 | 0.667 | 0 | 1 | Neutral |
| 1 | 57 | 8.322 | 0 | 0 | 1.083 | 1 | 0 | 0.667 | 0 | 1 | Neutral |
| 1 | 58 | 40.751 | 1.325 | 0 | 5.419 | 1 | 0 | 0.667 | 0 | 1 | Neutral |
| 1 | 59 | 3.846 | 0 | 0 | 0.629 | 1 | 0 | 0.667 | 0 | 1 | Neutral |
| 1 | 60 | 3.129 | 1.591 | 0 | 1.608 | 1 | 0 | 0.667 | 0 | 1 | Neutral |
| 1 | 61 | 50.59 | 0 | 0 | 8.962 | 1 | 0 | 0.667 | 0 | 1 | Neutral |
| 1 | 62 | 12.067 | 0 | 0 | 2.378 | 1 | 0 | 0.667 | 0 | 1 | Neutral |
| 1 | 63 | 3.846 | 0 | 0 | 0.629 | 1 | 0 | 0.667 | 0 | 1 | Neutral |
| 1 | 64 | 18.025 | 0 | 0 | 9.949 | 1 | 0 | 0.667 | 0 | 1 | Neutral |
| 1 | 65 | 37.882 | 0 | 0 | 4.325 | 1 | 0 | 0.667 | 0 | 1 | Neutral |
| 1 | 66 | 2.188 | 0 | 0 | 6.034 | 1 | 0.327 | 0.482 | 2 | 1 | Neutral |
| 1 | 67 | 3.806 | 1.821 | 0 | 1.777 | 1 | 0 | 0.667 | 0 | 1 | Neutral |
| 1 | 68 | 8.839 | 0 | 0 | 1.762 | 1 | 0 | 0.667 | 0 | 1 | Neutral |
| 1 | 69 | 1.713 | 0.129 | 0 | 1.54 | 1 | 0 | 0.667 | 0 | 1 | Neutral |
| 1 | 70 | 6.738 | 0 | 0 | 0.687 | 1 | 0 | 0.667 | 0 | 1 | Neutral |
| 1 | 71 | 28.827 | 0 | 0 | 42.225 | 1 | 0.043 | 0.61 | 1 | 1 | Neutral |
| 1 | 72 | 12.791 | 0 | 0 | 2.595 | 1 | 0 | 0.667 | 0 | 1 | Neutral |
| 1 | 73 | 19.449 | 0 | 0 | 1.219 | 1 | 0 | 0.667 | 0 | 1 | Neutral |
| 1 | 74 | 6.319 | 0 | 0 | 0.457 | 1 | 0 | 0.667 | 0 | 1 | Neutral |
| 1 | 75 | 7.808 | 0 | 0 | 1.084 | 1 | 0 | 0.667 | 0 | 1 | Neutral |
| 1 | 76 | 3.128 | 1.835 | 0 | 1.86 | 1 | 0 | 0.667 | 0 | 1 | Neutral |
| 1 | 77 | 45.301 | 0 | 0 | 81.258 | 1 | 0.084 | 0.583 | 1 | 1 | Neutral |
| 1 | 78 | 27.7 | 0 | 0 | 2.264 | 1 | 0 | 0.667 | 0 | 1 | Neutral |
| 1 | 79 | 32.58 | 0 | 0 | 207.917 | 1 | 1.876 | 0.195 | 1 | 1 | Neutral |
| 1 | 80 | 6.66 | 1.398 | 0 | 2.002 | 1 | 0 | 0.667 | 0 | 1 | Neutral |
| 1 | 81 | 5.234 | 0 | 0 | 0.807 | 1 | 0 | 0.667 | 0 | 1 | Neutral |
| 1 | 82 | 4.65 | 3.48 | 0 | 3.903 | 1 | 0 | 0.667 | 0 | 1 | Neutral |
| 1 | 83 | 34.081 | 0 | 0 | 3.875 | 1 | 0 | 0.667 | 0 | 1 | Neutral |
| 1 | 84 | 0 | 0 | 1 | 0 | 0 | 0 | 1 | 0 | 1 | Invariable |
| 1 | 85 | 3.11 | 0 | 0 | 0.523 | 1 | 0 | 0.667 | 0 | 1 | Neutral |
| 1 | 86 | 13.619 | 0 | 0 | 2.164 | 1 | 0 | 0.667 | 0 | 1 | Neutral |
| 1 | 87 | 4.675 | 0 | 0 | 0.738 | 1 | 0 | 0.667 | 0 | 1 | Neutral |
| 1 | 88 | 7.551 | 0 | 0 | 0.589 | 1 | 0 | 0.667 | 0 | 1 | Neutral |
| 1 | 89 | 0 | 0 | 1 | 0 | 0 | 0 | 1 | 0 | 1 | Invariable |
| 1 | 90 | 58.31 | 0 | 0 | 11.494 | 1 | 0 | 0.667 | 0 | 1 | Neutral |
| 1 | 91 | 22.809 | 0 | 0 | 5.595 | 1 | 0 | 0.667 | 0 | 1 | Neutral |
| 1 | 92 | 78.845 | 0 | 0 | 13.456 | 1 | 0 | 0.667 | 0 | 1 | Neutral |
| 1 | 93 | 5.481 | 0 | 0 | 0.836 | 1 | 0 | 0.667 | 0 | 1 | Neutral |
| 1 | 94 | 25.715 | 0 | 0 | 81.61 | 1 | 0.307 | 0.488 | 1 | 1 | Neutral |
| 1 | 95 | 0.001 | 0 | 0 | 4.256 | 1 | 0.677 | 0.385 | 1 | 1 | Neutral |
| 1 | 96 | 7.819 | 0 | 0 | 0.535 | 1 | 0 | 0.667 | 0 | 1 | Neutral |
| 1 | 97 | 9.891 | 0 | 0 | 2.018 | 1 | 0 | 0.667 | 0 | 1 | Neutral |
| 1 | 98 | 12.32 | 0 | 0 | 1.262 | 1 | 0 | 0.667 | 0 | 1 | Neutral |
| 1 | 99 | 6.185 | 0 | 0 | 0.313 | 1 | 0 | 0.667 | 0 | 1 | Neutral |
| 1 | 100 | 0 | 0 | 1 | 0 | 0 | 0 | 1 | 0 | 1 | Invariable |
| 1 | 101 | 0 | 0 | 0 | 59.161 | 1 | 1.88 | 0.194 | 1 | 1 | Neutral |
| 1 | 102 | 3.11 | 0 | 0 | 0.523 | 1 | 0 | 0.667 | 0 | 1 | Neutral |
| 1 | 103 | 0 | 0 | 1 | 0 | 0 | 0 | 1 | 0 | 1 | Invariable |
| 1 | 104 | 11.451 | 0 | 0 | 2.222 | 1 | 0 | 0.667 | 0 | 1 | Neutral |
| 1 | 105 | 20.36 | 0 | 0 | 3.744 | 1 | 0 | 0.667 | 0 | 1 | Neutral |
| 1 | 106 | 5.659 | 0 | 0 | 0.857 | 1 | 0 | 0.667 | 0 | 1 | Neutral |
| 1 | 107 | 0 | 0 | 1 | 0 | 0 | 0 | 1 | 0 | 1 | Invariable |
| 1 | 108 | 9.555 | 0 | 0 | 0.815 | 1 | 0 | 0.667 | 0 | 1 | Neutral |
| 1 | 109 | 12.984 | 0 | 0 | 3.91 | 1 | 0 | 0.667 | 0 | 1 | Neutral |
| 1 | 110 | 27.824 | 0 | 0 | 7.222 | 1 | 0 | 0.667 | 0 | 1 | Neutral |
| 1 | 111 | 10.282 | 0 | 0 | 2.197 | 1 | 0 | 0.667 | 0 | 1 | Neutral |
| 1 | 112 | 41.885 | 0 | 0 | 6.942 | 1 | 0 | 0.667 | 0 | 1 | Neutral |
| 1 | 113 | 49.958 | 0 | 0 | 7.03 | 1 | 0 | 0.667 | 0 | 1 | Neutral |
| 1 | 114 | 7.469 | 3.172 | 0 | 3.097 | 1 | 0 | 0.667 | 0 | 1 | Neutral |
| 1 | 115 | 0 | 0 | 0 | 1.404 | 1 | 0.31 | 0.487 | 0 | 1 | Neutral |
| 1 | 116 | 14.421 | 0 | 0 | 2.241 | 1 | 0 | 0.667 | 0 | 1 | Neutral |
| 1 | 117 | 20.693 | 1.966 | 0 | 1.723 | 1 | 0 | 0.667 | 0 | 1 | Neutral |
| 1 | 118 | 7.623 | 3.98 | 0 | 4.117 | 1 | 0 | 0.667 | 0 | 1 | Neutral |
| 1 | 119 | 9.605 | 0 | 0 | 1.382 | 1 | 0 | 0.667 | 0 | 1 | Neutral |
| 1 | 120 | 19.543 | 0 | 0 | 2.249 | 1 | 0 | 0.667 | 0 | 1 | Neutral |
| 1 | 121 | 52.753 | 0 | 0 | 4.581 | 1 | 0 | 0.667 | 0 | 1 | Neutral |
| 1 | 122 | 4.675 | 0 | 0 | 0.738 | 1 | 0 | 0.667 | 0 | 1 | Neutral |
| 1 | 123 | 9.015 | 0 | 0 | 2.009 | 1 | 0 | 0.667 | 0 | 1 | Neutral |
| 1 | 124 | 26.6 | 0 | 0 | 4.221 | 1 | 0 | 0.667 | 0 | 1 | Neutral |
| 1 | 125 | 5.412 | 0 | 0 | 0.828 | 1 | 0 | 0.667 | 0 | 1 | Neutral |
| 1 | 126 | 16.24 | 0 | 0 | 4.851 | 1 | 0 | 0.667 | 0 | 1 | Neutral |
| 1 | 127 | 8.508 | 0 | 0 | 1.263 | 1 | 0 | 0.667 | 0 | 1 | Neutral |
| 1 | 128 | 19.111 | 0 | 0 | 1.916 | 1 | 0 | 0.667 | 0 | 1 | Neutral |
| 1 | 129 | 24.019 | 0 | 0 | 3.545 | 1 | 0 | 0.667 | 0 | 1 | Neutral |
| 1 | 130 | 2.697 | 0 | 0 | 0.214 | 1 | 0 | 0.667 | 0 | 1 | Neutral |
| 1 | 131 | 8.869 | 0 | 0 | 0.411 | 1 | 0 | 0.667 | 0 | 1 | Neutral |
| 1 | 132 | 13.619 | 0 | 0 | 2.164 | 1 | 0 | 0.667 | 0 | 1 | Neutral |
| 1 | 133 | 4.832 | 0 | 0 | 15.143 | 1 | 0.467 | 0.439 | 1 | 1 | Neutral |
| 1 | 134 | 0 | 0 | 1 | 0 | 0 | 0 | 1 | 0 | 1 | Invariable |
| 1 | 135 | 4.462 | 0 | 0 | 0.336 | 1 | 0 | 0.667 | 0 | 1 | Neutral |
| 1 | 136 | 5.544 | 0 | 0 | 0.844 | 1 | 0 | 0.667 | 0 | 1 | Neutral |
| 1 | 137 | 7.331 | 0 | 0 | 0.644 | 1 | 0 | 0.667 | 0 | 1 | Neutral |
| 1 | 138 | 2.208 | 0 | 0 | 0.303 | 1 | 0 | 0.667 | 0 | 1 | Neutral |
| 1 | 139 | 10.497 | 0 | 0 | 2.185 | 1 | 0 | 0.667 | 0 | 1 | Neutral |
| 1 | 140 | 19.211 | 0 | 0 | 122.552 | 1 | 0.7 | 0.379 | 1 | 1 | Neutral |
| 1 | 141 | 11.734 | 0 | 0 | 1.361 | 1 | 0 | 0.667 | 0 | 1 | Neutral |
| 1 | 142 | 13.973 | 0 | 0 | 4.107 | 1 | 0 | 0.667 | 0 | 1 | Neutral |
| 1 | 143 | 27.06 | 0 | 0 | 3.659 | 1 | 0 | 0.667 | 0 | 1 | Neutral |
| 1 | 144 | 12.834 | 0 | 0 | 2.934 | 1 | 0 | 0.667 | 0 | 1 | Neutral |
| 1 | 145 | 17.315 | 0 | 0 | 2.82 | 1 | 0 | 0.667 | 0 | 1 | Neutral |
| 1 | 146 | 3.087 | 0 | 0 | 0.311 | 1 | 0 | 0.667 | 0 | 1 | Neutral |
| 1 | 147 | 8.156 | 0 | 0 | 0.717 | 1 | 0 | 0.667 | 0 | 1 | Neutral |
| 1 | 148 | 25.183 | 0 | 0 | 3.636 | 1 | 0 | 0.667 | 0 | 1 | Neutral |
| 1 | 149 | 5.354 | 0 | 0 | 0.821 | 1 | 0 | 0.667 | 0 | 1 | Neutral |
| 1 | 150 | 6.011 | 0 | 0 | 0.897 | 1 | 0 | 0.667 | 0 | 1 | Neutral |
| 1 | 151 | 22.246 | 0 | 0 | 3.399 | 1 | 0 | 0.667 | 0 | 1 | Neutral |
| 1 | 152 | 5.972 | 0 | 0 | 0.553 | 1 | 0 | 0.667 | 0 | 1 | Neutral |
| 1 | 153 | 0 | 0 | 1 | 0 | 0 | 0 | 1 | 0 | 1 | Invariable |
| 1 | 154 | 7.573 | 0 | 0 | 3.456 | 1 | 0 | 0.667 | 0 | 1 | Neutral |
| 1 | 155 | 4.138 | 0 | 0 | 0.668 | 1 | 0 | 0.667 | 0 | 1 | Neutral |
| 1 | 156 | 4.454 | 0 | 0 | 0.71 | 1 | 0 | 0.667 | 0 | 1 | Neutral |
| 1 | 157 | 3.808 | 0 | 0 | 0.68 | 1 | 0 | 0.667 | 0 | 1 | Neutral |
| 1 | 158 | 0 | 0 | 1 | 0 | 0 | 0 | 1 | 0 | 1 | Invariable |
| 1 | 159 | 3.317 | 0 | 0 | 0.554 | 1 | 0 | 0.667 | 0 | 1 | Neutral |
| 1 | 160 | 30.479 | 0 | 0 | 3.98 | 1 | 0 | 0.667 | 0 | 1 | Neutral |
| 1 | 161 | 14.642 | 0 | 0 | 2.663 | 1 | 0 | 0.667 | 0 | 1 | Neutral |
| 1 | 162 | 7.725 | 0 | 0 | 1.076 | 1 | 0 | 0.667 | 0 | 1 | Neutral |
| 1 | 163 | 26.646 | 0 | 0 | 59.152 | 1 | 0.136 | 0.556 | 1 | 1 | Neutral |
| 1 | 164 | 4.113 | 0 | 0 | 0.61 | 1 | 0 | 0.667 | 0 | 1 | Neutral |
| 1 | 165 | 13.379 | 0 | 0 | 2.14 | 1 | 0 | 0.667 | 0 | 1 | Neutral |
| 1 | 166 | 24.149 | 0 | 0 | 2.27 | 1 | 0 | 0.667 | 0 | 1 | Neutral |
| 1 | 167 | 0.004 | 0 | 0 | 1.27 | 1 | 0.067 | 0.593 | 1 | 1 | Neutral |
| 1 | 168 | 23.091 | 0 | 0 | 3.47 | 1 | 0 | 0.667 | 0 | 1 | Neutral |
| 1 | 169 | 18.347 | 0 | 0 | 45.179 | 1 | 0.195 | 0.53 | 1 | 1 | Neutral |
| 1 | 170 | 56.25 | 0 | 0 | 9.329 | 1 | 0 | 0.667 | 0 | 1 | Neutral |
| 1 | 171 | 0 | 0 | 1 | 0 | 0 | 0 | 1 | 0 | 1 | Invariable |
| 1 | 172 | 2.739 | 0 | 0 | 0.599 | 1 | 0 | 0.667 | 0 | 1 | Neutral |
| 1 | 173 | 36.055 | 0 | 0 | 7.547 | 1 | 0 | 0.667 | 0 | 1 | Neutral |
| 1 | 174 | 40.517 | 0 | 0 | 6.594 | 1 | 0 | 0.667 | 0 | 1 | Neutral |
| 1 | 175 | 5.918 | 0 | 0 | 0.887 | 1 | 0 | 0.667 | 0 | 1 | Neutral |
| 1 | 176 | 10.78 | 0 | 0 | 0.864 | 1 | 0 | 0.667 | 0 | 1 | Neutral |
| 1 | 177 | 7.9 | 0 | 0 | 0.378 | 1 | 0 | 0.667 | 0 | 1 | Neutral |
| 1 | 178 | 11.646 | 0 | 0 | 3.856 | 1 | 0 | 0.667 | 0 | 1 | Neutral |
| 1 | 179 | 11.646 | 0 | 0 | 4.046 | 1 | 0 | 0.667 | 0 | 1 | Neutral |
| 1 | 180 | 11.855 | 0 | 0 | 1.802 | 1 | 0 | 0.667 | 0 | 1 | Neutral |
| 1 | 181 | 6.718 | 0 | 0 | 0.974 | 1 | 0 | 0.667 | 0 | 1 | Neutral |
| 1 | 182 | 26.456 | 0 | 0 | 4.728 | 1 | 0 | 0.667 | 0 | 1 | Neutral |
| 1 | 183 | 7.877 | 0 | 0 | 1.091 | 1 | 0 | 0.667 | 0 | 1 | Neutral |
| 1 | 184 | 7.802 | 0 | 0 | 0.603 | 1 | 0 | 0.667 | 0 | 1 | Neutral |
| 1 | 185 | 4.045 | 0 | 0 | 0.627 | 1 | 0 | 0.667 | 0 | 1 | Neutral |
| 1 | 186 | 0 | 0 | 1 | 0 | 0 | 0 | 1 | 0 | 1 | Invariable |
| **1** | **187** | **0** | **0** | **0** | **2,476.83** | **1** | **3.651** | **0.076** | **1** | **1** | **Diversifying** |
| 1 | 188 | 7.553 | 0 | 0 | 1.071 | 1 | 0 | 0.667 | 0 | 1 | Neutral |
| 1 | 189 | 0 | 0 | 1 | 0 | 0 | 0 | 1 | 0 | 1 | Invariable |
| 1 | 190 | 12.239 | 0 | 0 | 3.992 | 1 | 0 | 0.667 | 0 | 1 | Neutral |
| 1 | 191 | 64.224 | 0 | 0 | 10.593 | 1 | 0 | 0.667 | 0 | 1 | Neutral |
| 1 | 192 | 5.412 | 0 | 0 | 0.828 | 1 | 0 | 0.667 | 0 | 1 | Neutral |
| 1 | 193 | 21.413 | 0 | 0 | 3.328 | 1 | 0 | 0.667 | 0 | 1 | Neutral |
| 1 | 194 | 4.675 | 0 | 0 | 0.738 | 1 | 0 | 0.667 | 0 | 1 | Neutral |
| 1 | 195 | 4.402 | 0 | 0 | 0.672 | 1 | 0 | 0.667 | 0 | 1 | Neutral |
| 1 | 196 | 8.089 | 0 | 0 | 1.02 | 1 | 0 | 0.667 | 0 | 1 | Neutral |
| 1 | 197 | 3.705 | 0 | 0 | 0.609 | 1 | 0 | 0.667 | 0 | 1 | Neutral |
| 1 | 198 | 0 | 0 | 1 | 0 | 0 | 0 | 1 | 0 | 1 | Invariable |
| 1 | 199 | 0 | 0 | 1 | 0 | 0 | 0 | 1 | 0 | 1 | Invariable |
| 1 | 200 | 12.253 | 0 | 0 | 2.402 | 1 | 0 | 0.667 | 0 | 1 | Neutral |
| 1 | 201 | 0 | 0 | 1 | 0 | 0 | 0 | 1 | 0 | 1 | Invariable |
| 1 | 202 | 7.218 | 0 | 0 | 1.026 | 1 | 0 | 0.667 | 0 | 1 | Neutral |
| 1 | 203 | 5.519 | 0 | 0 | 0.803 | 1 | 0 | 0.667 | 0 | 1 | Neutral |
| 1 | 204 | 0 | 0 | 1 | 0 | 0 | 0 | 1 | 0 | 1 | Invariable |
| 1 | 205 | 7.532 | 0 | 0 | 9.601 | 1 | 0.012 | 0.639 | 1 | 1 | Neutral |
| 1 | 206 | 14.142 | 0 | 0 | 2.214 | 1 | 0 | 0.667 | 0 | 1 | Neutral |
| 1 | 207 | 7.858 | 0 | 0 | 0.691 | 1 | 0 | 0.667 | 0 | 1 | Neutral |
| 1 | 208 | 0 | 0 | 1 | 0 | 0 | 0 | 1 | 0 | 1 | Invariable |
| 1 | 209 | 9.173 | 0 | 0 | 0.605 | 1 | 0 | 0.667 | 0 | 1 | Neutral |
| 1 | 210 | 20.414 | 0 | 0 | 169.114 | 1 | 0.894 | 0.338 | 1 | 1 | Neutral |
| 1 | 211 | 7.9 | 0 | 0 | 0.378 | 1 | 0 | 0.667 | 0 | 1 | Neutral |
| 1 | 212 | 11.118 | 0 | 0 | 1.665 | 1 | 0 | 0.667 | 0 | 1 | Neutral |
| 1 | 213 | 13.602 | 0 | 0 | 2.93 | 1 | 0 | 0.667 | 0 | 1 | Neutral |
| 1 | 214 | 10.122 | 0 | 0 | 2.572 | 1 | 0 | 0.667 | 0 | 1 | Neutral |
| 1 | 215 | 34.776 | 0 | 0 | 6.135 | 1 | 0 | 0.667 | 0 | 1 | Neutral |
| 1 | 216 | 37.581 | 0 | 0 | 5.623 | 1 | 0 | 0.667 | 0 | 1 | Neutral |
| 1 | 217 | 3.408 | 0 | 0 | 0.542 | 1 | 0 | 0.667 | 0 | 1 | Neutral |
| 1 | 218 | 11.521 | 0 | 0 | 2.42 | 1 | 0 | 0.667 | 0 | 1 | Neutral |
| 1 | 219 | 5.918 | 0 | 0 | 0.887 | 1 | 0 | 0.667 | 0 | 1 | Neutral |
| 1 | 220 | 15.028 | 0 | 0 | 2.055 | 1 | 0 | 0.667 | 0 | 1 | Neutral |
| 1 | 221 | 7.407 | 0 | 0 | 0.959 | 1 | 0 | 0.667 | 0 | 1 | Neutral |
| 1 | 222 | 8.504 | 0 | 0 | 1.59 | 1 | 0 | 0.667 | 0 | 1 | Neutral |
| 1 | 223 | 37.98 | 0 | 0 | 4.33 | 1 | 0 | 0.667 | 0 | 1 | Neutral |
| 1 | 224 | 32.344 | 0 | 0 | 3.141 | 1 | 0 | 0.667 | 0 | 1 | Neutral |
| 1 | 225 | 42.077 | 0 | 0 | 4.669 | 1 | 0 | 0.667 | 0 | 1 | Neutral |
| 1 | 226 | 0 | 0 | 1 | 0 | 0 | 0 | 1 | 0 | 1 | Invariable |
| 1 | 227 | 5.354 | 0 | 0 | 0.821 | 1 | 0 | 0.667 | 0 | 1 | Neutral |
| 1 | 228 | 5.354 | 0 | 0 | 0.821 | 1 | 0 | 0.667 | 0 | 1 | Neutral |
| 1 | 229 | 16.205 | 0 | 0 | 1.293 | 1 | 0 | 0.667 | 0 | 1 | Neutral |
| 1 | 230 | 7.006 | 0 | 0 | 0.693 | 1 | 0 | 0.667 | 0 | 1 | Neutral |
| 1 | 231 | 9.186 | 0 | 0 | 2.061 | 1 | 0 | 0.667 | 0 | 1 | Neutral |
| 1 | 232 | 5.752 | 0 | 0 | 0.841 | 1 | 0 | 0.667 | 0 | 1 | Neutral |
| 1 | 233 | 2.758 | 0 | 0 | 0.263 | 1 | 0 | 0.667 | 0 | 1 | Neutral |
| 1 | 234 | 18.7 | 0 | 0 | 2.609 | 1 | 0 | 0.667 | 0 | 1 | Neutral |
| 1 | 235 | 25.949 | 0 | 0 | 3.108 | 1 | 0 | 0.667 | 0 | 1 | Neutral |
| 1 | 236 | 21.696 | 0 | 0 | 3.136 | 1 | 0 | 0.667 | 0 | 1 | Neutral |
| 1 | 237 | 9.341 | 0 | 0 | 0.614 | 1 | 0 | 0.667 | 0 | 1 | Neutral |
| 1 | 238 | 11 | 0 | 0 | 2.523 | 1 | 0 | 0.667 | 0 | 1 | Neutral |
| 1 | 239 | 0 | 0 | 1 | 0 | 0 | 0 | 1 | 0 | 1 | Invariable |
| 1 | 240 | 11.616 | 0 | 0 | 3.87 | 1 | 0 | 0.667 | 0 | 1 | Neutral |
| 1 | 241 | 7.274 | 0 | 0 | 0.665 | 1 | 0 | 0.667 | 0 | 1 | Neutral |
| 1 | 242 | 9.56 | 0 | 0 | 2.472 | 1 | 0 | 0.667 | 0 | 1 | Neutral |
| 1 | 243 | 5.06 | 1.563 | 0 | 1.267 | 1 | 0 | 0.667 | 0 | 1 | Neutral |
| 1 | 244 | 0 | 0 | 1 | 0 | 0 | 0 | 1 | 0 | 1 | Invariable |
| 1 | 245 | 41.125 | 0 | 0 | 6.748 | 1 | 0 | 0.667 | 0 | 1 | Neutral |
| 1 | 246 | 14.924 | 0 | 0 | 4.29 | 1 | 0 | 0.667 | 0 | 1 | Neutral |
| 1 | 247 | 40.013 | 0 | 0 | 2.014 | 1 | 0 | 0.667 | 0 | 1 | Neutral |
| 1 | 248 | 20.985 | 0 | 0 | 4.111 | 1 | 0 | 0.667 | 0 | 1 | Neutral |
| 1 | 249 | 0 | 0 | 1 | 0 | 0 | 0 | 1 | 0 | 1 | Invariable |
| 1 | 250 | 7.407 | 0 | 0 | 0.959 | 1 | 0 | 0.667 | 0 | 1 | Neutral |
| 1 | 251 | 0 | 0 | 1 | 0 | 0 | 0 | 1 | 0 | 1 | Invariable |
| 1 | 252 | 43.886 | 0 | 0 | 2.545 | 1 | 0 | 0.667 | 0 | 1 | Neutral |
| 1 | 253 | 6.64 | 0 | 0 | 0.966 | 1 | 0 | 0.667 | 0 | 1 | Neutral |
| 1 | 254 | 16.422 | 0 | 0 | 3.026 | 1 | 0 | 0.667 | 0 | 1 | Neutral |
| 1 | 255 | 16.908 | 0 | 0 | 2.905 | 1 | 0 | 0.667 | 0 | 1 | Neutral |
| 1 | 256 | 9.733 | 0 | 0 | 0.763 | 1 | 0 | 0.667 | 0 | 1 | Neutral |
| 1 | 257 | 39.482 | 0 | 0 | 4.034 | 1 | 0 | 0.667 | 0 | 1 | Neutral |
| 1 | 258 | 31.818 | 0 | 0 | 3.642 | 1 | 0 | 0.667 | 0 | 1 | Neutral |
| 1 | 259 | 20.742 | 0 | 0 | 1.873 | 1 | 0 | 0.667 | 0 | 1 | Neutral |
| 1 | 260 | 7.604 | 0 | 0 | 0.597 | 1 | 0 | 0.667 | 0 | 1 | Neutral |
| 1 | 261 | 10.028 | 0 | 0 | 0.825 | 1 | 0 | 0.667 | 0 | 1 | Neutral |
| 1 | 262 | 0 | 0 | 1 | 0 | 0 | 0 | 1 | 0 | 1 | Invariable |
| 1 | 263 | 0 | 0 | 1 | 0 | 0 | 0 | 1 | 0 | 1 | Invariable |
| 1 | 264 | 7.791 | 0 | 0 | 0.778 | 1 | 0 | 0.667 | 0 | 1 | Neutral |
| 1 | 265 | 3.599 | 0 | 0 | 0.595 | 1 | 0 | 0.667 | 0 | 1 | Neutral |
| 1 | 266 | 8.776 | 0 | 0 | 0.755 | 1 | 0 | 0.667 | 0 | 1 | Neutral |
| 1 | 267 | 11.238 | 0 | 0 | 1.268 | 1 | 0 | 0.667 | 0 | 1 | Neutral |
| 1 | 268 | 12.216 | 0 | 0 | 3.986 | 1 | 0 | 0.667 | 0 | 1 | Neutral |
| 1 | 269 | 32.53 | 0 | 0 | 5.147 | 1 | 0 | 0.667 | 0 | 1 | Neutral |
| 1 | 270 | 14.481 | 0 | 0 | 2.246 | 1 | 0 | 0.667 | 0 | 1 | Neutral |
| 1 | 271 | 21.954 | 0 | 0 | 3.501 | 1 | 0 | 0.667 | 0 | 1 | Neutral |
| 1 | 272 | 8.936 | 0 | 0 | 1.996 | 1 | 0 | 0.667 | 0 | 1 | Neutral |
| 1 | 273 | 13.379 | 0 | 0 | 2.14 | 1 | 0 | 0.667 | 0 | 1 | Neutral |
| 1 | 274 | 15.493 | 0 | 0 | 2.757 | 1 | 0 | 0.667 | 0 | 1 | Neutral |
| 1 | 275 | 19.857 | 0 | 0 | 2.923 | 1 | 0 | 0.667 | 0 | 1 | Neutral |
| 1 | 276 | 0 | 0 | 1 | 0 | 0 | 0 | 1 | 0 | 1 | Invariable |
| 1 | 277 | 7.449 | 0 | 0 | 1.066 | 1 | 0 | 0.667 | 0 | 1 | Neutral |
| 1 | 278 | 8.322 | 0 | 0 | 1.083 | 1 | 0 | 0.667 | 0 | 1 | Neutral |
| 1 | 279 | 6.876 | 0 | 0 | 0.989 | 1 | 0 | 0.667 | 0 | 1 | Neutral |
| 1 | 280 | 11.362 | 0 | 0 | 2.379 | 1 | 0 | 0.667 | 0 | 1 | Neutral |
| 1 | 281 | 45.85 | 0 | 0 | 2.643 | 1 | 0 | 0.667 | 0 | 1 | Neutral |
| 1 | 282 | 0.003 | 0.001 | 0 | 3.703 | 1 | 0.3 | 0.491 | 0 | 1 | Neutral |
| 1 | 283 | 8.417 | 0 | 0 | 202.831 | 1 | 1.6 | 0.227 | 1 | 1 | Neutral |
| 1 | 284 | 8.857 | 0 | 0 | 0.306 | 1 | 0 | 0.667 | 0 | 1 | Neutral |
| 1 | 285 | 12.718 | 0 | 0 | 4.098 | 1 | 0 | 0.667 | 0 | 1 | Neutral |
| 1 | 286 | 15.757 | 0 | 0 | 1.54 | 1 | 0 | 0.667 | 0 | 1 | Neutral |
| 1 | 287 | 0 | 0 | 1 | 0 | 0 | 0 | 1 | 0 | 1 | Invariable |
| 1 | 288 | 10.989 | 0 | 0 | 0.875 | 1 | 0 | 0.667 | 0 | 1 | Neutral |
| 1 | 289 | 32.854 | 0 | 0 | 4.169 | 1 | 0 | 0.667 | 0 | 1 | Neutral |
| 1 | 290 | 5.11 | 0 | 0 | 0.522 | 1 | 0 | 0.667 | 0 | 1 | Neutral |
| 1 | 291 | 13.125 | 0 | 0 | 2.653 | 1 | 0 | 0.667 | 0 | 1 | Neutral |
| 1 | 292 | 30.958 | 0 | 0 | 3.396 | 1 | 0 | 0.667 | 0 | 1 | Neutral |
| 1 | 293 | 14.43 | 0 | 0 | 2.577 | 1 | 0 | 0.667 | 0 | 1 | Neutral |
| 1 | 294 | 64.445 | 0 | 0 | 10.8 | 1 | 0 | 0.667 | 0 | 1 | Neutral |
| 1 | 295 | 0 | 0 | 1 | 0 | 0 | 0 | 1 | 0 | 1 | Invariable |
| 1 | 296 | 0 | 0 | 1 | 0 | 0 | 0 | 1 | 0 | 1 | Invariable |
| 1 | 297 | 39.772 | 0 | 0 | 3.499 | 1 | 0 | 0.667 | 0 | 1 | Neutral |
| 1 | 298 | 8.132 | 0 | 0 | 1.892 | 1 | 0 | 0.667 | 0 | 1 | Neutral |
| 1 | 299 | 4.918 | 0 | 0 | 0.769 | 1 | 0 | 0.667 | 0 | 1 | Neutral |
| 1 | 300 | 5.519 | 0 | 0 | 0.841 | 1 | 0 | 0.667 | 0 | 1 | Neutral |
| 1 | 301 | 0 | 0 | 1 | 0 | 0 | 0 | 1 | 0 | 1 | Invariable |
| 1 | 302 | 10.898 | 0 | 0 | 2.197 | 1 | 0 | 0.667 | 0 | 1 | Neutral |
| 1 | 303 | 0 | 0 | 1 | 0 | 0 | 0 | 1 | 0 | 1 | Invariable |
| 1 | 304 | 12.632 | 0 | 0 | 1.492 | 1 | 0 | 0.667 | 0 | 1 | Neutral |
| 1 | 305 | 3.317 | 0 | 0 | 0.554 | 1 | 0 | 0.667 | 0 | 1 | Neutral |
| 1 | 306 | 3.408 | 0 | 0 | 0.542 | 1 | 0 | 0.667 | 0 | 1 | Neutral |
| 1 | 307 | 7.877 | 0 | 0 | 1.091 | 1 | 0 | 0.667 | 0 | 1 | Neutral |
| 1 | 308 | 21.866 | 0 | 0 | 5.455 | 1 | 0 | 0.667 | 0 | 1 | Neutral |
| 1 | 309 | 3.18 | 0 | 0 | 0.32 | 1 | 0 | 0.667 | 0 | 1 | Neutral |
| 1 | 310 | 0 | 0 | 1 | 0 | 0 | 0 | 1 | 0 | 1 | Invariable |
| 1 | 311 | 9.919 | 0 | 0 | 1.309 | 1 | 0 | 0.667 | 0 | 1 | Neutral |
| 1 | 312 | 9.445 | 0 | 0 | 3.323 | 1 | 0 | 0.667 | 0 | 1 | Neutral |
| 1 | 313 | 0 | 0 | 1 | 0 | 0 | 0 | 1 | 0 | 1 | Invariable |
| 1 | 314 | 2.706 | 0 | 0 | 0.287 | 1 | 0 | 0.667 | 0 | 1 | Neutral |
| 1 | 315 | 0 | 0 | 1 | 0 | 0 | 0 | 1 | 0 | 1 | Invariable |
| 1 | 316 | 0 | 0 | 1 | 0 | 0 | 0 | 1 | 0 | 1 | Invariable |
| 1 | 317 | 4.002 | 0 | 0 | 0.621 | 1 | 0 | 0.667 | 0 | 1 | Neutral |
| 1 | 318 | 20.56 | 0 | 0 | 3.253 | 1 | 0 | 0.667 | 0 | 1 | Neutral |
| 1 | 319 | 12.546 | 0 | 0 | 2.563 | 1 | 0 | 0.667 | 0 | 1 | Neutral |
| 1 | 320 | 20.476 | 0 | 0 | 3.246 | 1 | 0 | 0.667 | 0 | 1 | Neutral |
| 1 | 321 | 36.991 | 4.009 | 0 | 3.767 | 1 | 0 | 0.667 | 0 | 1 | Neutral |
| 1 | 322 | 6.619 | 0 | 0 | 0.769 | 1 | 0 | 0.667 | 0 | 1 | Neutral |
| 1 | 323 | 3.982 | 0 | 0 | 0.647 | 1 | 0 | 0.667 | 0 | 1 | Neutral |
| 1 | 324 | 34.993 | 0 | 0 | 5.179 | 1 | 0 | 0.667 | 0 | 1 | Neutral |
| 1 | 325 | 15.181 | 0 | 0 | 4.616 | 1 | 0 | 0.667 | 0 | 1 | Neutral |
| 1 | 326 | 8.869 | 0 | 0 | 0.411 | 1 | 0 | 0.667 | 0 | 1 | Neutral |
| 1 | 327 | 22.664 | 0 | 0 | 1.586 | 1 | 0 | 0.667 | 0 | 0.998 | Neutral |
| 1 | 328 | 15.537 | 0 | 0 | 0.206 | 1 | 0 | 0.667 | 0 | 0.994 | Neutral |
| 1 | 329 | 13.505 | 0 | 0 | 3.245 | 1 | 0 | 0.667 | 0 | 0.991 | Neutral |
| 1 | 330 | 15.803 | 0 | 0 | 3.383 | 1 | 0 | 0.667 | 0 | 0.987 | Neutral |
| 1 | 331 | 3.846 | 0 | 0 | 0.629 | 1 | 0 | 0.667 | 0 | 0.984 | Neutral |
| 1 | 332 | 10.908 | 0 | 0 | 2.344 | 1 | 0 | 0.667 | 0 | 0.98 | Neutral |
| 1 | 333 | 7.723 | 0 | 0 | 1.003 | 1 | 0 | 0.667 | 0 | 0.977 | Neutral |
| 1 | 334 | 5.996 | 0 | 0 | 1.643 | 1 | 0 | 0.667 | 0 | 0.973 | Neutral |
| 1 | 335 | 19.375 | 0 | 0 | 2.889 | 1 | 0 | 0.667 | 0 | 0.97 | Neutral |
| 1 | 336 | 9.921 | 0 | 0 | 1.97 | 1 | 0 | 0.667 | 0 | 0.967 | Neutral |
| 1 | 337 | 7.863 | 0 | 0 | 0.348 | 1 | 0 | 0.667 | 0 | 0.963 | Neutral |
| 1 | 338 | 2.835 | 0 | 0 | 0.484 | 1 | 0 | 0.667 | 0 | 0.96 | Neutral |
| 1 | 339 | 0.004 | 0 | 0 | 1.255 | 1 | 0.313 | 0.486 | 0 | 1 | Neutral |
| 1 | 340 | 3.016 | 0 | 0 | 0.509 | 1 | 0 | 0.667 | 0 | 0.957 | Neutral |
| 1 | 341 | 4.045 | 0 | 0 | 0.627 | 1 | 0 | 0.667 | 0 | 0.954 | Neutral |
| 1 | 342 | 59.955 | 0 | 0 | 1.578 | 1 | 0 | 0.667 | 0 | 0.95 | Neutral |
| 1 | 343 | 9.444 | 0 | 0 | 1.133 | 1 | 0 | 0.667 | 0 | 0.947 | Neutral |
| 1 | 344 | 0 | 0 | 1 | 0 | 0 | 0 | 1 | 0 | 1 | Invariable |
| 1 | 345 | 1.95 | 0 | 0 | 0.267 | 1 | 0 | 0.667 | 0 | 0.944 | Neutral |
| 1 | 346 | 8.622 | 0 | 0 | 0.552 | 1 | 0 | 0.667 | 0 | 0.941 | Neutral |
| 1 | 347 | 6.189 | 0 | 0 | 0.647 | 1 | 0 | 0.667 | 0 | 0.938 | Neutral |
| 1 | 348 | 0 | 0 | 1 | 0 | 0 | 0 | 1 | 0 | 1 | Invariable |
| 1 | 349 | 14.481 | 0 | 0 | 2.246 | 1 | 0 | 0.667 | 0 | 0.935 | Neutral |
| 1 | 350 | 14.641 | 0 | 0 | 2.306 | 1 | 0 | 0.667 | 0 | 0.932 | Neutral |
| 1 | 351 | 5.547 | 0 | 0 | 0.843 | 1 | 0 | 0.667 | 0 | 0.928 | Neutral |
| 1 | 352 | 20.624 | 0 | 0 | 3.388 | 1 | 0 | 0.667 | 0 | 0.925 | Neutral |
| 1 | 353 | 3.493 | 0 | 0 | 0.579 | 1 | 0 | 0.667 | 0 | 0.922 | Neutral |
| 1 | 354 | 7.04 | 0.045 | 0 | 4.643 | 1 | 0 | 0.667 | 0 | 0.919 | Neutral |
| 1 | 355 | 7.858 | 0 | 0 | 0.691 | 1 | 0 | 0.667 | 0 | 0.916 | Neutral |
| 1 | 356 | 0 | 0 | 1 | 0 | 0 | 0 | 1 | 0 | 1 | Invariable |
| 1 | 357 | 10.626 | 0 | 0 | 2.77 | 1 | 0 | 0.667 | 0 | 0.913 | Neutral |
| 1 | 358 | 6.932 | 0 | 0 | 0.996 | 1 | 0 | 0.667 | 0 | 0.91 | Neutral |
| 1 | 359 | 15.749 | 0 | 0 | 2.18 | 1 | 0 | 0.667 | 0 | 0.908 | Neutral |
| 1 | 360 | 2.654 | 0 | 0 | 6.988 | 1 | 0.194 | 0.53 | 1 | 1 | Neutral |
| 1 | 361 | 8.863 | 1.527 | 0 | 1.68 | 1 | 0 | 0.667 | 0 | 0.905 | Neutral |
| 1 | 362 | 24.752 | 0 | 0 | 7.38 | 1 | 0 | 0.667 | 0 | 0.902 | Neutral |
| 1 | 363 | 34.329 | 0 | 0 | 5.209 | 1 | 0 | 0.667 | 0 | 0.899 | Neutral |
| 1 | 364 | 7.979 | 0 | 0 | 0.613 | 1 | 0 | 0.667 | 0 | 0.896 | Neutral |
| 1 | 365 | 32.468 | 1.57 | 0 | 4.334 | 1 | 0 | 0.667 | 0 | 0.893 | Neutral |
| 1 | 366 | 0.001 | 0 | 0 | 1.293 | 1 | 0.465 | 0.439 | 0 | 1 | Neutral |
| 1 | 367 | 14.481 | 0 | 0 | 2.246 | 1 | 0 | 0.667 | 0 | 0.89 | Neutral |
| 1 | 368 | 0.002 | 0 | 0 | 1.283 | 1 | 0.517 | 0.425 | 1 | 1 | Neutral |
| 1 | 369 | 0 | 0 | 1 | 0 | 0 | 0 | 1 | 0 | 1 | Invariable |
| 1 | 370 | 25.095 | 0 | 0 | 6.093 | 1 | 0 | 0.667 | 0 | 0.887 | Neutral |
| 1 | 371 | 8.561 | 0 | 0 | 0.702 | 1 | 0 | 0.667 | 0 | 0.885 | Neutral |
| 1 | 372 | 50.826 | 0 | 0 | 8.056 | 1 | 0 | 0.667 | 0 | 0.882 | Neutral |
| 1 | 373 | 7.636 | 0 | 0 | 1.068 | 1 | 0 | 0.667 | 0 | 0.879 | Neutral |
| 1 | 374 | 3.317 | 0 | 0 | 0.554 | 1 | 0 | 0.667 | 0 | 0.876 | Neutral |
| 1 | 375 | 10.963 | 1.614 | 0 | 1.564 | 1 | 0 | 0.667 | 0 | 0.874 | Neutral |
| 1 | 376 | 18.656 | 0 | 0 | 4.091 | 1 | 0 | 0.667 | 0 | 0.871 | Neutral |
| 1 | 377 | 3.846 | 0 | 0 | 0.629 | 1 | 0 | 0.667 | 0 | 0.868 | Neutral |
| 1 | 378 | 22.202 | 0 | 0 | 3.548 | 1 | 0 | 0.667 | 0 | 0.866 | Neutral |
| 1 | 379 | 0 | 0 | 1 | 0 | 0 | 0 | 1 | 0 | 1 | Invariable |
| 1 | 380 | 1.95 | 0 | 0 | 0.267 | 1 | 0 | 0.667 | 0 | 0.863 | Neutral |
| 1 | 381 | 3.376 | 0 | 0 | 0.839 | 1 | 0 | 0.667 | 0 | 0.86 | Neutral |
| 1 | 382 | 0 | 0 | 1 | 0 | 0 | 0 | 1 | 0 | 1 | Invariable |
| 1 | 383 | 0 | 0 | 1 | 0 | 0 | 0 | 1 | 0 | 1 | Invariable |
| 1 | 384 | 22.486 | 0 | 0 | 4.237 | 1 | 0 | 0.667 | 0 | 0.858 | Neutral |
| 1 | 385 | 5.629 | 0 | 0 | 0.854 | 1 | 0 | 0.667 | 0 | 0.855 | Neutral |
| 1 | 386 | 26.479 | 1.58 | 0 | 2.743 | 1 | 0 | 0.667 | 0 | 0.853 | Neutral |
| 1 | 387 | 0 | 0 | 1 | 0 | 0 | 0 | 1 | 0 | 1 | Invariable |
| 1 | 388 | 0 | 0 | 1 | 0 | 0 | 0 | 1 | 0 | 1 | Invariable |
| 1 | 389 | 6.366 | 0 | 0 | 0.936 | 1 | 0 | 0.667 | 0 | 0.85 | Neutral |
| 1 | 390 | 3.44 | 1.707 | 0 | 1.691 | 1 | 0 | 0.667 | 0 | 0.847 | Neutral |
| 1 | 391 | 4.511 | 0 | 0 | 0.717 | 1 | 0 | 0.667 | 0 | 0.845 | Neutral |
| 1 | 392 | 9.451 | 0 | 0 | 0.793 | 1 | 0 | 0.667 | 0 | 0.842 | Neutral |
| 1 | 393 | 0 | 0 | 1 | 0 | 0 | 0 | 1 | 0 | 1 | Invariable |
| 1 | 394 | 0 | 0 | 1 | 0 | 0 | 0 | 1 | 0 | 1 | Invariable |
| 1 | 395 | 18.631 | 0 | 0 | 2.604 | 1 | 0 | 0.667 | 0 | 0.84 | Neutral |
| 1 | 396 | 3.004 | 0 | 0 | 0.51 | 1 | 0 | 0.667 | 0 | 0.837 | Neutral |
| 1 | 397 | 32.545 | 0 | 0 | 3.682 | 1 | 0 | 0.667 | 0 | 0.835 | Neutral |
| 1 | 398 | 15.456 | 0 | 0 | 1.838 | 1 | 0 | 0.667 | 0 | 0.832 | Neutral |
| 1 | 399 | 6.932 | 0 | 0 | 0.996 | 1 | 0 | 0.667 | 0 | 0.83 | Neutral |
| 1 | 400 | 5.832 | 0 | 0 | 0.374 | 1 | 0 | 0.667 | 0 | 0.827 | Neutral |
| 1 | 401 | 14.725 | 0 | 0 | 2.662 | 1 | 0 | 0.667 | 0 | 0.825 | Neutral |
| 1 | 402 | 11.145 | 0 | 0 | 0.883 | 1 | 0 | 0.667 | 0 | 0.823 | Neutral |
| 1 | 403 | 4.675 | 0 | 0 | 0.738 | 1 | 0 | 0.667 | 0 | 0.82 | Neutral |
| 1 | 404 | 6.269 | 0 | 0 | 0.83 | 1 | 0 | 0.667 | 0 | 0.818 | Neutral |
| 1 | 405 | 9.186 | 1.78 | 0 | 1.905 | 1 | 0 | 0.667 | 0 | 0.815 | Neutral |
| 1 | 406 | 3.252 | 0 | 0 | 0.544 | 1 | 0 | 0.667 | 0 | 0.813 | Neutral |
| 1 | 407 | 8.508 | 0 | 0 | 1.636 | 1 | 0 | 0.667 | 0 | 0.811 | Neutral |
| 1 | 408 | 3.591 | 0 | 0 | 0.387 | 1 | 0 | 0.667 | 0 | 0.808 | Neutral |
| 1 | 409 | 21.492 | 0 | 0 | 3.335 | 1 | 0 | 0.667 | 0 | 0.806 | Neutral |
| 1 | 410 | 41.17 | 0 | 0 | 4.496 | 1 | 0 | 0.667 | 0 | 0.804 | Neutral |
| 1 | 411 | 20.624 | 0 | 0 | 3.388 | 1 | 0 | 0.667 | 0 | 0.802 | Neutral |
| 1 | 412 | 29.882 | 0 | 0 | 3.975 | 1 | 0 | 0.667 | 0 | 0.799 | Neutral |
| 1 | 413 | 19.51 | 1.363 | 0 | 1.643 | 1 | 0 | 0.667 | 0 | 0.797 | Neutral |
| 1 | 414 | 32.306 | 0 | 0 | 4.005 | 1 | 0 | 0.667 | 0 | 0.795 | Neutral |
| 1 | 415 | 33.821 | 0 | 0 | 3.752 | 1 | 0 | 0.667 | 0 | 0.792 | Neutral |
| 1 | 416 | 0 | 0 | 1 | 0 | 0 | 0 | 1 | 0 | 1 | Invariable |
| 1 | 417 | 0 | 0 | 1 | 0 | 0 | 0 | 1 | 0 | 1 | Invariable |
| 1 | 418 | 7.317 | 0 | 0 | 1.053 | 1 | 0 | 0.667 | 0 | 0.79 | Neutral |
| 1 | 419 | 14.315 | 1.492 | 0 | 1.432 | 1 | 0 | 0.667 | 0 | 0.788 | Neutral |
| 1 | 420 | 0 | 0 | 1 | 0 | 0 | 0 | 1 | 0 | 1 | Invariable |
| 1 | 421 | 42.766 | 0 | 0 | 4.36 | 1 | 0 | 0.667 | 0 | 0.786 | Neutral |
| 1 | 422 | 5.481 | 0 | 0 | 0.836 | 1 | 0 | 0.667 | 0 | 0.784 | Neutral |
