# Supplementary material for: Unravelling the genomic epidemiology and antigenic evolution of Chandipura virus: A genetically constrained neurotropic threat
Source: PLoS Negl Trop Dis. 2026 Aug 3;20(8):e0014565. doi: 10.1371/journal.pntd.0014565 (PMC13489527; doi:10.1371/journal.pntd.0014565)
Supplement: Supplementary file 8 [file pntd.0014565.s008.docx]

**S1 Fig. Temporal signal analysis of Chandipura virus (CHPV) sequences using TempEst. Root-to-tip regression plots showing the correlation between genetic divergence and sampling time for CHPV sequences.** Panels depict: (A) whole genome, (B) glycoprotein (G), (C) matrix protein (M), (D) nucleoprotein (N), (E) phosphoprotein (P), and (F) large protein (L). Temporal signal was assessed to evaluate the suitability of the datasets for molecular clock analyses, with root-to-tip correlation coefficients indicated for each panel.
