# Supplementary material for: Unravelling the genomic epidemiology and antigenic evolution of Chandipura virus: A genetically constrained neurotropic threat
Source: PLoS Negl Trop Dis. 2026 Aug 3;20(8):e0014565. doi: 10.1371/journal.pntd.0014565 (PMC13489527; doi:10.1371/journal.pntd.0014565)
Supplement: Supplementary file 9 [file pntd.0014565.s009.docx]

**S2 Fig. Bayesian skyline plots of Chandipura virus protein-coding genes inferred under a relaxed molecular clock model.** Bayesian skyline reconstructions of effective population size (N_e_τ) for individual Chandipura virus (CHPV) protein-coding genes inferred under an uncorrelated lognormal relaxed molecular clock model using BEAST 2.7.7. Panels represent: (A) glycoprotein (G), (B) matrix protein (M), (C) nucleoprotein (N), (D) phosphoprotein (P), and (E) large polymerase protein (L). In each panel, the solid line represents the median estimate, and the shaded region indicates the 95% highest posterior density (HPD) interval. The x-axis denotes time (years), and the y-axis represents scaled effective population size (N_e_τ).
