# Supplementary material for: Unravelling the genomic epidemiology and antigenic evolution of Chandipura virus: A genetically constrained neurotropic threat
Source: PLoS Negl Trop Dis. 2026 Aug 3;20(8):e0014565. doi: 10.1371/journal.pntd.0014565 (PMC13489527; doi:10.1371/journal.pntd.0014565)
Supplement: Supplementary file 10 [file pntd.0014565.s010.docx]

**S3 Fig. Bayesian skyline plots of Chandipura virus genomes and protein-coding genes inferred under a strict molecular clock model.** Bayesian skyline reconstructions of effective population size (N_e_τ) for the Chandipura virus (CHPV) whole genome and individual protein-coding genes inferred under a strict molecular clock model using BEAST 2.7.7. Panels represent: (A) whole genome, (B) glycoprotein (G), (C) matrix protein (M), (D) nucleoprotein (N), (E) phosphoprotein (P), and (F) large polymerase protein (L). In each panel, the solid line represents the median estimate, and the shaded region indicates the 95% highest posterior density (HPD) interval. The x-axis denotes time (years), and the y-axis represents scaled effective population size (N_e_τ).
